# Mood in the moment: a study protocol for embedding ecological momentary assessments into established longitudinal cohorts to examine depression in real time

**DOI:** 10.1136/bmjopen-2026-122195

**Published:** 2026-06-23

**Authors:** Alex S F Kwong, Sarah Moody, Adele Taylor, Celestine Lockhart, Rachel Ogden, Ray Leal, Andrew Mcmillan, Jessica Harvey, Elizabeth Brierley, Sarah Matthews, Richard Hobbs, Anne Simmons, Thalia C Eley, Nicholas C Jacobson, Aja L Murray

**Affiliations:** 1 Division of Psychiatry, Centre for Clinical Brain Sciences, The University of Edinburgh, Edinburgh, UK; 2 Population Health Sciences, Bristol Medical School, University of Bristol, Bristol, UK; 3 MRC Integrative Epidemiology Unit, University of Bristol, Bristol, UK; 4 Social, Genetic and Developmental Psychiatry, King’s College London, London, UK; 5 South London and Maudsley NHS Foundation Trust, National Institute for Health and Care Research (NIHR) Maudsley Biomedical Research Centre, London, UK; 6 Center for Technology and Behavioral Health, Geisel School of Medicine, Dartmouth College, Hanover, New Hampshire, USA; 7 Department of Psychology, The University of Edinburgh, Edinburgh, UK

**Keywords:** Depression & mood disorders, Longitudinal studies, Child & adolescent psychiatry, Adult psychiatry, STATISTICS & RESEARCH METHODS

## Abstract

**Introduction:**

Depression is a major global health challenge, with onset commonly occurring in youth. There is an urgent need to better understand the epidemiology of depression to facilitate better interventions and preventions that are person-specific and time-specific. Long-term depression trajectories and ecological momentary assessment (EMA) can help address this need. Embedding EMA designs within established longitudinal cohorts offers a uniquely powerful approach to examine depression in both long- and short-term settings and to identify distal and proximal modifiable risk factors for depression.

**Methods and analyses:**

Our study will include ~450 participants from the Twins Early Development Study and ~250 participants from the Avon Longitudinal Study of Parents and Children (ALSPAC) who have depression trajectories data previously collected between late childhood and early adulthood. Participants will be recruited from four different depression trajectories in each cohort based on participants’ prior symptoms. They will undertake EMA surveys of depression, sleep, physical activity, substance use, diet, recent activities and social interactions three times a day for 6 weeks, with more detailed questionnaires at baseline, 2, 4 and 6 weeks. We will use descriptive analysis, mixed effects models and dynamic structural equation modelling to examine how depression occurs over this time, how potentially modifiable factors affect depression and vice versa and how these effects vary by different life-course trajectories. This study is designed to identify people at heightened risk of depression and/or identify modifiable targets that could inform more effective prevention and intervention strategies.

**Ethics and dissemination:**

Favourable ethical opinions were given by the Edinburgh Medical School Research Ethics Committee (REC References: 25-EMREC-001 and 25-EMREC-030) and the ALSPAC Law and Ethics Committee (Ref: 0027 B4792). The results will be disseminated through journal publications, conferences and seminar presentations and to relevant stakeholders, such as those with a history of depression, policy makers and clinicians.

**STRENGTHS AND LIMITATIONS OF THIS STUDY**

- This study will combine intensive longitudinal data (ecological momentary assessment [EMA]) with traditional longitidinal data to examine depression in both the long and short term, for the first time.
- This study has a large sample of 700 participants who will complete complete brief EMAs up to three times a day, for 6 weeks, with additional baseline and fortnightly questionnaires.
- A range of measures will be used during the EMA study, allowing for the identification of important modifiable factors related to depression in real time, for specific groups of individuals with different depression histories.
- In Avon Longitudinal Study of Parents and Children, a subset of participants will also wear smartwatches to passively collect data on sleep, activity and heart rate.
- Attrition is likely to play a role in the EMA data collection, especially for participants with a history of depression.

## Introduction

Depression is a global health challenge, affecting over an estimated 330 million people worldwide,^1^ and predicted to be the leading cause of disability by 2030.^2^ Young people are at particular risk of depression,^3^ with emerging evidence that depression is worsening within this population.^4 5^ Depression during this period is associated with wide-ranging and long-lasting economic, social and physiological consequences.^6–9^ Evidence suggests that 50% of people who have ever had depression had an onset before the age of 25,^10^ and almost 50% of those who develop depression between 18 and 40 will have a subsequent episode.^11^ This makes it crucial to prevent or limit depression during earlier life to mitigate later effects. Understanding what modifiable factors and mechanisms influence the course of depression is therefore of considerable clinical, economic and societal importance. Such research could ultimately help identify people at heightened risk of worsening depression and/or identify modifiable targets that could inform more effective prevention and intervention strategies.

Longitudinal studies that track the same people over time are critical to addressing this line of research. Such studies can estimate depression trajectories across development and highlight how depression develops, persists and remits across the life course, as well as what trajectories are specific for different aetiologies and outcomes.^12–14^ Importantly, they have also identified key risk and protective factors such as sex and pubertal timing,^15 16^ genetic vulnerability,^17 18^ early adversity^7^ and the family environment^19 20^ that likely shape depression over time. While such research is important, these studies typically assess depression at yearly intervals or longer. As such, there is a current paucity for what depression looks like in between these assessments, and how depression and its correlates may fluctuate and drive one another in the short-term, from day-to-day or even moment to moment settings. A greater understanding of more granular short-term manifestation could inform treatment provision and illuminate the perpetuating or proximal mechanisms that drive depression in day-to-day life.

Ecological momentary assessment (EMA) has emerged as a powerful tool for addressing this limitation.^21–23^ EMA designs can collect data multiple times a day while participants go about their daily routines, enabling researchers to examine depression as it unfolds in naturalistic settings and to identify how specific contextual factors can shape mood and vice versa, all in close to real time.^24 25^ EMA designs can be paired with passive data collection (ie, through smartwatches) to provide objective measures of factors such as sleep, exercise or heart rate and their relation to daily mood^26^ in a continuous, non-invasive manner. EMA is particularly well suited to capturing the dynamic, person and context-dependent nature of depression, providing the opportunity to measure short-term depression trajectories over a given period. It can also capture symptom variability, the occurrence of correlated factors and perhaps most importantly, reactivity to daily stressors and life events in a way that retrospective designs cannot reliably detect. A growing body of EMA research has demonstrated that short-term fluctuations in depression are both clinically meaningful and informative.^27^ For example, EMA designs have shown that depression is not stable, even across a single day.^28^ Furthermore, momentary factors such as disrupted sleep,^28 29^ reduced physical activity^28 30 31^ and worse social interactions are believed to influence depressive states in the short term.^24 32 33^ However, existing EMA studies of depression have predominantly been conducted in small samples (often clinical populations or student cohorts), without access to broader phenotypic or prospectively ascertained life-course data. This limits their capacity to examine how short-term depressive dynamics fit within the broader context of development and the life course.

To date, long-term trajectory research and short-term intensive data collection have not been conducted in depression research, with only a handful of studies embedding broader EMA designs into longitudinal studies, such as in ADHD research.^34^ This represents a significant missed opportunity to combine detailed data on depression in the moment and short-term depression trajectories, alongside detailed longer-term depression trajectories derived from routinely collected data. Short-term depression symptoms and the modifiable factors that drive them such as sleep, exercise, diet, substance use and social interactions could illuminate proximal mechanisms that underpin longer-term depression trajectories—thus highlighting opportunities for more tailored and targeted interventions and preventions that are time-specific and person-specific. Embedding EMA within established longitudinal cohorts, whose participants have well-characterised life-course depression trajectories, offers a uniquely powerful approach to address this gap. By selecting participants based on previously characterised depression trajectories, it is possible to examine the real-time epidemiology of depression and how it varies across individuals with different histories of depressive experience. This can also be combined with data collected across development, such as psychosocial factors, family history and genetic risk, to identify the momentary risk and protective factors that are most relevant within different trajectories.

In this study, we will use an EMA design embedded within two established longitudinal studies: the Twins Early Development Study (TEDS) and the Avon Longitudinal Study of Parents and Children (ALSPAC) to study depression in both the long- and short-term. Both studies have extensive life-course data on depression and its determinants, allowing us to derive clinically meaningful and robust depression trajectories from childhood to early/emerging adulthood. The nature of this study will allow us to examine how short-term depressive dynamics occur and vary within the context of longer-term depression trajectories and how momentary risk and protective factors, such as sleep, physical activity, diet, substance use, social interactions and stress, contribute to these fluctuations in real time. Furthermore, in a subsample of ALSPAC participants, concurrent passive data collection will be collected via wearable smartwatch devices to enrich this picture by providing objective physiological measures of sleep, physical activity and heart rate, complementing and contextualising the self-reported EMA data.^35 36^ Our core research questions are:

- (RQ1) How does depression occur and vary in the short-term—from day-to-day or even moment-to-moment settings in people with different prior life-course depression trajectories?
- (RQ2) Which momentary risk and protective factors such as sleep, stress, physical activity, diet, substance use and social interactions are most strongly associated with short-term fluctuations and symptoms in depression in daily life, and how do these vary by different depression trajectories?
- (RQ3) How do short-term depression dynamics relate to longer-term depression trajectories previously characterised in the cohort, and do they mediate or moderate the associations between established risk factors and longer-term outcomes?

Exploratory analyses may be used to address a range of additional questions, including examination of individual differences in the links between momentary risk factors and depression, using machine learning to predict greater levels of depression and to identify further potential targets for personalised and timely intervention. For more ambitious questions like RQ3, we will pre-register analysis on the open science framework.

## Methods and analysis

### Existing study participants

Participants will be drawn from two existing longitudinal cohort studies: TEDS and the ALSPAC. In TEDS, the original sample comprised 13 759 families with newborn twins, who participated in the first assessment wave at 18 months. Families were recruited from the Office of National Statistics’ national register of twins born in England and Wales between 1994 and 1996.^37^ In ALSPAC, pregnant women residing in Avon, UK with expected delivery between 1 April 1991 and 31 December 1992 were invited to take part in the study. A total of 14 901 children alive at 1 year of age participated in ALSPAC.^38–40^ Further cohort information is provided in the online supplemental materials. Overall ethical approval for TEDS was granted by the King’s College London Research Ethics Committee (References: PNM/09/10–104, HR/DP-20/2122060, and RESCM-24/25-22060), and consent was given by participants at each wave. In ALSPAC, overall ethical approval for the study was obtained from the ALSPAC Ethics and Law Committee and the Local Research Ethics Committees. Informed consent for the use of existing data collected was obtained from participants following the recommendations of the ALSPAC Ethics and Law Committee at the time. Participants can contact the study team at any time to retrospectively withdraw consent for their data to be used. Study participation is voluntary and during all data collection sweeps, information was provided on the intended use of data. In TEDS, the study website contains details of all data that is available: https://datadictionary.teds.ac.uk/home.htm. In ALSPAC, the study website contains details of all data that is available through a fully searchable data dictionary and variable search tool and reference the following webpage: http://www.bristol.ac.uk/alspac/researchers/our-data/.

10.1136/bmjopen-2026-122195.supp1 Supplementary data

### EMA participants

**Figure 1:**
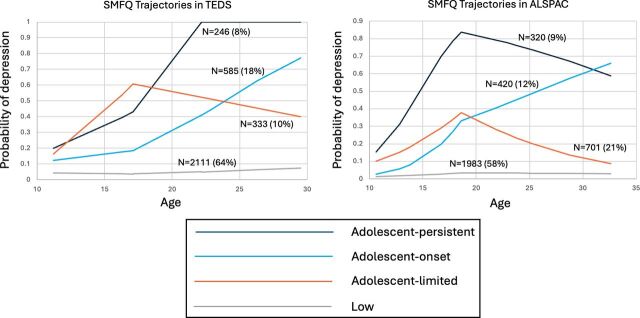
Depression trajectories in each cohort. N (and percentage of total sample) are given for each trajectory. ALSPAC, Avon Longitudinal Study of Parents and Children; SMFQ, Short Mood and Feelings Questionnaire; TEDS, Twins Early Development Study.

**Figure 2:**
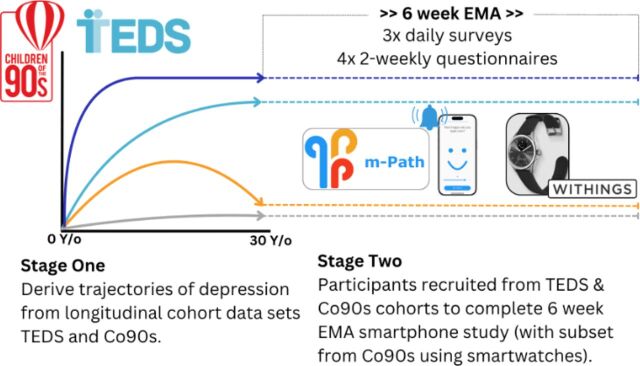
Summary of the mood in the moment study design with schematic depression trajectories. Co90s, children of the 90s; EMA, ecological momentary assessment; TEDS, Twins Early Development Study.

The present study will include ~450 participants from TEDS and ~250 participants from ALSPAC who have depression trajectories data previously collected between late childhood and early adulthood. We will only recruit participants who have responded to the most recent questionnaire on depression within each study (both assessed in 2025) to ensure that participants belong to the most up to date depression trajectories. Participants in the EMA study will have a mean age of 29.5 years in TEDS and 32.6 years in ALSPAC. All participants will be required to have completed a minimum of two depression assessments (one in childhood/adolescence and another in late adolescence/early adulthood) to be included in the initial trajectory analysis in which to recruit from. Based on recent questionnaire data collected in both cohorts, we have a maximum sample of 3275 participants in TEDS and 3866 participants in ALSPAC from which to recruit participants into the EMA section of the study. Based on previous research,^7 41^ we estimated four similar trajectories per cohort (a stable low trajectory, an adolescent-onset trajectory, an adolescent-limited trajectory and a persistent trajectory), see figure 1. We will recruit equally from each trajectory group to ensure a matched case-control analysis, randomising the order in which we invite participants to join the EMA study. In ALSPAC, ~100 participants will be given Withings smartwatches to passively collect data on sleep, activity and heart rate throughout the study. Figure 2 shows a graphical representation of our study.

### Inclusion criteria

The inclusion criteria for this study require participants to be active and consenting participants of the TEDS and ALSPAC studies and have previously measured data on depression (ie, a minimum of two depression assessments (one in childhood/adolescence and another in late adolescence/early adulthood)) and have completed the most recent questionnaire on mental health. Eligible participants will be invited to join the study if they meet these criteria and will be invited by the TEDS and ALSPAC teams directly through existing contact information held by the studies. In TEDS, only participants who have consented to be contacted about future substudies will be invited to take part. Participants will be required to have a sufficient understanding of written and spoken English language to understand study information and instructions, provide informed consent and participate in the text-based EMA surveys to be completed via smartphone application. Participants will also be required to own a smartphone device with access to either the app store (Apple iPhones) or Google Play Store (Android phones) to download the smartphone application m-Path to allow participation in the study for a period of six consecutive weeks. In ALSPAC, participants must be willing and able to download the Withings smartwatch application and wear a Withings smartwatch for a period of 6 weeks (if consented to this aspect of the study). Across both studies, participants must be available to participate in the study for a consecutive 6 week period without significant breaks while the data collection is live.

### Patient and public involvement

Individuals with and without depression have contributed to the design of this study. We first consulted people with lived experience of depression as part of an ongoing user-led citizen science project called ‘The Depression Detectives’. In their capacity as advisors, they saw value in identifying patterns of depression early through smartphones and to identify key factors that could prevent depression from getting worse and leading to long-term problems. Next, a group of adolescents recruited as part of a patient and public lived experience group determined feasibility of the study, recommending the types of factors to include and duration of the study. Finally, active participants from the ALSPAC study provided guidance on the duration of the study, measures used and amount of reimbursement that would be suitable for the study.

### Data collection

Data collection began in September 2025 and is expected to complete by August 2026. Analysis will be staggered across this period to avoid seasonality effects. Figure 2 shows a graphical representation of our study, where participants will be recruited based on their existing depression trajectories. Participants will then be invited to join this EMA study and provide consent as described above.

Participants who give consent and begin the study will be sent instructions on how to download the ‘M-Path’ EMA smartphone application. In ALSPAC, ~100 random (equal across trajectories) participants will be recruited into the smartwatch element of the study will also be sent instructions on how to download the ‘Withings’ smartphone application. Each participant will be sent their own unique and pseudo-anonymised login that only the respective study teams can use to identify participants.

Participants who successfully join the study will first complete a baseline questionnaire and have 7 days to complete this before they are removed from the study. Completion of this baseline questionnaire will initiate EMA surveys starting the next morning. EMA surveys will occur three times a day (randomly in the morning (08:00 to 09:00), afternoon (13:00 to 14:00) and evening (18:00 to 19:00)) for 6 weeks (for a maximum of 126 surveys), all through the ‘M-Path’ smartphone application. These EMA surveys are designed to be completed in under 3 min, and each daily survey is available for 3 hours from the initial prompt. Reminders were sent after 60 and 120 min. Slightly longer questionnaires (taking ~5 min to complete) are also available to complete at the 2-week and 4-week intervals. A final end-of-study questionnaire is then available to complete at the end of the study at week 6. Participants will be reimbursed £1 for every EMA survey they complete, and £2 for each questionnaire they complete (baseline, 2 week, 4 week and end of study questionnaires). We have also implemented a bonus structure for the EMA surveys. If participants complete 50% of the EMA surveys (63 surveys), they will receive a further £5. Completing 60%, 70%, 80%, 90% or 100% results in a further £10, £15, £20, £25 or £30 bonus, respectively. Therefore, a participant completing 50% of the EMA surveys and two longer questionnaires would be reimbursed £72 (£63 for the 63 EMA surveys, the £5 bonus for 50% completed EMA surveys and £4 for the two longer questionnaires). A participant completing 100% of the EMA surveys and all four longer questionnaires would be reimbursed the maximum £164 (£126 for the 126 EMA surveys, the £30 bonus for 100% completed EMA surveys and £8 for the two longer questionnaires). ALSPAC participants with the smartwatches will receive an additional £10 reimbursement for taking part in smartwatch component of the study. Reimbursement will be given in the form of Love2Shop vouchers, administered by the respective study teams.

### Measures

A full list of measures that will be assessed throughout the study and their schedules are presented in table 1. A full description of the measures is detailed in the online supplemental material.

**Table 1:** Study measures for the mood in the moment study

Phase	Measure	Source(s) and details	Items (n)	Data collection week						
Pre-study				**0**	**1**	**2**	**3**	**4**	**5**	**6**
	Informed consent		8	x	–	–	–	–	–	–
EMA Questionnaires (baseline, 2 week, 4 week and end of survey only)										
	Menstruation	Does participant currently menstruate	1	x	–	–	–	–	–	–
	Work pattern and caregiving	Adapted from prior shift-work studies; typical vs non-typical work pattern or caring responsibilities	1	x	–	–	–	–	–	–
	Depression	Short Mood and Feelings Questionnaire (SMFQ)^49^	13	x	–	x	–	x	–	x
	Anxiety	Generalised Anxiety Disorder Scale (GAD 7)^50^	7	x	–	x	–	x	–	x
	Resilience	Brief Resilience Scale (BRS)^51^	6	x	–	#	–	#	–	#
	Self-esteem	Rosenberg Self-Esteem Scale (RSES)^52^	10	x	–	#	–	#	–	#
	Cognitive style	Global Cognitive Styles Questionnaire (CSQ)^53^	4	x	–	#	–	#	–	#
	Social support	Social Support Scale^54^	6	x	–	x	–	x	–	x
	Exercise	Adapted from prior studies; frequency of mild, moderate and strenuous exercise	3	x	–	x	–	x	–	x
	Time outdoors	Newly developed; typical daily duration	1	x	–	x	–	x	–	x
	Sleep	Adapted from Pittsburgh Sleep Quality Index (PSQI); sleep quality and impact on daily life^55^	1 to 2*	x	–	x	–	x	–	x
	Pain	Newly developed; pain frequency and impact on life or work	1 to 2*	x	–	x	–	x	–	x
	Recent life events	Adapted from prior life events studies; occurrence of positive and negative life events	1	x	–	x	–	x	–	x
	Hormonal or contraceptive medication	Newly developed; medication type and duration of use	1 to 3*	x	–	x	–	x	–	x
	Gynaecological or reproductive health conditions	Newly developed; history of conditions	1	x	–	–	–	–	–	–
	Mental health conditions	Newly developed; history and type of mental health conditions	1 to 2*	x	–	–	–	–	–	–
	Mental health medication and treatment	Newly developed; recent use of mental health medication or treatment	1	x	–	x	–	x	–	x
	Perceived stress†	Perceived Stress Scale (PSS)^56^	14			x				
	Personality†	Big Five Inventory—Short Form (BFI-SF)^57^	15					x		
	Burnout†	Maslach Burnout Inventory (MBI-GS)^58^	16							x
EMA surveys (asking about last 3 hours, three times a day)										
	Depression‡	Adapted version of Patient Health Questionnaire (PHQ-8/9)^59–61^	8 or 9	–	ooo	ooo	ooo	ooo	ooo	ooo
	Anxiety	Adapted version of Generalised Anxiety Disorder Scale (GAD-2)+1 item on irritability from GAD-7^50^	3	–	ooo	ooo	ooo	ooo	ooo	ooo
	Current location	Where participant has spent most time	1	–	ooo	ooo	ooo	ooo	ooo	ooo
	Current activities	What participant has mostly been doing	1	–	ooo	ooo	ooo	ooo	ooo	ooo
	Diet and food quality	Adapted from prior diet and food insecurity studies; food consumed and type of food	1 to 2*	–	ooo	ooo	ooo	ooo	ooo	ooo
	Physical activity	Highest level of physical activity (from no movement to strenuous exercise)	1	–	ooo	ooo	ooo	ooo	ooo	ooo
	Substance use	Smoking, vaping and alcohol consumption	1	–	ooo	ooo	ooo	ooo	ooo	ooo
	Stress	Feelings of stress	1	–	ooo	ooo	ooo	ooo	ooo	ooo
	Confiding in someone	Feeling able to confide in someone close^62^	1	–	ooo	ooo	ooo	ooo	ooo	ooo
	Social connectedness	Newly developed; enjoyment of social contact online, in-person and via social media	3	–	ooo	ooo	ooo	ooo	ooo	ooo
	Sleep	Adapted from Pittsburgh Sleep Quality Index (PSQI); sleep quality, sleep &andwake time^55^	1 to 3*	–	ooo	ooo	ooo	ooo	ooo	ooo
	Positive and negative affect†	Newly developed: positive and negative feelinsg and reflections	2	-	ooo	ooo	ooo	ooo	ooo	ooo
	Daily menstruation status§	Has period started in past 24 hours	1	–	ooo	ooo	ooo	ooo	ooo	ooo
Passive data collection in ALSPAC only										
	Withings steel HR or ScanWatch Light Smart Watch	Step count, heart rate and sleep		Continuous throughout 6 weeks						

x, administered at baseline or follow-up; #, administered at baseline or follow-up in TEDS only; ooo, administered three times a day across each week.

*Follow-up items dependent on answers provided to initial item.

†Only administered within ALSPAC.

‡PHQ-9 administered one time per day in afternoon survey, PHQ-8 administered in morning and evening survey.

§Only administered in evening survey to participants who indicated they menstruated in baseline questionnaire.

ALSPAC, Avon Longitudinal Study of Parents and Children; EMA, ecological momentary assessment.

### Statistical analysis

Data will be analysed using descriptive and advanced intensive longitudinal data analysis techniques, including descriptive analysis of each item,^22^ mixed effects models^28^ and dynamic structural equation modelling.^42^ Using these techniques will allow us to examine group and participant-level effects and derive individual-level variability of depressive symptoms and their associated risk and protective factors as measured across the EMA period. For example, we can derive indices of depressive dynamics including: the average total or specific depressive symptoms experienced across the study period, the degree of fluctuation in depression from one assessment to the next (symptom variability), the extent to which prior depression states predict subsequent ones and the strength of the association between specific daily experiences (ie, previous sleep, a period of social isolation) and subsequent momentary depressive mood. These indices can then be examined in relation to longer-term depression trajectory membership as previously characterised in each study, enabling investigation of whether individuals with different depression histories show systematically different patterns of short-term depressive dynamics. For smartwatch participants, these within-person dynamics will also be examined alongside passively collected physiological data from wearable devices, including objective sleep metrics, step count and heart rate, to assess the contribution of passively collected data to real-time depressive fluctuations.

### Sample size justification

Our sample size of ~450 participants in TEDS and ~250 participants in ALSPAC is based on previous research comparing long term EMA data collection in case-control designs.^42^ Power calculations for this particular EMA design (recruited from four different trajectories) are not available in current packages. However, simulation data indicated that ~150 participants per trajectory, completing ~50% of their measures would give 80% power to detect associations between depression and modifiable risk factors with small to moderate effect sizes (~0.2–0.3), within each trajectory.

### Ethics and dissemination

Ethical approval to conduct this research in both TEDS and ALSPAC was given favourable opinions by the Edinburgh Medical School Research Ethics Committee (REC References: 25-EMREC-001 and 25-EMREC-030) and the ALSPAC Law and Ethics Committee (Ref: 0027 B4792). The results will be disseminated through journal publications, conferences and seminar presentations and to relevant stakeholders, such as those with a history of depression, policy makers and clinicians.

## Discussion

This protocol outlines the design for a unique EMA study nested within two established longitudinal studies: TEDS and ALSPAC. The purpose of this research is to examine what depression and its correlates look like in day-to-day or moment-to-moment settings in people with different histories of depression trajectories. Building on decades of cohort data that have characterised the long-term epidemiology of depression from childhood into adulthood, our study seeks to extend traditional longitudinal research to examine both long- and short-term depression trajectories. We will examine how depression occurs in close to ‘real time’ and aim to identify proximal, modifiable factors that drive short-term depressive experiences. Our present study is motivated by the need for new evidence to inform interventions that are ecologically valid, timely and responsive to people with different levels of depression risk.

A central strength of this study is the integration of intensive short-term data collection with extensive life-course phenotypic data. Existing EMA studies of depression have largely been conducted in opportunity or clinical samples without access to longitudinal background data,^23^ limiting their ability to contextualise short-term findings within the broader developmental context. In contrast, our study capitalises on the rich existing data of two large, well-characterised cohorts to select participants on the basis of previously established depression trajectory groups. This design enables a level of contextualisation that is not achievable in traditional EMA research, allowing short-term depressive dynamics to be examined against a backdrop of observed genetic and potentially modifiable factors that may have accumulated across the life course. This approach has the potential to yield insights into the real-time epidemiology of depression and ultimately to more time-specific and person-specific interventions and preventions.

A further strength lies in the multimodal nature of the data collection. We are collecting a variety of data encompassing sleep, diet, substance use, exercise, social interactions, daily activities, stress and other domains of mental health. This will allow us to thoroughly examine the epidemiology of depression in people with different depression trajectories for the first time. In addition, the combination of self-reported EMA data with passively collected objective physiological measures such as sleep, activity and heart rate via wearable smartwatch devices provides a more complete picture of the biological and behavioural context in which depressive fluctuations occur than either data source could provide alone.^35 36 43^ Self-report measures of some behaviours such as sleep and physical activity, while valuable, are subject to recall bias and may not accurately reflect objective physiological states.^44^ Passive wearable data can help to validate such self-reported information, and together they can provide large amounts of powerful data that are suitable for advanced analytical methods such as machine learning and depression prediction.^26 45^

The aim of our study is to provide valuable insights into the epidemiology of depression and to inform the potential development of smartphone-based and ecological momentary interventions for depression.^25^ Digital mental health interventions show considerable promise as scalable, accessible tools for improving depression outcomes, but their application is still very much in development. By identifying specific momentary risk and protective factors that strongly predict short-term depressive fluctuations, our study can provide a robust empirical foundation for the development of interventions that are triggered by, and responsive to, real-time signals of elevated risk. Such just-in-time adaptive interventions represent a promising frontier for depression treatment and prevention.^23 25^

Furthermore, we hope our study highlights a unique opportunity to conduct novel study designs that can be embedded within existing studies. Integrating intensive and/or passive smartphone and smartwatch data collection with the vast amount of data available in longitudinal cohort studies could hold significant opportunities to improve our understanding of depression and other diseases and behaviours.

### Limitations

There are several limitations of our study that warrant acknowledgement. First, the EMA component captures depressive experience over a period of 6 weeks, providing a relatively brief window into short-term depressive fluctuations and not a continuous record of daily life over months or years. While such a study design is possible, the burden on participants to complete such measures over months or even years is likely to be too great. As such, a key strength remains the ability to link these short-term depressive symptoms with the longitudinal cohort data. Future studies could look to embed regular periodic assessments of EMA into existing longitudinal studies (ie, once every couple of years) to address this.

Second, as with all EMA studies, our present design is likely to be subject to low compliance, participants rapidly completing the surveys and attrition over the study period. Participants who find the EMA schedule burdensome, or who experience significant worsening of depression during the study, may be less likely to complete all assessments or even consent to join the study to begin with, potentially introducing selection bias, as per some previous research.^46–48^ Although our study protocol has been designed to minimise burden with surveys designed to take no more than a few minutes each, and appropriate incentives and support structures will be in place, some degree of non-random attrition is to be expected. Sensitivity analyses will be used to examine the potential impact of missing data on key findings.

Finally, while our study spans two different cohorts and is designed to maximise sample size within our available resources, statistical power for some of the more complex within-person and between-group analyses may be limited, particularly in the subsample completing smartwatches. Power analyses and simulation studies have been used to inform the study design but replication of key findings in independent samples will ultimately be necessary to establish their robustness.

## Supplementary Material

Reviewer comments

Author's manuscript
