# Supplementary material for: Mood in the moment: a study protocol for embedding ecological momentary assessments into established longitudinal cohorts to examine depression in real time
Source: BMJ Open. 2026 Jun 23;16(6):e122195. doi: 10.1136/bmjopen-2026-122195 (PMC13296039; doi:10.1136/bmjopen-2026-122195)
Supplement: Supplementary data [file bmjopen-16-6-s001.pdf]

Supplementary materials

Mood in the Moment: a study protocol for embedding ecological momentary assessments into established longitudinal cohorts to examine depression in real-time

Additional cohort information - ALSPAC

Pregnant women resident in Avon, UK with expected dates of delivery between 1st April 1991 and 31st December 1992 were invited to take part in the study. 20,248 pregnancies have been identified as being eligible and the initial number of pregnancies enrolled was 14,541. Of the initial pregnancies, there was a total of 14,676 foetuses, resulting in 14,062 live births and 13,988 children who were alive at 1 year of age. When the oldest children were approximately 7 years of age, an attempt was made to bolster the initial sample with eligible cases who had failed to join the study originally. As a result, when considering variables collected from the age of seven onwards (and potentially abstracted from obstetric notes) there are data available for more than the 14,541 pregnancies mentioned above: The number of new pregnancies not in the initial sample (known as Phase I enrolment) that are currently represented in the released data and reflecting enrolment status at the age of 24 is 906, resulting in an additional 913 children being enrolled (456, 262 and 195 recruited during Phases II, III and IV respectively). The phases of enrolment are described in more detail in the cohort profile paper and its update. The total sample size for analyses using any data collected after the age of seven is therefore 15,447 pregnancies, resulting in 15,658 foetuses. Of these 14,901 children were alive at 1 year of age. From the age of 30, there were an additional 101 new participants recruited from 101 pregnancies during Phase V enrolment. The total sample size for analyses using any data collected after the age of 30 is therefore 15,548 pregnancies, resulting in 15,690 foetuses. Of these 15,002 children were alive at 1 year of age. Study data were collected and managed using REDCap (Research Electronic Data Capture) electronic data capture tools hosted at the University of Bristol. REDCap is a secure, web-based software platform designed to support data capture for research studies (Harris et al., 2009).

Reference: Harris, P., et al., *Research electronic data capture (REDCap) – A metadata-driven methodology and workflow process for providing translational research informatics support.* J Biomed Inform, 2009. **42**(2): p. 377-81.

Survey Measures used in Mood and the Moment Study

EMA Schedule and Key Information

Brief Daily Surveys (3 x per day unless stated)

Number of measures per survey: 11 – 13.

Approx time to complete: 3 minutes

The content of the 3 x daily brief daily surveys is almost identical except for order of administration. A summary of each survey (morning, afternoon, evening) is provided below, along with timings of the survey notifications and reminders sent in the m-Path app. A brief description of each survey item and its application in the current EMA study follows, and a detailed description of all survey and questionnaire items including item and response wording and references are provided at the end of the document.

**Morning Survey Order**	**Afternoon Survey Order**	**Evening Survey Order**
Current location and activity	PHQ-9	GAD-2
Diet	Current location and activity	Confiding in others
PHQ-8	Diet	Social connectedness
GAD-2	Exercise	Social media
Sleep	Substance use	Menstruation
Exercise	GAD-2	PHQ-8
Substance use	Stress	Stress
Stress	Positive and negative affect	Positive and negative affect
Positive and negative affect	Social media use	Current location and activity
Confiding in others	Social connectedness	Sleep*
Social connectedness	Confiding in others	Substance use
Social media use	Sleep*	Exercise
		Diet
**Notification time: 8:00am to 9:00am**	**Notification time: 1:00pm to 2:00pm**	**Notification time: 6:00pm to 7:00pm**
**Survey open for 3 hour**	**Survey open for 3 hour**	**Survey open for 3 hour**
**Reminders x 2, every 60 minutes**	**Reminders x 2, every 60 minutes**	**Reminders x 2, every 60 minutes**
	***only asked if previously missed**	***only asked if previously missed**

PHQ-8

- Administered with morning and evening prompts only
- 8 items
- Clinically validated measure
- Adapted version for EMA: replaced ‘Over the last 2 weeks’ with ‘In the last 3 hours’

PHQ 9

- Administered with afternoon prompt only
- 9 items

- Clinically validated measure
- Adapted version for EMA: replaced ‘Over the last 2 weeks’ with ‘In the last 3 hours’

GAD-2 (+ irritability item from GAD-7)

- 3 items
- Clinically validated measure
- Adapted version for EMA: replaced ‘Over the last 2 weeks’ with ‘In the last 3 hours’

Confiding in others

- 1 item
- Validated measure

Positive and negative affect

- 2 items
- Developed by research team at UofE

Stress

- 1 item
- Developed by research team at UofE

Sleep

- 3 items total
- Validated measure: single item on sleep quality from PSQI + 2 items on sleep/awake time
- **Once a day only**

Diet (food quality)

- 2 items
- Developed by research team at UofE

Menstruation

- Administered with evening prompt only
- 1 item
- Developed by research team at UofE

Exercise

- 1 item

- Developed by research team at UofE

Substance use (smoking/vaping & alcohol)

- 1 item
- Developed by research team at UofE

Current location & current activity

- 2 items
- Developed by research team at UofE

Social connectedness

- 2 items
- Developed by research team at UofE

Social media use

- 1 item
- Developed by research team at UofE

Baseline, week-2, -4 and -6 exit Questionnaires

Approx time to complete: 5 minutes

A summary of the questionnaire content is provided below, along with timings of the questionnaire notifications and reminders sent in the m-Path app. A brief description of each questionnaire item and its application in the current study follows, and a detailed description of all survey and questionnaire items including item and response wording and references are provided at the end of the document.

Short mood and feelings questionnaire (SMFQ)

- 13 items
- Clinically validated measure
- Assessed at every sweep

GAD-7

- 7 items
- Clinically validated measure
- Assessed at every sweep

Brief Resilience Scale (BRS)

- 6 items
- Validated measure
- ***Assessed at every sweep in TEDS, baseline questionnaire only in ALSPAC***

Rosenberg Self-Esteem Scale (RSE)

- 10 items
- Validated measure
- ***Assessed at every sweep in TEDS, baseline questionnaire only in ALSPAC***

Global cognitive styles

- 4 items
- Validated measure
- ***Assessed at every sweep in TEDS, baseline questionnaire only in ALSPAC***

Perceived Stress Scale

- 14 items
- Validated measure
- ***2 week follow up questionnaire in ALSPAC only***

Big Five Inventory 15 Item Version

- 15 items
- Validated measure
- ***4 week follow up questionnaire in ALSPAC only***

Maslach Burnout Inventory – MBI-GS

- 16 items
- Validated measure
- ***6 week follow up questionnaire in ALSPAC only***

Hormonal Contraception/Medication Use

- *3 items*
- Developed by research team at UofE
- Assessed at every sweep

Gynaecological or Reproductive Health Conditions

- *1 item*
- Developed by research team at UofE

- ***Baseline questionnaire only***

Pain

- 2 items
- Developed by research team at UofE
- Assessed at every sweep

Working pattern

- 1 item
- Developed by research team at UofE
- Assessed at every sweep

Exercise

- 3 items
- Developed by research team at UofE
- Assessed at every sweep

Time spent outdoors

- 1 item
- Developed by research team at UofE
- Assessed at every sweep

Social Support

- 6 items
- Validated measure
- Assessed at every sweep

Life in the last 2 weeks (exercise, sleep, pain and life events)

- 9 items
- Developed by research team at UofE
- Assessed at every sweep

Antidepressant medication or therapy use in the last 2 weeks

- 1 item
- Developed by research team at UofE
- Assessed at every sweep

Current and previous history of mental health

- 1 item
- Developed by research team at UofE
- ***Baseline questionnaire only***

Exit survey (only at week 6 at the end of the study)

- 1 item
- Developed by research team at UofE

EMA items (Validated Measures from Surveys and Questionnaires)

Adapted Patient Health Questionnaire (PHQ-8 and PHQ-9) (Kroenke et al. 2001)

The PHQ-8 is an 8-item self-administered measure which can be used to screen for depression and assess the severity of depressive symptoms in the previous two weeks. Here we adapt the time frame for the items to ask, ***'In the last 3 hours,*** *I have…' to make it suitable for use as an EMA measure*, and we adapt the sleep question to, 'Last night, I had trouble with sleep' (included in morning survey only). The PHQ-8 is included in the morning and evening surveys.

The PHQ-9, administered in the afternoon survey only, includes an additional item assessing suicidality. Participants answer using a sliding scale with a range between 0 and 100 with the further option for prefer not to say.

Participants will be informed in the participant information sheet and the consent form that we will not be actively monitoring their responses and as such they cannot use the m-Path app and their response to this item to attain help and support. However, if this question is answered positively (i.e., ≥ 1), participants will instantly be directed to help lines and advised to seek support through the application before being able to proceed with the survey.

PHQ-8 and PHQ-9 items [daily surveys]:

In the last 3 hours, I have **had little interest or pleasure in doing things**

0. Not at all

to

100. Constantly

Prefer not to say

In the last 3 hours, I have **felt down, depressed, or hopeless**

0. Not at all

to

100. Constantly

Prefer not to say

Last night, I **had trouble with sleep**

0. Not at all

to

100. Constantly

Prefer not to say

In the last 3 hours, I have **felt tired or have had little energy**

0. Not at all

to

100. Constantly

Prefer not to say

In the last 3 hours, I have **had poor appetite or have been overeating**

0. Not at all

to

100. Constantly

Prefer not to say

In the last 3 hours, I have **felt bad about myself**

0. Not at all

to

100. Constantly

Prefer not to say

In the last 3 hours, I have **had trouble concentrating**

0. Not at all

to

100. Constantly

Prefer not to say

In the last 3 hours, I have **been moving or speaking slowly, or fidgeting more**

0. Not at all

to

100. Constantly

Prefer not to say

In the last 3 hours, I have **had thoughts of hurting myself or that I would be better off dead** *[PHQ-9 question, included in the afternoon survey only. Participant directed to support lines if respond with answers ≥1]*

0. Not at all

to

100. Constantly

Prefer not to say

Reference: Kroenke, K., Spitzer, R. L., & Williams, J. B. W. (2001). The PHQ-9 - Validity of a brief depression severity measure. Journal of General Internal Medicine, 16, 606-613.

Adapted Generalised Anxiety Disorder Scale [Two-item version + irritability item] (GAD-2) (Spitzer et al. 2006)

The GAD-2 uses the first two items of the GAD-7 to assess critical facets of anxiety disorders. This shorter measure has shown to have good sensitivity and specificity to that of the GAD-7 (Plummer et al. 2016). Here we use the GAD-2 along with an additional question on irritability from the GAD-7, to be administered in all 3 brief daily surveys. We adapt the time frame for the items to ask, *'**In the last 3 hours,** have you…' to make it suitable for use as an EMA measure,* as with the PHQ-8/9 above.

Generalised Anxiety Disorder 2 + irritability [daily surveys]

In the last 3 hours, have you been **feeling nervous, anxious or on edge?**

0. Not at all

to

100. Constantly

Prefer not to say

In the last 3 hours, have you been **unable to stop or control worrying?**

0. Not at all

to

100. Constantly

Prefer not to say

In the last 3 hours, have you been **feeling easily annoyed or irritable?**

0. Not at all

to

100. Constantly

Prefer not to say

Generalised Anxiety Disorder Scale [Seven-Item] (GAD-7) (Spitzer et al. 2006)

The GAD-7 is a seven-item measure which screens for anxiety severity in clinical and general populations. Participants will also be administered the full GAD-7 during the baseline questionnaire and at every 2-week follow up questionnaire. The original item framing of '*Over the last 2 weeks...*' will be used for administering the GAD-7 in these bi-weekly questionnaires, but for ease of comprehension we have removed the "been bothered with" phrasing in each question. See reference for full list of questions.

Reference: Spitzer, R. L., Kroenke, K., Williams, J. B., & Löwe, B. (2006). A brief measure for assessing generalized anxiety disorder: the GAD-7. Archives of internal medicine, 166(10), 1092-1097.

Social Support Scale (Shakespeare-Finch & Obst, 2011)

The social support scale is a six-item scale to assess self-reported/perceived social supports. This measure will be administered at baseline and at every two-week follow-up questionnaire. See reference for full list of questions.

Reference: Shakespeare-Finch, J., & Obst, P.L. (2011). The Development of the 2-Way Social Support Scale: A Measure of Giving and Receiving Emotional and Instrumental Support. Journal of Personality Assessment, 93, 483 - 490.

Short Mood and Feelings Questionnaire (SMFQ) (Angold & Costello, 1987)

The SMFQ is a 13-item self-report questionnaire in which participants answer items to assess key depression symptoms. This measure has been used in clinical and general populations. Participants will be administered the SMFQ at baseline and at every two-week follow-up questionnaire. See reference for full list of questions.

Reference: Angold, A., & Costello, E. J. (1987). *Short Mood and Feelings Questionnaire (SMFQ, SMFQ-C, SMFQ-P)* [Database record]. APA PsycTests. https://doi.org/10.1037/t15197-000

Brief Resilience Scale (BRS) (Smith et al. 2008)

The BRS is a brief self-report scale in which participants answer six items to measure their ability to bounce back or recover from stress. This measure will be administered at baseline in ALSPAC and TEDS and at every two-week follow up in TEDS only. See reference for full list of questions.

Reference: Smith, B. W., Dalen, J., Wiggins, K., Tooley, E., Christopher, P., & Bernard, J. (2008). The brief resilience scale: assessing the ability to bounce back. *International journal of behavioral medicine*, *15*(3), 194–200. https://doi.org/10.1080/10705500802222972

Rosenberg Self-Esteem Scale (RSE) (Rosenberg, 1965)

The RSE is a ten-item self-report measure used to assess global self-esteem in adults. This measure will be administered at baseline in ALSPAC and TEDS and at every two-week follow up in TEDS only. See reference for full list of questions.

Reference: Rosenberg, M. (1965). *Rosenberg Self-Esteem Scale (RSES)* [Database record]. APA PsycTests. https://doi.org/10.1037/t01038-000

Global Cognitive Styles Questionnaire (CSQ) (Pearson et al. 2013)

The CSQ is a measure utilised to assess a participant's negative cognitive style through four items. This measure will be administered at baseline in ALSPAC and TEDS and at every two-week follow up in TEDS only. See reference for full list of questions.

Reference: Pearson, R. M., Fernyhough, C., Bentall, R., Evans, J., Heron, J., Joinson, C., Stein, A. L., & Lewis, G. (2013). Association between maternal depressogenic cognitive style during pregnancy and offspring cognitive style 18 years later. The American journal of psychiatry, 170(4), 434–441. https://doi.org/10.1176/appi.ajp.2012.12050673

The Perceived Stress Scale (Cohen et al., 1983)

The PSS is used to measure perceived stress in the last month and will be assessed in ALSPAC at the 2-week questionnaire only. See reference for full list of questions.

Reference: Cohen, S., Kamarck, T., & Mermelstein, R. (1983). Perceived Stress Scale [Database record]. APA PsycTests. https://doi.org/10.1037/t02889-000

Big Five Inventory – 15 Item Short Form (Lang et al. 2011)

The Big Five Inventory - 15 item Short Form is a shorter version of the traditional Big Five Inventory for assessing personality. This will be measured administered in ALSPAC at the 4 week follow up only. See reference for full list of questions.

Reference: Lang, F. R., John, D., Lüdtke, O., Schupp, J., & Wagner, G. G. (2011). Short assessment of the Big Five: robust across survey methods except telephone interviewing. Behavior research methods, 43(2), 548–567. https://doi.org/10.3758/s13428-011-0066-z

Maslach Burnout Inventory: MBI-GS – (Maslach & Jackson, 1981)

The MBI-GS is a short form measure of the original Maslach Burnout Inventory and is a concise measure of burnout. It will be administered in ALSPAC at the 6-week questionnaire only. See reference for full list of questions.

Maslach, C., & Jackson, S. E. (1981). The Measurement of Experienced Burnout. *Journal of Occupational Behaviour*, *2*(2), 99–113. https://doi.org/10.1002/job.4030020205

EMA Items (Non-Validated Measures from Surveys and Questionnaires)

Current Location and Activities

This two-item measure assess where participants have spent the most time between prompts and to report on their main activities during this time. Items assessing location and current activities may give insight into how a participant's environment or activity may be related to their reported mood and other factors reported via daily EMA measures. These items are included in all 3 brief daily surveys.

In the last 3 hours, **where have you spent the most time?**

1. At home

2. At a friend's place
3. At my romantic partner's place
4. At my family's place
5. At work / place of education
6. In a car / on public transport
7. Other indoor location
8. Outdoor location (urban/city)
9. Outdoor location (rural/in nature)
10. At a hotel or Airbnb

In the last 3 hours, **what have you mainly been doing?** Select all that apply:

1. Social activities (in person)
2. Online social activities
3. Physical activity/leisure (e.g., walking, exercising)
4. Mentally stimulating activities (e.g., reading a book, puzzles, board game)
5. Passive leisure (e.g., watching TV, scrolling social media)
6. Studying / working
7. Chores (e.g., groceries, cooking, cleaning)
8. Childcare
9. Travelling / on my way somewhere
10. Eating
11. Sleeping
12. Other

Working patterns

To gather data on non-standard working patterns and other reasons for participants systematically missing surveys and questionnaires, they will be asked the following question:

It's important that you answer as many surveys and questionnaires as possible throughout the study, however we understand that work and other commitments might stop you from doing so. We'd still love you to take part, even if you can't respond to every notification.

Please select one of the options below to let us know about any commitments that will regularly stop you from responding to notifications in the next 6 weeks:

1. I work shifts or have a non-typical working pattern (e.g., work starts before 7am or after 2pm, hours rotate, or regularly includes hours outside of 7am to 6pm)
2. I have caring responsibilities
3. I am unable to access my phone during working hours
4. N/A – I can normally respond to notifications at any time.

Stress

Stress will be assessed using the following single item assessed with a sliding scale, included in all 3 brief daily surveys.

In the last 3 hours, **have you been feeling stressed?**

0. Not at all

to

100. Extremely

Prefer not to say

Confiding in Someone

Participants are asked a single item *'In the last 3 hours, have you felt like you could confide in someone close to you?'*. This question is included in all 3 brief daily surveys.

In the last 3 hours, **have you *felt like* you could confide in someone close to you?**

0. Not at all

1. Some of the time

2. More than half the time

3. Nearly all the time

4. Prefer not to say

Positive and Negative Affect

Positive and negative affect will be assessed using the following two items assessed with a sliding scale for the first one and a selection of options for the second, included in all 3 brief daily surveys in ALSPAC only.

How positive or negative do you feel right now?
0. Positive

to

10. Negative

Prefer not to say

Reflect on what you were thinking about prior to answering this survey
1. I was conscious of my inner feelings.

2. I was self-conscious about the way I look or was concerned about the way I present myself.

3. I was conscious of what is going on around me.

4. I was reflective about my life.

5. I was concerned about what other people think of me

6. I was aware of my innermost thoughts.

7. None of the above

Prefer not to say

Exercise

Participants will be asked a variety of items on exercise across the surveys and questionnaires. The questions for the daily surveys, were adapted from a question in Hirvensalo et al. (2000) and response options from Bruening et al. (2016).

Additionally, at baseline and for every two-week follow-up, participants will be asked three further items on exercise and time spent outdoors.

[Following Question is asked in brief daily surveys]

In the last 3 hours, what has been your highest level of physical activity?

0. No movement (e.g., sitting at a desk, on the sofa, travelling by car or public transport)
1. Moved only in connection with necessary tasks (e.g., household chores)
2. Gentle exercise or leisurely activities requiring little effort (e.g., gentle walking, gardening, yoga)
3. Moderate exercise - increased your heart rate but not exhausting e.g., brisk walking, dancing, cycling, weights)
4. Strenuous exercise causing sweating / heavy breathing (e.g., running, football, fast swimming, HIIT)

[The following questions are asked in bi-weekly questionnaires]

In the past 2 weeks, how many times have you done any strenuous exercise to the point of sweating and heavy breathing (e.g., running, HIIT, football, fast swimming)?

1. None
2. 1 – 2 times
3. 3 – 4 times
4. 5 – 7 times
5. 8 – 10 times
6. 11 - 13 times
7. 14 or more times

In the past 2 weeks, how many times have you done any moderate exercise that increased your heart rate but was not exhausting (e.g., walking fast, hiking, lifting weights, dancing)?

1) None
2) 1 – 2 times
3) 3 – 4 times
4) 5 – 7 times
5) 8 – 10 times
6) 11 - 13 times
7) 14 or more times

In the past 2 weeks, how many times have you done any mild exercise that required little effort (e.g., gentle walking, yoga, bowling)

1) None
2) 1 – 2 times
3) 3 – 4 times
4) 5 – 7 times
5) 8 – 10 times
6) 11- 13 times
7) 14 or more times

Thinking about the past 2 weeks, how many minutes did you spend outdoors on a typical day?

1) 0-15 minutes
2) 16-60 minutes
3) 61-120 minutes (1-2 hours)
4) 121-180 minutes (2-3 hours)
5) 181+ minutes (3+ hours)

Sleep

Participants will be asked to rate the quality of their sleep using items from the Pittsburgh Sleep Quality Index (PSQI) (Buysse et al. 1989). The sleep quality item will be included in all 3 daily surveys to reduce possible data loss in participants with atypical sleep patterns (e.g., shift-workers). However, the sleep and wake time questions will only be asked once per day.

Further, during baseline and bi-weekly questionnaires participants will be asked again to rate the overall quality of their sleep. If a participant indicates that their average sleep has been of bad quality (answers ≥4) they will be prompted to answer a further question about how their sleep affects their daily life.

[Following Question is asked in brief daily surveys]

How would you rate the quality of your sleep last night?

0. Very poor
1. Poor
2. Neutral
3. Good
4. Very good

What time did you fall asleep last night ________ [answer in hours/minutes]

What time did you wake up today ____________ [answer in hours/minutes]

[Following Questions are asked in bi-weekly questionnaires]

In the past 2 weeks, how would you rate the quality of your sleep on average?

1. Very good
2. Fairly good
3. Average
4. Fairly poor
5. Very poor

In the past 2 weeks, have your sleep difficulties interfered with your daily life? [only for participants who answer 4 or 5 to preceding question]

1. Not at all

2. A little bit
3. Moderately
4. Quite a bit
5. Extremely

Diet

To assess diet and diet quality in participants we will administer two items. The first will ask participants if they have eaten since the last prompt. Those who indicate that they have eaten, they will be directed to the second item asking about the type of food they have eaten (e.g., processed or homemade food). These items are adapted from Berge et al. (2023) and will be included in all 3 daily surveys.

Have you eaten in the last 3 hours? Select all that apply:

1. Yes, I have eaten a main meal (breakfast, lunch, or dinner)
2. Yes, I have eaten a snack
3. No, I have not eaten since the last prompt

Which best describes the type of food you ate on this occasion? Select all that apply: **[only for participants who answer 1 or 2 to preceding question]**

1. Fast food / take-out (consumed at fast food restaurant, home, or other location)
2. Other food eaten outside the home (e.g., a restaurant, café)
3. Pre-prepared or processed foods (e.g., frozen, oven-cook or microwaveable meals, breakfast cereals, crisps, biscuits, etc.)
4. Homemade / freshly prepared food (e.g., made from fresh fruits or vegetables, meat, fish, dairy, grains, pulses)

Menstruation

A single item will enquire as to whether participants have menstruated in the last 24 hours. There will be a '*prefer not to say*' option for those who do not wish to answer.

Did you have your period **in the last 24 hours?**

1. Yes
2. No

Hormonal Contraception/Medication Use and Gynaecological or Reproductive Health Conditions

Three questions will enquire as to whether participants are currently taking any hormonal contraceptives or medications at baseline and every 2-weekly questionnaire. A single question about experience of gynaecological or reproductive health conditions will be included in the baseline questionnaire.

Are you currently using any hormonal contraceptives or medications? This can be for any reason (e.g., birth control, acne, pain/cramps)

1. Yes
2. N/A
3. I don't currently take any hormonal contraception or medication
4. I’ve stopped taking hormonal contraceptives or medications within the last three months.
5. I use a non-hormonal copper coil/IUD

Please select the type of hormonal contraceptives or medications you are currently taking (select all that apply)

[Only for participants who answer ‘Yes’ to previous question]

1. Combined pill (e.g., Microgynon, Rigevidon, Ovranette)
2. Progestogen only pill (“mini-pill” e.g., Cerazette, Cerelle, Norgeston)
3. Contraceptive injection (e.g., Depo-Provera, Sayana Press)
4. Contraceptive patches (e.g., Evra Patches)
5. Vaginal ring (e.g., NuvaRing)
6. Hormonal coil/IUS (Mirena, Levosert, Benilexa, Kyleena, Jaydess)
7. Contraceptive implant (e.g., Nexplanon)
8. Other (please give details): [Prompts free text box]

How long have you been taking any type of hormonal contraceptive for?

[Only for participants who answer ‘Yes’ to taking hormonal contraception/medication]

1. 0 – 3 months
2. 3 – 6 months
3. 6 – 12 months
4. 1 year – 2 years
5. 2 years plus

Have you ever had any of the following gynaecological or reproductive health conditions? Select all that apply:

[baseline questionnaire only]

1. N/A
2. No
3. Endometriosis
4. Polycystic ovary syndrome (PCOS)
5. Fibroid(s)
6. Ovarian cyst
7. Pelvic inflammatory disease (PID)
8. Other (please give details) [Prompts free text box]

Substance Use (Smoking and Alcohol)

We will assess substance use with a single item in all 3 brief daily surveys.

In the last 3 hours, have you done any of the following? Select all that apply:

1. Smoked
2. Vaped
3. Drank alcohol
4. None of the above

Social Connectedness

Social connectedness will be assessed with three items asking participants how enjoyable they have found different types of social contact, including in-person and online. Items are adapted from Fried et al. (2023). These will be administered in all 3 brief daily surveys.

In the last 3 hours, **how enjoyable was spending time with other people in person?**

1. Not at all
2. Somewhat
3. Moderately
4. Very
5. Extremely
6. I haven’t had any in-person contact in the last 3 hours

In the last 3 hours, **how enjoyable was spending time online using social media sites or apps such as Instagram, TikTok or X?**

1. Not at all
2. Somewhat

3. Moderately
4. Very
5. Extremely
6. I haven’t spent any time using social media in the last 3 hours

In the last 3 hours, **how enjoyable was spending time with other people online through messaging sites (e.g., WhatsApp), video calls, or by phone?**

1. Not at all
2. Somewhat
3. Moderately
4. Very
5. Extremely
6. I haven’t had any online social interactions in the last 3 hours

Pain

Participants will be asked about their experience of pain in the baseline questionnaire and at every two-week follow-up questionnaire. Those who report experiencing any pain will be asked a follow up question about the impact this has had on their life or work activities.

In the past 2 weeks, how often have you had any pain?

1. Never
2. Some days
3. Most days
4. Every day
5. Prefer not to say

In the past 2 weeks, how often did pain limit your life or work activities? [only for participants who answer 2 -4 to preceding question]

1. Never
2. Some days
3. Most days
4. Every day
5. Prefer not to say

Recent Life Events

At baseline and at every two-week follow-up questionnaire, participants will be asked if they have experienced a range of recent life events. By assessing life events over the previous two weeks the questionnaire shall elucidate potential positive or negative circumstances which may impact depressive symptoms and mood.

In the past 2 weeks, have any of the following happened to you [tick all that apply]?

1. You entered a new romantic relationship
2. You had a mild illness (e.g., cough, cold, flu, fever)
3. You became seriously ill
4. You accomplished a personal achievement
5. You got into serious financial problems
6. You or your partner became pregnant
7. You started a new job
8. A romantic relationship ended
9. You took a holiday
10. A friend or family member died
11. You or your partner had a baby
12. You had a big argument with a friend or family member
13. You lost your job
14. No significant life events in the past 2 weeks
15. Other (please give details):
16. Prefer not to say

Treatment and Medication

Participants will be asked if they have received any of the following treatments for anxiety or depression: antidepressant medication, cognitive behavioural therapy, any other form of psychological therapy, or any self-help guidance. Participants will be asked to provide this information at baseline and at every two-week questionnaire following this.

In the past 2 weeks, have you taken or received any of the following? Select all that apply:

1. Antidepressant medication for anxiety or depression
2. Cognitive behavioural therapy for anxiety or depression
3. Other forms of psychological therapy for anxiety or depression
4. Any self-help guidance for anxiety or depression
5. None of the above
6. Prefer not to say

Current and Previous Mental Health

Participants will be asked about current and previous mental health diagnoses. Participants will be asked about this at baseline only.

Have you ever been diagnosed with a mental health condition by a health-care professional (e.g., doctor, psychiatrist, therapist, nurse, healthcare professional with specialist training)?

1. Yes, I have a current mental health diagnosis.
2. Yes, I have had a past mental health diagnosis.
3. Yes, I have **both a previous and current** mental health diagnosis.
4. No, I have never received a mental health diagnosis.
5. I'm not sure.
6. Prefer not to say

Please select which mental health diagnosis [select all that apply]

[Only for participants who answer 'Yes' to even been diagnosed with a mental health condition]

1. Depression

2. Anxiety

3. Phobia

4. Bi-polar disorder

4. Body dysmorphia

5. Premenstrual dysphoric disorder

6. Post Traumatic Stress Disorder

7. Obsessive Compulsive Disorder

8. An eating disorder

9. Psychosis

10. Schizophrenia or schizoaffective disorder

11. A personality disorder

12. Other (not listed)

Questionnaire and Study Feedback

Following the completion of the EMA study at six weeks participants will be asked to provide feedback on their experience of using the m-Path application and of participation in the study in general.

Helplines

The helplines below will be shown at the end of every daily survey and at the end of every questionnaire. Additionally, if someone answers the PHQ-9 with a positive score of ≥1 they will immediately be shown the helplines below before being able to progress with the survey.

- **SAMARITANS -** Emotional support for everyone
  Website: https://www.samaritans.org/scotland/ | *Tel:* 116 123 (24 hours)
- **MIND -** Advice and support for anyone with a mental health problem
  Website: https://www.mind.org.uk | *Tel:* 0300 123 3393 Text: 86463
- **SHOUT** - Support around self-harm
  Website: https://giveusashout.org/get-help/issues/help-self-harm/ | *Text:* 85258
- **NHS 111 -** If you need medical help right now, 111 online can tell you what to do next | *Website:* https://111.nhs.uk.
