# Supplementary material for: Mood in the moment: a study protocol for embedding ecological momentary assessments into established longitudinal cohorts to examine depression in real time
Source: BMJ Open. 2026 Jun 23;16(6):e122195. doi: 10.1136/bmjopen-2026-122195 (PMC13296039; doi:10.1136/bmjopen-2026-122195)
Supplement: Reviewer comments [file bmjopen-2026-122195.reviewer_comments.pdf]

PEER REVIEW HISTORY

BMJ Open publishes all reviews undertaken for accepted manuscripts. Reviewers are asked to complete a checklist review form and are provided with free text boxes to elaborate on their assessment. These free text comments are reproduced below.

ARTICLE DETAILS

Title (Provisional)

Mood in the Moment: a study protocol for embedding ecological momentary assessments into established longitudinal cohorts to examine depression in real-time

Authors

Kwong, Alex S F; Moody, Sarah; Taylor, Adele M; Lockhart, Celestine; Ogden, Rachel; Leal, Ray; Mcmillan, Andrew; Harvey, Jessica; Brierley, Elizabeth; Simmons, Anne; Matthews, Sarah; Hobbs, Richard; Eley, Thalia C; Jacobson, Nicholas C.; Murray, Aja

---

This article was not externally reviewed at BMJ Open. Protocols that have been independently assessed prior to submission to BMJ Open are usually fast-tracked to publication on the grounds that further substantial changes will not be possible. This independent assessment will usually be external, independent review for both a substantial grant award from a non-commercial or government funder and ethics approval.
