# Supplementary material for: Mood in the moment: a study protocol for embedding ecological momentary assessments into established longitudinal cohorts to examine depression in real time
Source: BMJ Open. 2026 Jun 23;16(6):e122195. doi: 10.1136/bmjopen-2026-122195 (PMC13296039; doi:10.1136/bmjopen-2026-122195)
Supplement: Author's manuscript [file bmjopen-2026-122195.draft_revisions.pdf]

BMJ Open is committed to open peer review. As part of this commitment we make the peer review history of every article we publish publicly available.

When an article is published we post the peer reviewers' comments and the authors' responses online. We also post the versions of the paper that were used during peer review. These are the versions that the peer review comments apply to.

The versions of the paper that follow are the versions that were submitted during the peer review process. They are not the versions of record or the final published versions. They should not be cited or distributed as the published version of this manuscript.

BMJ Open is an open access journal and the full, final, typeset and author-corrected version of record of the manuscript is available on our site with no access controls, subscription charges or pay-per-view fees (http://bmjopen.bmj.com).

If you have any questions on BMJ Open's open peer review process please email info.bmjopen@bmj.com

BMJ Open

Mood in the Moment: a study protocol for embedding ecological momentary assessments into established longitudinal cohorts to examine depression in real-time

Journal:	*BMJ Open*
Manuscript ID	bmjopen-2026-122195
Article Type:	Protocol
Date Submitted by the Author:	05-May-2026

Complete List of Authors:	Kwong, Alex S F; University of Edinburgh, Division of Psychiatry; University of Bristol, Population Health Sciences<br>Moody, Sarah ; University of Edinburgh, Division of Psychiatry<br>Taylor, Adele; The University of Edinburgh, Psychology<br>Lockhart, Celestine ; King's College London, Social, Genetic and Developmental Psychiatry<br>Ogden, Rachel ; King's College London, Social, Genetic and Developmental Psychiatry<br>Leal, Ray; King's College London, Department of Psychological Medicine; King's College London, Department of Psychological Medicine<br>Mcmillan, Andrew ; King's College London, Social, Genetic and Developmental Psychiatry<br>Harvey, Jessica ; University of Bristol, Population Health Sciences<br>Brierley, Elizabeth ; University of Bristol, Population Health Sciences<br>Simmons, Anne ; University of Bristol, Population Health Sciences<br>Matthews, Sarah ; University of Bristol, Population Health Sciences<br>Hobbs, Richard ; University of Bristol, Population Health Sciences<br>Eley, Thalia; King's College London, MRC Social, Genetic, and Developmental Psychiatry Centre<br>Jacobson, Nicholas ; Dartmouth College<br>Murray, Aja; The University of Edinburgh, Department of Psychology

Keywords:	Depression & mood disorders < PSYCHIATRY, Longitudinal studies, Child & adolescent psychiatry < PSYCHIATRY, Adult psychiatry < PSYCHIATRY, STATISTICS & RESEARCH METHODS

SCHOLARONE MANUSCRIPTS

Mood in the Moment: a study protocol for embedding ecological momentary assessments into established longitudinal cohorts to examine depression in real-time

Alex S. F. Kwong[1,2,3*], Sarah Moody[1^], Adele Taylor[1^], Celestine Lockhart[4], Rachel Ogden[4], Ray Leal[4], Andrew McMillan[4], Jessica Harvey[2], Elizabeth Brierley[2], Sarah Matthews[2], Richard Hobbs[2], Anne Simmons[2], Thalia C. Eley[4,5], Nicholas C. Jacobson[6], Aja L. Murray[7]

[1]Division of Psychiatry, Centre for Clinical Brain Sciences, University of Edinburgh, UK

[2]Population Health Sciences, Bristol Medical School, University of Bristol, UK

[3]MRC Integrative Epidemiology Unit, University of Bristol, UK

[4]Social, Genetic and Developmental Psychiatry, King's College London

[5]National Institute for Health and Care Research (NIHR) Maudsley Biomedical Research Centre, South London and Maudsley NHS Foundation Trust, London, UK

[6]Center for Technology and Behavioral Health, Geisel School of Medicine, Dartmouth College

[7]Department of Psychology, University of Edinburgh, Edinburgh, UK

*Corresponding author: Division of Psychiatry, University of Edinburgh, 49 Little France Crescent, BioQuarter, Edinburgh, EH16 4TJ; akwong@ed.ac.uk

^joint second authors

Word Count: 3760 (excluding abstract and references)

For peer review only

Mood in the moment: an EMA study on depression

Abstract

Introduction

Depression is a major global health challenge, with onset commonly occurring in young people. There is an urgent need to better understand the epidemiology of depression to facilitate better interventions and preventions that are person and timing specific. Long-term depression trajectories and Ecological Momentary Assessment (EMA) can help address this need. Embedding EMA designs within established longitudinal cohorts offers a uniquely powerful approach to examine depression in both long-and-short term settings and to identify distal and proximal modifiable risk factors for depression.

Methods and analyses

Our study will include approximately 450 participants from the Twins Early Development Study (TEDS) and 250 participants from the Avon Longitudinal Study of Parents and Children (ALSPAC) who have depression trajectories data previously collected between late childhood and early adulthood. Participants will be recruited from four different depression trajectories in each cohort, based upon participants prior symptoms. They will then undertake EMA surveys of depression, sleep, physical activity, substance use, diet, recent activities and social interactions, three times a day for six weeks, with more detailed questionnaires at baseline, 2, 4 and 6 weeks. We will use descriptive analysis, mixed effects models and dynamic structural equation modelling to examine how depression occurs over this time, how potentially modifiable factors affect depression and vice versa and how these effects vary by different life-course trajectories. This study is designed to ultimately help identify people at heightened risk of depression and/or identify modifiable targets that could inform more effective prevention and intervention strategies.

Ethics and dissemination

Favourable ethical opinions were given by the Edinburgh Medical School Research Ethics Committee (REC References: 25-EMREC-001 and 25-EMREC-030) and the ALSPAC Law and Ethics Committee (Ref: 0027 B4792). The results will be disseminated through journal publications, conferences and seminar presentations and to relevant stakeholders, such as those with a history of depression, policy makers and clinicians.

For peer review only

Strengths and limitations of the study

- This study will use ecological momentary assessment (EMA), embedded within two established longitudinal cohort studies to examine both long- and short-term trajectories of depression and its correlates in around 700 participants.
- A range of measures will be used during the EMA study, allowing for the identification of important modifiable factors related to depression in real-time, for specific groups of individuals with different depression histories.
- In ALSPAC, a subset of participants will also wear smartwatches to passively collect data on sleep, activity and heart rate.
- Attrition is likely to play a role in the EMA data collection, especially for participants with a history of depression.

For peer review only

Introduction

Depression is a global health challenge, affecting over an estimated 330 million people worldwide [1], and predicted to be the leading cause of disability by 2030 [2]. Young people are at particular risk of depression [3], with emerging evidence that depression is worsening within this population [4, 5]. Depression during this period is associated with wide-ranging and long-lasting economic, social, and physiological consequences [6-9]. Evidence suggests that 50% of people who have ever had depression, had an onset before the age of 25 [10], and almost 50% of those who develop depression between 18 and 40 will have a subsequent episode [11]. This makes it crucial to prevent or limit depression during earlier life to mitigate later effects. Understanding what modifiable factors and mechanisms influence the course of depression is therefore of considerable clinical, economic and societal importance. Such research could ultimately help identify people at heightened risk of worsening depression and/or identify modifiable targets that could inform more effective prevention and intervention strategies.

Longitudinal studies that track the same people over time are critical to addressing this line of research. Such studies can estimate depression trajectories across development and highlight how depression develops, persists, and remits across the life course, as well as what trajectories are specific for different aetiologies and outcomes (for reviews see [12-14]). Importantly, they have also identified key risk and protective factors such as sex and pubertal timing [15, 16], genetic vulnerability [17, 18], early adversity [7] and the family environment [19, 20] that likely shape depression over time. Whilst such research is important, these studies typically assess depression at yearly intervals or longer. As such, there is a current paucity in what depression looks like in between these assessments, and how depression and its correlates may fluctuate and drive one another in the short-term, from day-to-day, or even moment to moment settings. A greater understanding of more granular short-term manifestation could inform treatment provision and illuminate the perpetuating or proximal; mechanisms that drive depression in day-to-day life.

Ecological momentary assessment (EMA) has emerged as a powerful tool for addressing this limitation [21-23]. EMA designs can collect data multiple times a day whilst participants go about

For peer review only

their daily routines, enabling researchers to examine depression as it unfolds in naturalistic settings and to identify how specific contextual factors can shape mood and vice versa, all in close to real time [24, 25]. EMA designs can be paired with passive data collection (i.e., through smartwatches) to provide objective measures of factors such as sleep, exercise or heart rate, and their relation to daily mood [26] in a continuous, non-invasive manner. EMA is particularly well suited to capturing the dynamic, person and context-dependent nature of depression, providing the opportunity to measure short-term depression trajectories over a given period. It can also capture symptom variability, the occurrence of correlated factors and perhaps most importantly, reactivity to daily stressors and life events in a way that retrospective designs cannot reliably detect. A growing body of EMA research has demonstrated that short-term fluctuations in depression are both clinically meaningful and informative [27]. For example, EMA designs have shown that depression is not stable, even across a single day [28]. Furthermore, momentary factors such as disrupted sleep [28, 29], reduced physical activity [28, 30, 31] and worse social interactions are believed to influence depressive states in the short term [24, 32, 33]. However, existing EMA studies of depression have predominantly been conducted in small samples (often clinical populations or student cohorts), without access to broader phenotypic or prospectively ascertained life-course data. This limits their capacity to examine how short-term depressive dynamics fit within the broader context of development and the life-course.

To date, long-term trajectory research and short-term intensive data collection have not been conducted in depression research, with only a handful of studies embedding broader EMA designs into longitudinal studies, such as in ADHD [34]. This represents a significant missed opportunity to combine detailed data on depression in the moment and short-term depression trajectories, alongside detailed longer-term depression trajectories. Short-term depression symptoms and the modifiable factors that drive them such as sleep, exercise, diet, substance use, and social interactions could illuminate proximal mechanisms that underpin longer-term depression trajectories - thus highlighting opportunities for more tailored and targeted interventions and preventions that are timing and person specific. Embedding EMA within established longitudinal cohorts, whose participants have well-characterised life-course depression trajectories, offers a uniquely powerful approach to address this

For peer review only

gap. By selecting participants based upon previously characterised depression trajectories, it is possible to examine the real-time epidemiology of depression and how it varies across individuals with different histories of depressive experience. This can also be combined with data collected across development such as psycho-social factors, family history and genetic risk, and identify the momentary risk and protective factors that are most relevant within different trajectories.

In this study, we will use an EMA design embedded within two established longitudinal studies: the Twins Early Development Study (TEDS) and the Avon Longitudinal Study of Parents and Children (ALSPAC) to study depression in both the long- and short-term. Both studies have extensive life-course data on depression and its determinants, allowing us to derive clinically meaningful and robust depression trajectories from childhood to early/emerging adulthood. The nature of this study will allow us to examine how short-term depressive dynamics occur and vary within the context of longer-term depression trajectories, and how momentary risk and protective factors such as sleep, physical activity, diet, substance use, social interactions and stress contribute to these fluctuations in real time. Furthermore, in a subsample of ALSPAC participants, concurrent passive data collection via wearable smartwatch devices will be collected to enrich this picture by providing objective physiological measures of sleep, physical activity, and heart rate, complementing and contextualising the self-reported EMA data [35, 36]. Our core research questions are:

- RQ1) How does depression occur and vary in the short-term — from day-to-day or even moment-to-moment settings in people with different prior life-course depression trajectories?
- RQ2) Which momentary risk and protective factors such as sleep, stress, physical activity, diet, substance use, and social interactions are most strongly associated with short-term fluctuations and symptoms in depression in daily life, and how do these vary by different depression trajectories?
- RQ3) How do short-term depression dynamics relate to longer-term depression trajectories previously characterised in the cohort, and do they mediate or moderate the associations between established risk factors and longer-term outcomes?

For peer review only

Exploratory analyses may be used to address a range of additional questions, including examination of individual differences in the links between momentary risk factors and depression, using machine learning to predict greater levels of depression and to identify further potential targets for personalised and timely intervention. For more ambitious questions like RQ3, we will pre-register analysis on the open science framework.

For peer review only

Methods and analysis

Existing study participants

Participants will be drawn from two existing longitudinal cohort studies: The Twins Early Development Study (TEDS) and the Avon Longitudinal Study of Parents and Children. In TEDS, the original sample comprised of 13,759 families with newborn twins, who participated in the first assessment wave at 18 months. Families were recruited from the Office of National Statistics' national register of twins born in England and Wales between 1994 and 1996 [37]. In ALSPAC, pregnant women resident in Avon, UK with expected delivery between 1st April 1991 and 31st December 1992 were invited to take part in the study. A total of 14,901 children alive at 1 year of age participated in ALSPAC [38-40]. Further cohort information is provided in the supplementary materials. Overall ethical approval for TEDS was granted by the King's College London Research Ethics Committee (References: PNM/09/10–104, HR/DP-20/2122060, and RESCM-24/25-22060), and consent was given by participants at each wave. In ALSPAC, overall ethical approval for the study was obtained from the ALSPAC Ethics and Law Committee and the Local Research Ethics Committees. Informed consent for the use of existing data collected was obtained from participants following the recommendations of the ALSPAC Ethics and Law Committee at the time. Participants can contact the study team at any time to retrospectively withdraw consent for their data to be used. Study participation is voluntary and during all data collection sweeps, information was provided on the intended use of data. In TEDS, the study website contains details of all data that is available: https://datadictionary.teds.ac.uk/home.htm. In ALSPAC, the study website contains details of all data that is available through a fully searchable data dictionary and variable search tool and reference the following webpage: http://www.bristol.ac.uk/alspac/researchers/our-data/.

EMA participants

The present study will include approximately 450 participants from TEDS and 250 participants from ALSPAC who have depression trajectories data previously collected between late childhood and early adulthood. We will only recruit participants who have responded to the most recent questionnaire on

For peer review only - http://bmjopen.bmj.com/site/about/guidelines.xhtml

depression within each study (both assessed in 2025) to ensure that participant belong to the most up to date depression trajectories. Participants in the EMA study will have a mean age of 29.5 years in TEDS and 32.6 in ALSPAC. All participants will be required to have completed a minimum of two depression assessments (one in childhood/adolescence and another in late adolescence/early adulthood) to be included in the initial trajectory analysis. Based upon recent questionnaire data collected in both cohorts, we have a maximum sample of 3275 participants in TEDS and 3866 participants in ALSPAC from which to recruit participants into the EMA section of the study. Based upon previous research [7, 41], we estimated four similar trajectories per cohort (a stable low trajectory, an adolescent-onset trajectory, an adolescent-limited trajectory and a persistent trajectory), see Figure 1. We will recruit equally from each trajectory group to ensure a matched case-control analysis, randomising the order in which we invite participants to join the EMA study. In ALSPAC, ~100 participants will be given Withings smartwatches to passively collect data on sleep, activity and heart rate throughout the study. Figure 2 shows a graphical representation of our study.

Inclusion criteria

The inclusion criteria for this study require participants to be active and consenting participants of the TEDS and ALSPAC studies and have previously measured data on depression (i.e., a minimum of two depression assessments [one in childhood/adolescence and another in late adolescence/early adulthood]). Participants will be required to have a sufficient understanding of written and spoken English language to understand study information and instructions, provide informed consent, and participate in the text-based EMA surveys to be completed via smartphone application. Participants will also be required to own a smartphone device with access to either the app store (Apple iPhones) or Google Play Store (Android phones) to download the smartphone application m-Path to allow participation in the study for a period of six consecutive weeks. In ALSPAC, participants must be willing and able to download the Withings smartwatch application and wear a Withings smartwatch for a period of six weeks (if consented to this aspect of the study). Across both studies, participants must be available to participate in the study for a consecutive six-week period without significant breaks while the data collection is live.

For peer review only

Patient and public involvement

Individuals with and without depression have contributed to the design of this study. We first consulted people with lived experience of depression as part of an ongoing user-led citizen science project called 'The Depression Detectives'. In their capacity as advisors, they saw value in identifying patterns of depression early through smartphones and to identify key factors that could prevent depression from getting worse and leading to long-term problems. Next, a group of adolescents recruited as part of a patient and public lived experience group determined feasibility of the study, recommending the types of factors to include and duration of the study. Finally, active participants from the ALSPAC study provided guidance on the duration of the study, measures used and amount of reimbursement that would be suitable for the study.

Data collection

Data collection began in September 2025 and is expected to complete by August 2026. Analysis will be staggered across this period to avoid seasonality effects. Figure 2 shows a graphical representation of our study where participants will be recruited based upon their existing depression trajectories. Participants will then be invited to join this EMA study and provide consent.

Participants who give consent and begin the study will be sent instructions on how to download the 'M-Path' EMA smartphone application. In ALSPAC, ~100 random (equal across trajectories) participants will be recruited into the smartwatch element of the study will also be sent instructions on how to download the 'Withings' smartphone application. Each participant will be sent their own unique and pseudo-anonymised login that only the respective study teams can use to identify participants.

Participants who successfully join the study will first complete a baseline questionnaire and have seven days to complete this before they are removed from the study. Completion of this baseline questionnaire will initiate EMA surveys starting the next morning. EMA surveys will occur 3 times a day (randomly in the morning [8am to 9am], afternoon [1pm to 2pm] and evening [6pm-7pm]) for six weeks (for a maximum of 126 surveys), all through the 'M-Path" smartphone application. These

For peer review only

EMA surveys are designed to be completed in under 3 minutes and each daily survey is available for 3 hours from the initial prompt. Reminders were sent after 60 and 120 minutes. Slightly longer questionnaires (taking approximately 5 minutes to complete) are also available to complete at the two- and four-week intervals. A final end-of-study questionnaire is then available to complete at the end of the study at week six. Participants will be reimbursed £1 for every EMA survey they complete, and £2 for each questionnaire they complete (baseline, two-week, four-week and end of study questionnaires). We have also implemented a bonus structure for the EMA surveys. If participants complete 50% of the EMA surveys (63 surveys), they will receive a further £5. Completing 60%, 70%, 80%, 90% or 100% results in a further £10, £15, £20, £25 or £30 bonus, respectively. Therefore, a participant completing 50% of the EMA surveys and 2 longer questionnaires would be reimbursed £72 (£63 for the 63 EMA surveys, the £5 bonus for 50% completed EMA surveys and £4 for the two longer questionnaires). A participant completing 100% of the EMA surveys and all four longer questionnaires would be reimbursed the maximum £164 (£126 for the 126 EMA surveys, the £30 bonus for 100% completed EMA surveys and £8 for the two longer questionnaires). ALSPAC participants with the smartwatches will receive an additional £10 reimbursement for taking part in smartwatch component of the study. Reimbursement will be given in the form of Love2Shop vouchers, administered by the respective study teams.

Measures

A full list of measures that will be assessed throughout the study and their schedules are presented in Table 1. A full description of the measures is detailed in the online supplementary material.

Statistical analysis

Data will be analysed using descriptive and advanced intensive longitudinal data analysis techniques, including descriptive analysis of each item [22], mixed effects models [28], and dynamic structural equation modelling (DSEM) [42]. Using these techniques will allow us to examine group and participant-level effects and derive individual-level variability of depressive symptoms and their associated risk and protective factors as measured across the EMA period. For example, we can derive

For peer review only

indices of depressive dynamics including: the average total or specific depressive symptoms experienced across the study period, the degree of fluctuation in depression from one assessment to the next (symptom variability), the extent to which prior depression states predict subsequent ones, and the strength of the association between specific daily experiences (i.e., previous sleep, a period of social isolation) and subsequent momentary depressive mood. These indices can then be examined in relation to longer-term depression trajectory membership as previously characterised in each study, enabling investigation of whether individuals with different depression histories show systematically different patterns of short-term depressive dynamics. For smartwatch participants, these within-person dynamics will also be examined alongside passively collected physiological data from wearable devices, including objective sleep metrics, step count, and heart rate, to assess the contribution of passively collected data to real-time depressive fluctuations.

Sample size justification

Our sample size of approximately 450 participants in TEDS and approximately 250 participants in ALSPAC is based upon previous research comparing long term EMA data collection in case-control designs [42]. Power calculations for this particular EMA design (recruited from four different trajectories) are not available in current packages. However, simulation data indicated that ~150 participants per trajectory, completing ~50% of their measures would give 80% power to detect associations between depression and modifiable risk factors with small to moderate effect sizes (~0.2-0.3), within each trajectory.

Ethics and dissemination

Ethical approval to conduct this research in both TEDS and ALSPAC was given favourable opinions by the Edinburgh Medical School Research Ethics Committee (REC References: 25-EMREC-001 and 25-EMREC-030) and the ALSPAC Law and Ethics Committee (Ref: 0027 B4792). The results will be disseminated through journal publications, conferences and seminar presentations and to relevant stakeholders, such as those with a history of depression, policy maker and clinicians.

For peer review only

Discussion

This protocol outlines the design for a unique EMA study nested within two established longitudinal studies: TEDS and ALSPAC. The purpose of this research is to examine what depression, and its correlates look like in day-to-day, moment-to-moment settings in people with different histories of depression trajectories. Building on decades of cohort data that have characterised the long-term epidemiology of depression from childhood into adulthood, our study seeks to extend traditional longitudinal research to examine both long- and short-term depression trajectories. We will examine how depression occurs in close to 'real time', and aim to identify proximal, modifiable factors that drive short-term depressive experiences. Our present study is motivated by the need for new evidence to inform interventions that are ecologically valid, timely, and responsive to people with different levels of depression risk.

A central strength of this study is its integration of intensive short-term data collection with extensive life-course phenotypic data. Existing EMA studies of depression have largely been conducted in opportunity or clinical samples without access to longitudinal background data [23], limiting their ability to contextualise short-term findings within the broader developmental context. In contrast, our study capitalises on the rich existing data of two large, well-characterised cohorts to select participants on the basis of previously established depression trajectory groups. This design enables a level of contextualisation that is not achievable in traditional EMA research, allowing short-term depressive dynamics to be examined against a backdrop of observed genetic, and potentially modifiable factors that may have accumulated across the life-course. This approach has the potential to yield insights into the real-time epidemiology of depression and ultimately to more time and person specific interventions and preventions.

A further strength lies in the multimodal nature of the data collection. We are collecting a variety of data encompassing sleep, diet, substance use, exercise, social interactions, daily activities, stress and other domains of mental health. This will allow us to thoroughly examine the epidemiology of depression in people with different depression trajectories for the first time. In addition, the combination of self-reported EMA data with passively collected objective physiological measures

For peer review only

such as sleep, activity and heart rate via wearable smartwatch devices provides a more complete picture of the biological and behavioural context in which depressive fluctuations occur than either data source could provide alone [35, 36, 43]. Self-report measures of some behaviours such as sleep and physical activity, while valuable, are subject to recall bias and may not accurately reflect objective physiological states [44]. Passive wearable data can help to validate such self-reported information, and together they can provide large amounts of powerful data that are suitable for advanced analytical methods such as machine learning and depression prediction [26, 45].

The aim of our study is to provide valuable insights into the epidemiology of depression and to inform the potential development of smartphone-based and ecological momentary interventions for depression [25]. Digital mental health interventions show considerable promise as scalable, accessible tools for improving depression outcomes, but their application is still very much in development. By identifying specific momentary risk and protective factors that strongly predict short-term depressive fluctuations, our study can provide a robust empirical foundation for the development of interventions that are triggered by, and responsive to, real-time signals of elevated risk. Such just-in-time adaptive interventions represent a promising frontier for depression treatment and prevention [23, 25].

Furthermore, we hope our study highlights a unique opportunity to conduct novel study designs that can be embedded within existing studies. Integrating intensive and/or passive smartphone and smartwatch data collection with the vast amount of data available in longitudinal cohort studies could hold significant opportunities to improve our understanding of depression and other diseases and behaviours.

Limitations

There are several limitations of our study that warrant acknowledgement. First, the EMA component captures depressive experience over a period of six weeks, providing a relatively brief window into short-term depressive fluctuations and not a continuous record of daily life over months or years. While such a study design is possible, the burden on participants to complete such measures over months or even years is likely to be too great. As such a key strength remains the ability to link these

For peer review only

short-term depressive symptoms with the longitudinal cohort data. Future studies could look to embed regular periodic assessments of EMA into existing longitudinal studies (i.e., once every couple of years) to address this.

Second, as with all EMA studies, our present design is likely to be subject to low compliance, participants rapidly completing the surveys and attrition over the study period. Participants who find the EMA schedule burdensome, or who experience significant worsening of depression during the study, may be less likely to complete all assessments or even consent to join the study to begin with, potentially introducing selection bias, as per some previous research [46-48]. Although our study protocol has been designed to minimise burden with surveys designed to take no more than a few minutes each, and appropriate incentives and support structures will be in place, some degree of non-random attrition is to be expected. Sensitivity analyses will be used to examine the potential impact of missing data on key findings.

Finally, while our study spans two different cohorts and is designed to maximise sample size within our available resources, statistical power for some of the more complex within-person and between-group analyses may be limited, particularly in the subsample completing smartwatches. Power analyses and simulation studies have been used to inform the study design but replication of key findings in independent samples will ultimately be necessary to establish their robustness.

For peer review only

Mood in the moment: an EMA study on depression

Figure 1

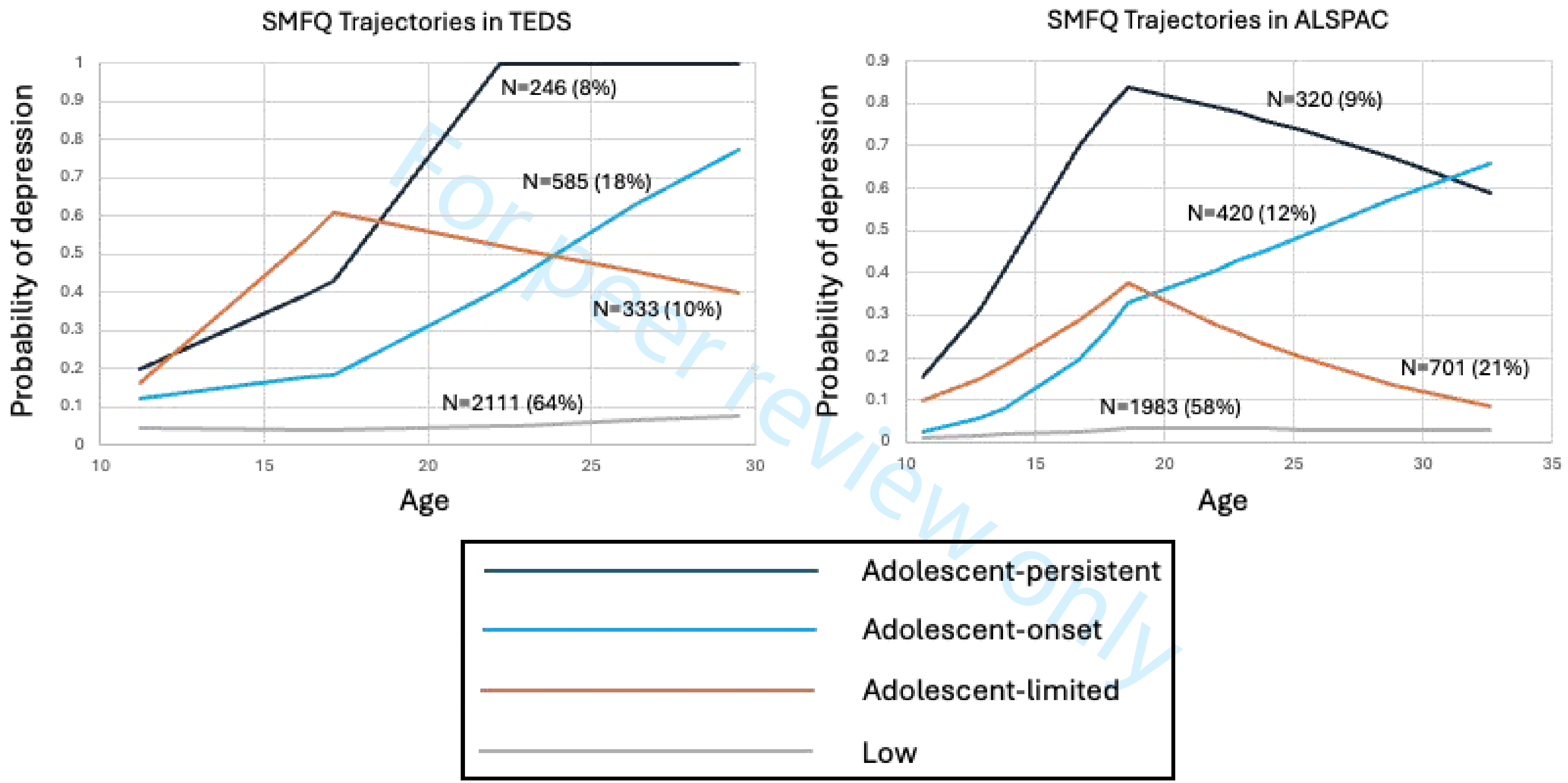

Figure 1. Depression trajectories in each cohort. TEDS: Twins Early Development Study; ALSPAC: Avon Longitudinal Study of Parents and Children. N (and percentage of total sample) are given for each trajectory.

For peer review only - http://bmjopen.bmj.com/site/about/guidelines.xhtml

Figure 2.

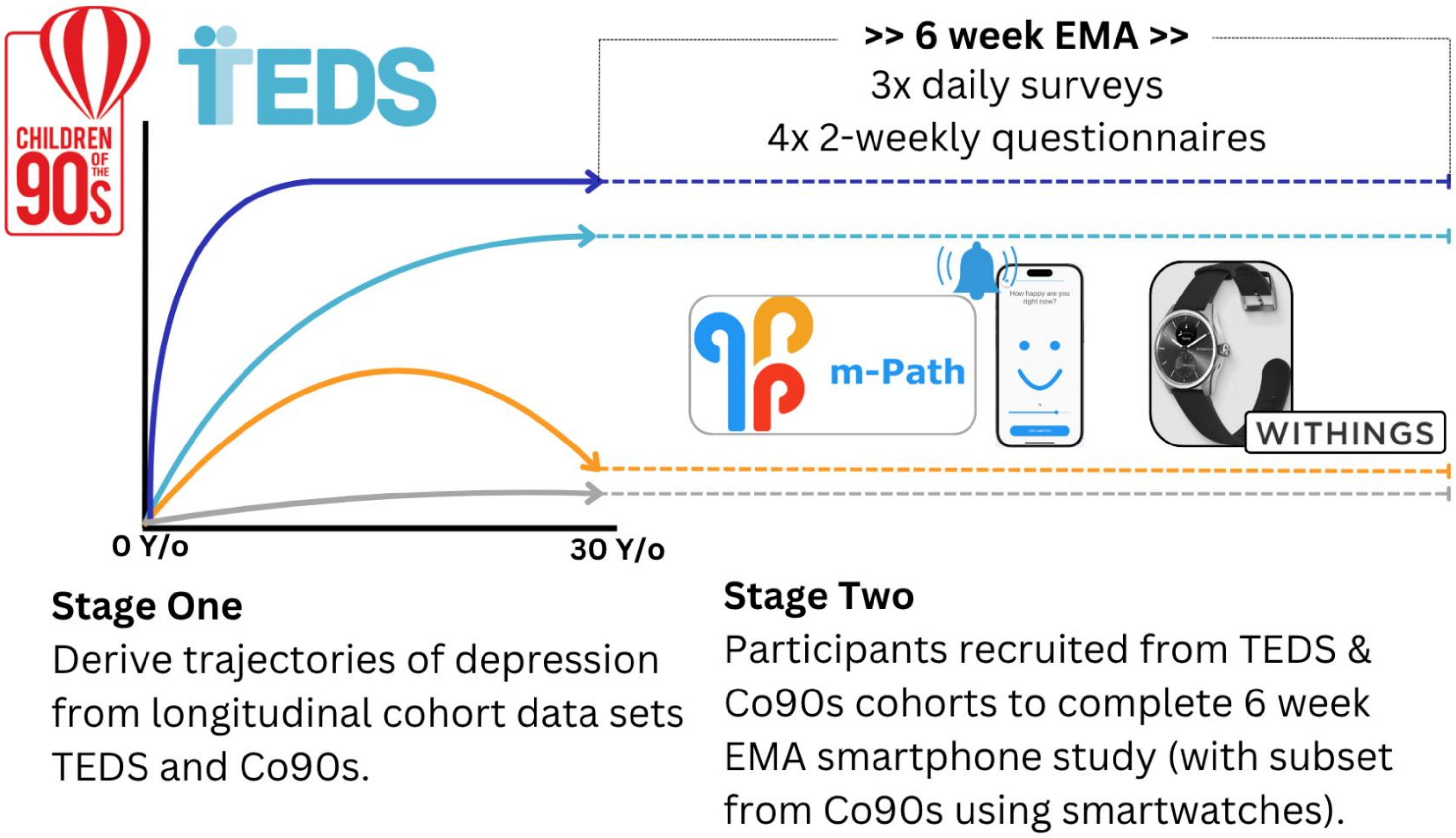

Figure 2. Summary of the Mood in the Moment Study design with schematic depression trajectories. TEDS: Twins Early Development Study; ALSPAC/Co90s: Avon Longitudinal Study of Parents and Children/Children of the 90s; EMA: Ecological Momentary Assessment.

Table 1. Study measures for the Mood in the Moment Study

Phase	Measure	Source(s) & Details	Items (n)	Data Collection Week						
**Pre-study**				**0**	**1**	**2**	**3**	**4**	**5**	**6**
	Informed consent		8	x	-	-	-	-	-	-
**EMA Questionnaires (baseline, two-week, four-week and end of survey only)**										
	Menstruation	Does participant currently menstruate	1	x	-	-	-	-	-	-
	Work pattern & caregiving	Adapted from prior shift-work studies; Typical vs. non-typical work pattern or caring responsibilities	1	x	-	-	-	-	-	-
	Depression	Short Mood and Feelings Questionnaire (SMFQ)[49]	13	x	-	x	-	x	-	x
	Anxiety	Generalised Anxiety Disorder Scale (GAD 7)[50]	7	x	-	x	-	x	-	x
	Resilience	Brief Resilience Scale (BRS)[51]	6	x	-	#	-	#	-	#
	Self esteem	Rosenberg Self-Esteem Scale (RSES)[52]	10	x	-	#	-	#	-	#
	Cognitive style	Global Cognitive Styles Questionnaire (CSQ)[53]	4	x	-	#	-	#	-	#
	Social support	Social Support Scale[54]	6	x	-	x	-	x	-	x
	Exercise	Adapted from prior studies; frequency of mild, moderate, & strenuous exercise	3	x	-	x	-	x	-	x
	Time outdoors	Newly developed; typical daily duration	1	x	-	x	-	x	-	x
	Sleep	Adapted from Pittsburgh Sleep Quality Index (PSQI); sleep quality & impact on daily life[55]	1 to 2*	x	-	x	-	x	-	x
	Pain	Newly developed; pain frequency & impact on life or work	1 to 2*	x	-	x	-	x	-	x
	Recent Life Events	Adapted from prior life events studies; occurrence of positive & negative life events	1	x	-	x	-	x	-	x
	Hormonal or contraceptive medication	Newly developed; medication type & duration of use	1 to 3*	x	-	x	-	x	-	x
	Gynaecological or reproductive health conditions	Newly developed; history of conditions	1	x	-	-	-	-	-	-
	Mental health conditions	Newly developed; history & type of mental health conditions	1 to 2*	x	-	-	-	-	-	-
	Mental health medication & treatment	Newly developed; recent use of mental health medication or treatment	1	x	-	x	-	x	-	x
	Perceived stress**	Perceived Stress Scale (PSS)[56]	14			x				

For peer review only

	Personality**	Big Five Inventory – Short Form (BFI-SF)[57]	15					x		
	Burnout**	Maslach Burnout Inventory (MBI-GS)[58]	16							x
**EMA Surveys (asking about last 3 hours, 3 x day)**										
	Depression***	Adapted version of Patient Health Questionnaire (PHQ-8/9)[59-61]	8 or 9	-	ooo	ooo	ooo	ooo	ooo	ooo
	Anxiety	Adapted version of Generalised Anxiety Disorder Scale (GAD-2) + 1 item on irritability from GAD-7[50]	3	-	ooo	ooo	ooo	ooo	ooo	ooo
	Current location	Where participant has spent most time	1	-	ooo	ooo	ooo	ooo	ooo	ooo
	Current activities	What participant has mostly been doing	1	-	ooo	ooo	ooo	ooo	ooo	ooo
	Diet and food quality	Adapted from prior diet and food insecurity studies; Food consumed & type of food	1 to 2*	-	ooo	ooo	ooo	ooo	ooo	ooo
	Physical activity	Highest level of physical activity (from no movement to strenuous exercise)	1	-	ooo	ooo	ooo	ooo	ooo	ooo
	Substance use	Smoking, vaping, & alcohol consumption	1	-	ooo	ooo	ooo	ooo	ooo	ooo
	Stress	Feelings of stress	1	-	ooo	ooo	ooo	ooo	ooo	ooo
	Confiding in someone	Feeling able to confide in someone close[62]	1	-	ooo	ooo	ooo	ooo	ooo	ooo
	Social connectedness	Newly developed; enjoyment of social contact online, in-person, & via social media	3	-	ooo	ooo	ooo	ooo	ooo	ooo
	Sleep	Adapted from Pittsburgh Sleep Quality Index (PSQI); sleep quality, sleep & wake time[55]	1 to 3*	-	ooo	ooo	ooo	ooo	ooo	ooo
	Daily Menstruation status****	Has period started in past 24 hours	1	-	ooo	ooo	ooo	ooo	ooo	ooo
**Passive Data Collection in ALSPAC only**										
	Withings Steel HR or ScanWatch Light Smart Watch	Step count, heart rate and sleep		Continuous throughout 6 weeks						

Note. x = Administered at baseline or follow up
# = Administered at baseline or follow up in TEDS only
ooo = Administered three times a day across each week.
* Follow-up items dependent on answers provided to initial item.
** Only administered within ALSPAC.
*** PHQ-9 administered 1x per day in afternoon survey, PHQ-8 administered in morning and evening survey.
**** Only administered in evening survey to participants who indicated they menstruated in baseline questionnaire.

For peer review only

Acknowledgments

We gratefully acknowledge the ongoing contribution of the Twins Early Development Study (TEDS) participants and their families. We are extremely grateful to all the ALSPAC families who took part in this study, the midwives for their help in recruiting them, and the whole ALSPAC team, which includes interviewers, computer and laboratory technicians, clerical workers, research scientists, volunteers, managers, receptionists and nurses.

Contributions

Conceptualisation: ASFK. Funding acquisition: ASFK. Methodology: All authors. Writing original draft: ASFK. Writing review and editing: All authors.

Funding

This research was funded by Wellcome (Grant ref: 227063/Z/23/Z). The UK Medical Research Council and Wellcome (Grant ref: MR/Z505924/1) and the University of Bristol provide core support for ALSPAC. A comprehensive list of grants funding is available on the ALSPAC website: http://www.bristol.ac.uk/alspac/external/documents/grant-acknowledgements.pdf. TEDS is supported by the UK Medical Research Council (MR/V012878/1 and previously MR/M021475/1). This publication is the work of the authors and ASFK will serve as guarantor for the contents of this paper. ASFK is supported by a Wellcome Early Career Award (Grant ref: 227063/Z/23/Z).

Data availability

The informed consent obtained from ALSPAC (Avon Longitudinal Study of Parents and Children) participants does not allow the data to be made available through any third party maintained public repository. Supporting data are available from ALSPAC on request under the approved proposal number, B4792. Full instructions for applying for data access can be found here: http://www.bristol.ac.uk/alspac/researchers/access/. The ALSPAC study website contains details of all available data (http://www.bristol.ac.uk/alspac/researchers/our-data/).

Competing interests

For peer review only

Mood in the moment: an EMA study on depression

None

Patient and public involvement

Patients and the public were involved in the acquisition of funding, design and dissemination plans of this research.

Patient consent for publication

Not applicable

For peer review only

References

1. W.H.O, *World mental health today: latest data*. 2025.
2. W.H.O. *Depression: let's talk*. 2017 [cited 2017.
3. Thapar A, et al., *Depression in young people.* Lancet, 2022. **400**(10532): p. 617-631.
4. Armitage, J.M., et al., *Cross-cohort change in parent-reported emotional problem trajectories across childhood and adolescence in the UK.* Lancet Psychiatry, 2023. **10**(7): p. 509-517.
5. McGorry, P.D., et al., *The Lancet Psychiatry Commission on youth mental health.* Lancet Psychiatry, 2024. **11**(9): p. 731-774.
6. Lopez-Lopez, J.A., et al., *Trajectories of depressive symptoms and adult educational and employment outcomes.* BJPsych Open, 2019. **6**(1): p. e6.
7. Weavers, B., et al., *The antecedents and outcomes of persistent and remitting adolescent depressive symptom trajectories: a longitudinal, population-based English study.* Lancet Psychiatry, 2021. **8**(12): p. 1053-1061.
8. Copeland, W.E., et al., *Adult Functional Outcomes of Common Childhood Psychiatric Problems: A Prospective, Longitudinal Study.* JAMA Psychiatry, 2015. **72**(9): p. 892-9.
9. Fergusson, D.M., J.M. Boden, and J. Horwood, *Recurrence of major depression in adolescence and early adulthood, and later mental health, educational and economic outcomes.* Br J Psychiatry, 2007. **191**: p. 335-342.
10. Solmi, M., et al., *Age at onset of mental disorders worldwide: large-scale meta-analysis of 192 epidemiological studies.* Mol Psychiatry, 2022. **27**(1): p. 281-295.
11. Desai Bostrom, A.E., et al., *Recovery and Recurrence From Major Depression in Adolescence and Adulthood.* Acta Psychiatr Scand, 2025. **151**(5): p. 625-633.
12. Shore, L., et al., *Review: Longitudinal trajectories of child and adolescent depressive symptoms and their predictors - a systematic review and meta-analysis.* Child and Adolescent Mental Health, 2018. **23**(2): p. 107-120.
13. Schubert, K.O., et al., *Depressive symptom trajectories in late adolescence and early adulthood: A systematic review.* Aust N Z J Psychiatry, 2017. **51**(5): p. 477-499.
14. Musliner, K.L., et al., *Heterogeneity in long-term trajectories of depressive symptoms: Patterns, predictors and outcomes.* J Affect Disord, 2016. **192**: p. 199-211.
15. Ge, X. and M.N. Natsuaki, *In Search of Explanations for Early Pubertal Timing Effects on Developmental Psychopathology.* Current Directions in Psychological Science, 2009. **18**(6).
16. Kwong, A.S.F., et al., *Identifying Critical Points of Trajectories of Depressive Symptoms from Childhood to Young Adulthood.* J Youth Adolesc, 2019. **48**: p. 815-827.
17. Kwong, A.S.F., et al., *Polygenic risk for depression, anxiety and neuroticism are associated with the severity and rate of change in depressive symptoms across adolescence.* Journal of Child Psychology and Psychiatry, 2021.
18. Grimes, P.Z., et al., *Genetic Architectures of Adolescent Depression Trajectories in 2 Longitudinal Population Cohorts.* JAMA Psychiatry, 2024.

For peer review only

19. Cadman, T., et al., *Joint associations of parental personality traits and socio-economic position with trajectories of offspring depression: Findings from up to 6925 families in a UK birth cohort.* JCPP Adv, 2021. **1**(3): p. e12028.
20. Rajyaguru, P., et al., *Maternal and paternal depression and child mental health trajectories: evidence from the Avon Longitudinal Study of Parents and Children.* BJPsych Open, 2021. **7**(5): p. e166.
21. Fritz, J., et al., *So You Want to Do ESM? 10 Essential Topics for Implementing the Experience-Sampling Method.* Advances in Methods and Practices in Psychological Science, 2024. **7**(3): p. 1-27.
22. Siepe, B.S., et al., *Understanding Ecological-Momentary-Assessment Data: A Tutorial on Exploring Item Performance in Ecological-Momentary-Assessment Data.* Advances in Methods and Practices in Psychological Science, 2025. **8**(1): p. 1-20.
23. Harari, G.M. and S.D. Gosling, *Understanding behaviours in context using mobile sensing.* Nature Reviews Psychology, 2023. **2**(12): p. 767-779.
24. Dejonckheere, E., et al., *Complex affect dynamics add limited information to the prediction of psychological well-being.* Nat Hum Behav, 2019. **3**(5): p. 478-491.
25. Fried, E.I., R.K.K. Proppert, and C.L. Rieble, *Building an Early Warning System for Depression: Rationale, Objectives, and Methods of the WARN-D Study.* Clin Psychol Eur, 2023. **5**(3): p. e10075.
26. Ware, S., et al., *Predicting depressive symptoms using smartphone data.* Smart Health, 2020. **15**.
27. Volovic-Shushan, S., et al., *Real-time experience: A systematic review of ecological momentary assessments of everyday functioning in major depressive disorder.* Aust Occup Ther J, 2026. **73**(2): p. e70080.
28. Unzueta Saavedra, J., et al., *Smartphone Ecological Momentary Assessment and Wearable Activity Tracking in Pediatric Depression: Cohort Study.* JMIR Form Res, 2025. **9**: p. e66187.
29. Triantafillou, S., et al., *Relationship Between Sleep Quality and Mood: Ecological Momentary Assessment Study.* JMIR Ment Health, 2019. **6**(3): p. e12613.
30. El-Awad, U., et al., *Physical activity is associated with subsequent affective well-being in day hospital patients with a diagnosis of depression: A combined ecological momentary assessment and accelerometry study.* Mental Health and Physical Activity, 2025. **29**.
31. Li, Y.M., et al., *Temporal associations between objectively measured physical activity and depressive symptoms: An experience sampling study.* Front Psychiatry, 2022. **13**: p. 920580.
32. Perski, O., et al., *Understanding health behaviours in context: A systematic review and meta-analysis of ecological momentary assessment studies of five key health behaviours.* Health Psychol Rev, 2022. **16**(4): p. 576-601.
33. Kuczynski, A.M., et al., *Characterizing the momentary association between loneliness, depression, and social interactions: Insights from an ecological momentary assessment study.* J Affect Disord, 2024. **360**: p. 376-386.
34. Speyer, L.G., et al., *The role of moment-to-moment dynamics of perceived stress and negative affect in co-occurring ADHD and internalising symptoms.* J Autism Dev Disord, 2023. **53**(3): p. 1213-1223.

For peer review only

35. Price, G.D., et al., *Use of passively collected actigraphy data to detect individual depressive symptoms in a clinical subpopulation and a general population.* J Psychopathol Clin Sci, 2025. **134**(1): p. 31-40.
36. Price, G.D., et al., *Detecting major depressive disorder presence using passively-collected wearable movement data in a nationally-representative sample.* Psychiatry Res, 2024. **332**: p. 115693.
37. Lockhart, C., et al., *Twins Early Development Study (TEDS): A genetically sensitive investigation of mental health outcomes in the mid-twenties.* JCPP Adv, 2023. **3**(2): p. e12154.
38. Boyd, A., et al., *Cohort Profile: the 'children of the 90s'--the index offspring of the Avon Longitudinal Study of Parents and Children.* Int J Epidemiol, 2013. **42**(1): p. 111-27.
39. Fraser, A., et al., *Cohort Profile: the Avon Longitudinal Study of Parents and Children: ALSPAC mothers cohort.* Int J Epidemiol, 2013. **42**(1): p. 97-110.
40. Northstone, K., et al., *The Avon Longitudinal Study of Parents and Children (ALSPAC): an update on the enrolled sample of index children in 2019.* Wellcome Open Res, 2019. **4**: p. 51.
41. Grimes, P.Z., et al., *Genome-wide association study of adolescent-onset depression.* Under review.
42. Murray, A., et al., *Illuminating the daily life experiences of adolescents with and without ADHD: protocol for an ecological momentary assessment study.* BMJ Open, 2023. **13**(9): p. e077222.
43. Ringwald, W.R., et al., *Passive Smartphone Sensors for Detecting Psychopathology.* JAMA Netw Open, 2025. **8**(7): p. e2519047.
44. Lekkas, D., et al., *Depression deconstructed: Wearables and passive digital phenotyping for analyzing individual symptoms.* Behav Res Ther, 2023. **168**: p. 104382.
45. Kim, E.Y., et al., *Diagnosis of major depressive disorder by combining multimodal information from heart rate dynamics and serum proteomics using machine-learning algorithm.* Prog Neuropsychopharmacol Biol Psychiatry, 2017. **76**: p. 65-71.
46. Murray, A., et al., *Predicting Participation Willingness in Ecological Momentary Assessment of General Population Health and Behavior: Machine Learning Study.* J Med Internet Res, 2023. **25**: p. e41412.
47. Murray, A., et al., *Respondent characteristics associated with adherence in a general population ecological momentary assessment study.* International Journal of Methods in Psychiatric Research, 2023. **32**(4).
48. Murray, A.L., et al., *Prompt-level predictors of compliance in an ecological momentary assessment study of young adults' mental health.* J Affect Disord, 2023. **322**: p. 125-131.
49. Angold, A., et al., *Development of a short questionnaire for use in epidemiological studies of depression in children and adolescents.* International Journal of Methods in Psychiatric Research, 1995. **5**(4): p. 237-249.
50. Spitzer, R.L., et al., *A Brief Measure for Assessing Generalized Anxiety Disorder.* Arch Intern Med, 2006. **166**: p. 1092-1097.
51. Smith, B., et al., *The Brief Resilience Scale: Assessing the Ability to Bounce Back.* International Journal of Behavioral Medicine, 2008. **15**: p. 194-200.
52. Rosenberg, M., *Rosenberg self-esteem scale.* Journal of Religion and Health, 1965.

For peer review only

53. Pearson, R.M., et al., *Association Between Maternal Depressogenic Cognitive Style During Pregnancy and Offspring Cognitive Style 18 Years Later.* Am J Psychiatry, 2013. **170**(4): p. 434-441.
54. Shakespeare-Finch, J. and P.L. Obst, *The Development of the 2-Way Social Support Scale: A Measure of Giving and Receiving Emotional and Instrumental Support.* Journal of Personality Assessment, 2011. **93**(483-490).
55. Buysse, D.J., et al., *The Pittsburgh Sleep Quality Index: a new instrument for psychiatric practice and research.* Psychiatry Research, 1989. **28**(2): p. 193-213.
56. Cohen, S., T. Kamarck, and R. Mermelstein, *A global measure of perceived stress.* Journal of Health and Social Behavior, 1983. **24**(4): p. 385-396.
57. Lang, F.R., et al., *Short assessment of the Big Five: robust across survey methods except telephone interviewing.* Behavior research methods, 2011. **43**(2): p. 548–567.
58. Maslach, C. and S.E. Jackson, *The Measurement of Experienced Burnout.* Journal of Occupational Behaviour, 1981. **2**(2): p. 99-113.
59. Kroenke, K., R.L. Spitzer, and J.B. Williams, *The PHQ-9: Validity of a Brief Depression Severity Measure.* J Gen Intern Med, 2001. **16**(9): p. 606-613.
60. Kroenke, K., et al., *The PHQ-8 as a measure of current depression in the general population.* Journal of Affective Disorders, 2009. **114**(1): p. 163-173.
61. Haddox, D., et al., *Psychometric properties and validity of a Mobile Patient Health Questionnaire-9 (MPHQ-9) for ecological momentary assessment in depressed adults.* Psychol Assess, 2025.
62. Choi, K.W., et al., *An Exposure-Wide and Mendelian Randomization Approach to Identifying Modifiable Factors for the Prevention of Depression.* Am J Psychiatry, 2020. **177**(10): p. 944-954.

For peer review only

Supplementary materials

Mood in the Moment: a study protocol for embedding ecological momentary assessments into established longitudinal cohorts to examine depression in real-time

Additional cohort information - ALSPAC

Pregnant women resident in Avon, UK with expected dates of delivery between 1st April 1991 and 31st December 1992 were invited to take part in the study. 20,248 pregnancies have been identified as being eligible and the initial number of pregnancies enrolled was 14,541. Of the initial pregnancies, there was a total of 14,676 foetuses, resulting in 14,062 live births and 13,988 children who were alive at 1 year of age. When the oldest children were approximately 7 years of age, an attempt was made to bolster the initial sample with eligible cases who had failed to join the study originally. As a result, when considering variables collected from the age of seven onwards (and potentially abstracted from obstetric notes) there are data available for more than the 14,541 pregnancies mentioned above: The number of new pregnancies not in the initial sample (known as Phase I enrolment) that are currently represented in the released data and reflecting enrolment status at the age of 24 is 906, resulting in an additional 913 children being enrolled (456, 262 and 195 recruited during Phases II, III and IV respectively). The phases of enrolment are described in more detail in the cohort profile paper and its update. The total sample size for analyses using any data collected after the age of seven is therefore 15,447 pregnancies, resulting in 15,658 foetuses. Of these 14,901 children were alive at 1 year of age. From the age of 30, there were an additional 101 new participants recruited from 101 pregnancies during Phase V enrolment. The total sample size for analyses using any data collected after the age of 30 is therefore 15,548 pregnancies, resulting in 15,690 foetuses. Of these 15,002 children were alive at 1 year of age. Study data were collected and managed using REDCap (Research Electronic Data Capture) electronic data capture tools hosted at the University of Bristol. REDCap is a secure, web-based software platform designed to support data capture for research studies (Harris et al., 2009).

Reference: Harris, P., et al., *Research electronic data capture (REDCap) – A metadata-driven methodology and workflow process for providing translational research informatics support.* J Biomed Inform, 2009. **42**(2): p. 377-81.

For peer review only

Survey Measures used in Mood and the Moment Study

EMA Schedule and Key Information

Brief Daily Surveys (3 x per day unless stated)

Number of measures per survey: 11 – 13.

Approx time to complete: 3 minutes

The content of the 3 x daily brief daily surveys is almost identical except for order of administration. A summary of each survey (morning, afternoon, evening) is provided below, along with timings of the survey notifications and reminders sent in the m-Path app. A brief description of each survey item and its application in the current EMA study follows, and a detailed description of all survey and questionnaire items including item and response wording and references are provided at the end of the document.

Morning Survey Order	Afternoon Survey Order	Evening Survey Order
Current location and activity	PHQ-9	GAD-2
Diet	Current location and activity	Confiding in others
PHQ-8	Diet	Social connectedness
GAD-2	Exercise	Social media
Sleep	Substance use	Menstruation
Exercise	GAD-2	PHQ-8
Substance use	Stress	Stress
Stress	Positive and negative affect	Positive and negative affect
Positive and negative affect	Social media use	Current location and activity
Confiding in others	Social connectedness	Sleep*
Social connectedness	Confiding in others	Substance use
Social media use	Sleep*	Exercise
		Diet
**Notification time: 8:00am to 9:00am**	**Notification time: 1:00pm to 2:00pm**	**Notification time: 6:00pm to 7:00pm**
**Survey open for 3 hour**	**Survey open for 3 hour**	**Survey open for 3 hour**
**Reminders x 2, every 60 minutes**	**Reminders x 2, every 60 minutes**	**Reminders x 2, every 60 minutes**
	***only asked if previously missed**	***only asked if previously missed**

PHQ-8

- Administered with morning and evening prompts only
- 8 items
- Clinically validated measure
- Adapted version for EMA: replaced ‘Over the last 2 weeks’ with ‘In the last 3 hours’

PHQ 9

- Administered with afternoon prompt only
- 9 items

- Clinically validated measure
- Adapted version for EMA: replaced ‘Over the last 2 weeks’ with ‘In the last 3 hours’

GAD-2 (+ irritability item from GAD-7)

- 3 items
- Clinically validated measure
- Adapted version for EMA: replaced ‘Over the last 2 weeks’ with ‘In the last 3 hours’

Confiding in others

- 1 item
- Validated measure

Positive and negative affect

- 2 items
- Developed by research team at UofE

Stress

- 1 item
- Developed by research team at UofE

Sleep

- 3 items total
- Validated measure: single item on sleep quality from PSQI + 2 items on sleep/awake time
- **Once a day only**

Diet (food quality)

- 2 items
- Developed by research team at UofE

Menstruation

- Administered with evening prompt only
- 1 item
- Developed by research team at UofE

Exercise

- 1 item

For peer review only

- Developed by research team at UofE

Substance use (smoking/vaping & alcohol)

- 1 item
- Developed by research team at UofE

Current location & current activity

- 2 items
- Developed by research team at UofE

Social connectedness

- 2 items
- Developed by research team at UofE

Social media use

- 1 item
- Developed by research team at UofE

Baseline, week-2, -4 and -6 exit Questionnaires

Approx time to complete: 5 minutes

A summary of the questionnaire content is provided below, along with timings of the questionnaire notifications and reminders sent in the m-Path app. A brief description of each questionnaire item and its application in the current study follows, and a detailed description of all survey and questionnaire items including item and response wording and references are provided at the end of the document.

Short mood and feelings questionnaire (SMFQ)

- 13 items
- Clinically validated measure
- Assessed at every sweep

GAD-7

- 7 items
- Clinically validated measure
- Assessed at every sweep

For peer review only

Brief Resilience Scale (BRS)

- 6 items
- Validated measure
- ***Baseline questionnaire only***

Rosenberg Self-Esteem Scale (RSE)

- 10 items
- Validated measure
- ***Baseline questionnaire only***

Global cognitive styles

- 4 items
- Validated measure
- ***Baseline questionnaire only***

Perceived Stress Scale

- 14 items
- Validated measure
- ***2 week follow up questionnaire only***

Big Five Inventory 15 Item Version

- 15 items
- Validated measure
- ***4 week follow up questionnaire only***

Maslach Burnout Inventory – MBI-GS

- 16 items
- Validated measure
- ***6 week follow up questionnaire only***

Hormonal Contraception/Medication Use

- *3 items*
- Developed by research team at UofE
- Assessed at every sweep

Gynaecological or Reproductive Health Conditions

For peer review only

- *1 item*
- Developed by research team at UofE
- ***Baseline questionnaire only***

Pain

- 2 items
- Developed by research team at UofE
- Assessed at every sweep

Working pattern

- 1 item
- Developed by research team at UofE
- Assessed at every sweep

Exercise

- 3 items
- Developed by research team at UofE
- Assessed at every sweep

Time spent outdoors

- 1 item
- Developed by research team at UofE
- Assessed at every sweep

Social Support

- 6 items
- Validated measure
- ***Baseline questionnaire only***

Life in the last 2 weeks (exercise, sleep, pain and life events)

- 9 items
- Developed by research team at UofE
- Assessed at every sweep

Antidepressant medication or therapy use in the last 2 weeks

- 1 item
- Developed by research team at UofE
- Assessed at every sweep

For peer review only

Current and previous history of mental health

- 1 item
- Developed by research team at UofE
- ***Baseline questionnaire only***

Exit survey (only at week 6 at the end of the study)

- 1 item
- Developed by research team at UofE

For peer review only

EMA items (Validated Measures from Surveys and Questionnaires)

Adapted Patient Health Questionnaire (PHQ-8 and PHQ-9) (Kroenke et al. 2001)

The PHQ-8 is an 8-item self-administered measure which can be used to screen for depression and assess the severity of depressive symptoms in the previous two weeks. Here we adapt the time frame for the items to ask, ***'In the last 3 hours,*** *I have…' to make it suitable for use as an EMA measure*, and we adapt the sleep question to, 'Last night, I had trouble with sleep' (included in morning survey only). The PHQ-8 is included in the morning and evening surveys.

The PHQ-9, administered in the afternoon survey only, includes an additional item assessing suicidality. Participants answer using a sliding scale with a range between 0 and 100 with the further option for prefer not to say.

Participants will be informed in the participant information sheet and the consent form that we will not be actively monitoring their responses and as such they cannot use the m-Path app and their response to this item to attain help and support. However, if this question is answered positively (i.e., ≥ 1), participants will instantly be directed to help lines and advised to seek support through the application before being able to proceed with the survey.

PHQ-8 and PHQ-9 items [daily surveys]:

In the last 3 hours, I have **had little interest or pleasure in doing things**

0. Not at all

to

100. Constantly

Prefer not to say

In the last 3 hours, I have **felt down, depressed, or hopeless**

0. Not at all

to

100. Constantly

For peer review only

Prefer not to say

Last night, I **had trouble with sleep**

0. Not at all

to

100. Constantly

Prefer not to say

In the last 3 hours, I have **felt tired or have had little energy**

0. Not at all

to

100. Constantly

Prefer not to say

In the last 3 hours, I have **had poor appetite or have been overeating**

0. Not at all

to

100. Constantly

Prefer not to say

In the last 3 hours, I have **felt bad about myself**

0. Not at all

to

100. Constantly

Prefer not to say

In the last 3 hours, I have **had trouble concentrating**

0. Not at all

to

For peer review only

100. Constantly

Prefer not to say

In the last 3 hours, I have **been moving or speaking slowly, or fidgeting more**

0. Not at all

to

100. Constantly

Prefer not to say

In the last 3 hours, I have **had thoughts of hurting myself or that I would be better off dead** *[PHQ-9 question, included in the afternoon survey only. Participant directed to support lines if respond with answers ≥1]*

0. Not at all

to

100. Constantly

Prefer not to say

Reference: Kroenke, K., Spitzer, R. L., & Williams, J. B. W. (2001). The PHQ-9 - Validity of a brief depression severity measure. Journal of General Internal Medicine, 16, 606-613.

Generalised Anxiety Disorder Scale [Seven-Item/Two-item version] (GAD-7/GAD-2) (Spitzer et al. 2006)

The GAD-7 is a seven-item measure which screens for anxiety severity in clinical and general populations. The two-item version (GAD-2) uses the first two items of the GAD-7 to assess critical facets of anxiety disorders. This shorter measure has shown to have good sensitivity and specificity to that of the GAD-7 (Plummer et al. 2016). Here we use the GAD-2 along with an additional question on irritability from the GAD-7, to be administered in all 3 brief daily surveys. We adapt the time frame for the items to ask, *'**In the last 3 hours,** have you…' to make it suitable for use as an EMA measure,* as with the PHQ-8/9 above.

Participants will also be administered the full GAD-7 during the baseline questionnaire and at every 2-week follow up questionnaire. The original item framing of '*Over the last 2 weeks...*'

For peer review only

will be used for administering the GAD-7 in these bi-weekly questionnaires, but for ease of comprehension we have removed the "been bothered with" phrasing in each question.

GAD-2 (Adapted for brief daily surveys)

Generalised Anxiety Disorder 2 + irritability [daily surveys]

In the last 3 hours, have you been **feeling nervous, anxious or on edge?**

0. Not at all

to

100. Constantly

Prefer not to say

In the last 3 hours, have you been **unable to stop or control worrying?**

0. Not at all

to

100. Constantly

Prefer not to say

In the last 3 hours, have you been **feeling easily annoyed or irritable?**

0. Not at all

to

100. Constantly

Prefer not to say

Generalised Anxiety Disorder 7 (Full version included in bi-weekly questionnaires)

In the past 2 weeks, **have you been feeling nervous, anxious or on edge?**

0. Not at all

1. Some time

2. More than half the time

For peer review only

3. Nearly all the time

4. Prefer not to say

In the past 2 weeks, **have you been unable to stop or control worrying?**

0. Not at all

1. Some time

2. More than half the time

3. Nearly all the time

4. Prefer not to say

In the past 2 weeks, **have you been worrying too much about different things?**

0. Not at all

1. Some time

2. More than half the time

3. Nearly all the time

4. Prefer not to say

In the past 2 weeks, **have you had trouble relaxing?**

0. Not at all

1. Some time

2. More than half the time

3. Nearly all the time

4. Prefer not to say

In the past 2 weeks, **have you been feeling so restless that it is hard to sit still?**

0. Not at all

1. Some time

2. More than half the time

3. Nearly all the time

For peer review only

4. Prefer not to say

In the past 2 weeks, **have you been feeling easily annoyed or irritable?**

0. Not at all

1. Some time

2. More than half the time

3. Nearly all the time

4. Prefer not to say

In the past 2 weeks, **have you been feeling afraid as if something awful might happen?**

0. Not at all

1. Some time

2. More than half the time

3. Nearly all the time

4. Prefer not to say

Reference: Spitzer, R. L., Kroenke, K., Williams, J. B., & Löwe, B. (2006). A brief measure for assessing generalized anxiety disorder: the GAD-7. Archives of internal medicine, 166(10), 1092-1097.

Confiding in Someone (Choi et al. 2020)

Participants are asked a single item *'In the last 3 hours, have you felt like you could confide in someone close to you?'*. This question is included in all 3 brief daily surveys.

In the last 3 hours, **have you *felt like* you could confide in someone close to you?**

0. Not at all

1. Some of the time

2. More than half the time

3. Nearly all the time

For peer review only

4. Prefer not to say

Reference: Choi, K. W., Stein, M. B., Nishimi, K. M., Ge, T., Coleman, J. R. I., Chen, C. Y., Ratanatharathorn, A., Zheutlin, A. B., Dunn, E. C., 23andMe Research Team, Major Depressive Disorder Working Group of the Psychiatric Genomics Consortium, Breen, G., Koenen, K. C., & Smoller, J. W. (2020). An Exposure-Wide and Mendelian Randomization Approach to Identifying Modifiable Factors for the Prevention of Depression. The American journal of psychiatry, 177(10), 944–954. https://doi.org/10.1176/appi.ajp.2020.19111158

Social Support Scale (Shakespeare-Finch & Obst, 2011)

The social support scale is a six-item scale to assess self-reported/perceived social supports. This measure will be administered at baseline only.

There is someone I can talk to about the pressures in my life

1. Very frequently
2. Occasionally
3. Rarely
4. Very rarely
5. Never
6. Prefer not to answer

There is at least one person that I could share most things with

1. Very frequently
2. Occasionally
3. Rarely
4. Very rarely
5. Never
6. Prefer not to answer

When I am feeling down, there is someone I can lean on

1. Very frequently
2. Occasionally
3. Rarely
4. Very rarely
5. Never
6. Prefer not to answer

There is someone in my life that I can get emotional support from

1. Very frequently
2. Occasionally
3. Rarely
4. Very rarely
5. Never
6. Prefer not to answer

For peer review only

There is at least one person that I feel I can trust

1. Very frequently
2. Occasionally
3. Rarely
4. Very rarely
5. Never
6. Prefer not to answer

There is someone in my life that makes me feel worthwhile

1. Very frequently
2. Occasionally
3. Rarely
4. Very rarely
5. Never
6. Prefer not to answer

Reference: Shakespeare-Finch, J., & Obst, P.L. (2011). The Development of the 2-Way Social Support Scale: A Measure of Giving and Receiving Emotional and Instrumental Support. Journal of Personality Assessment, 93, 483 - 490.

Short Mood and Feelings Questionnaire (SMFQ) (Angold & Costello, 1987)

The SMFQ is a 13-item self-report questionnaire in which participants answer items to assess key depression symptoms. This measure has been used in clinical and general populations. Participants will be administered the SMFQ at baseline and at every two-week follow-up questionnaire.

In the past two weeks, **I felt miserable or unhappy**

0. Not true
1. Sometimes
2. True
3. Prefer not to say

In the past two weeks, **I didn't enjoy anything at all**

0. Not true
1. Sometimes
2. True
3. Prefer not to say

In the past two weeks, **I felt so tired I just sat around and did nothing**

0. Not true
1. Sometimes
2. True
3. Prefer not to say

For peer review only

In the past two weeks, **I was very restless**

0. Not true
1. Sometimes
2. True
3. Prefer not to say

In the past two weeks, **I felt I was no good anymore**

0. Not true
1. Sometimes
2. True
3. Prefer not to say

In the past two weeks, **I cried a lot**

0. Not true
1. Sometimes
2. True
3. Prefer not to say

In the past two weeks, **I found it hard to think properly or concentrate**

0. Not true
1. Sometimes
2. True
3. Prefer not to say

In the past two weeks, **I hated myself**

0. Not true
1. Sometimes
2. True
3. Prefer not to say

This is a quality control question, please select **'True'**

1. Not true
2. Sometimes
3. True
4. Prefer not to say

In the past two weeks, **I was a bad person**

0. Not true
1. Sometimes
2. True
3. Prefer not to say

In the past two weeks, **I felt lonely**

0. Not true
1. Sometimes
2. True
3. Prefer not to say

For peer review only

In the past two weeks, **I thought nobody really loved me**

1. Not true
2. Sometimes
3. True
4. Prefer not to say

In the past two weeks, **I thought I could never be as good as other people**

0. Not true
1. Sometimes
2. True
3. Prefer not to say

In the past two weeks, **I did everything wrong**

0. Not true
1. Sometimes
2. True
3. Prefer not to say

Reference: Angold, A., & Costello, E. J. (1987). *Short Mood and Feelings Questionnaire (SMFQ, SMFQ-C, SMFQ-P)* [Database record]. APA PsycTests. https://doi.org/10.1037/t15197-000

Brief Resilience Scale (BRS) (Smith et al. 2008)

The BRS is a brief self-report scale in which participants answer six items to measure their ability to bounce back or recover from stress. This measure will be administered at baseline only.

I tend to bounce back quickly after hard times

1. Strongly disagree
2. Disagree
3. Neither agree nor disagree
4. Agree
5. Strongly agree
6. Prefer not to say

I have a hard time making it through stressful events

1. Strongly disagree
2. Disagree
3. Neither agree nor disagree
4. Agree
5. Strongly agree
6. Prefer not to say

It does not take me long to recover from a stressful event

1. Strongly disagree

For peer review only

2. Disagree
3. Neither agree nor disagree
4. Agree
5. Strongly agree
6. Prefer not to say

It is hard for me to snap back when something bad happens

1. Strongly disagree
2. Disagree
3. Neither agree nor disagree
4. Agree
5. Strongly agree
6. Prefer not to say

I usually come through difficult times with little trouble

1. Strongly disagree
2. Disagree
3. Neither agree nor disagree
4. Agree
5. Strongly agree
6. Prefer not to say

I tend to take a long time to get over setbacks in my life

1. Strongly disagree
2. Disagree
3. Neither agree nor disagree
4. Agree
5. Strongly agree
6. Prefer not to say

Reference: Smith, B. W., Dalen, J., Wiggins, K., Tooley, E., Christopher, P., & Bernard, J. (2008). The brief resilience scale: assessing the ability to bounce back. *International journal of behavioral medicine*, *15*(3), 194–200. https://doi.org/10.1080/10705500802222972

Rosenberg Self-Esteem Scale (RSE) (Rosenberg, 1965)

The RSE is a ten-item self-report measure used to assess global self-esteem in adults. This measure will be administered at baseline only.

On the whole, I am satisfied with myself

1. Strongly agree
2. Agree
3. Disagree
4. Strongly disagree
5. Prefer not to say

At times I think I am no good at all

1. Strongly agree

For peer review only

2. Agree
3. Disagree
4. Strongly disagree
5. Prefer not to say

I feel that I have a number of good qualities

1. Strongly agree
2. Agree
3. Disagree
4. Strongly disagree
5. Prefer not to say

I am able to do things as well as most other people

1. Strongly agree
2. Agree
3. Disagree
4. Strongly disagree
5. Prefer not to say

I feel that I do not have much to be proud of

1. Strongly agree
2. Agree
3. Disagree
4. Strongly disagree
5. Prefer not to say

This is a quality control question, **please select 'Strongly disagree'**

1. Strongly agree
2. Agree
3. Disagree
4. Strongly disagree
5. Prefer not to say

I certainly feel useless at times

1. Strongly agree
2. Agree
3. Disagree
4. Strongly disagree
5. Prefer not to say

I feel that I am a person of worth

1. Strongly agree
2. Agree
3. Disagree
4. Strongly disagree
5. Prefer not to say

I wish I could have more respect for myself

1. Strongly agree
2. Agree
3. Disagree
4. Strongly disagree

For peer review only

5. Prefer not to say

All in all, I am inclined to think that I am a failure

1. Strongly agree
2. Agree
3. Disagree
4. Strongly disagree
5. Prefer not to say

I take a positive attitude toward myself

1. Strongly agree
2. Agree
3. Disagree
4. Strongly disagree
5. Prefer not to say

Reference: Rosenberg, M. (1965). *Rosenberg Self-Esteem Scale (RSES)* [Database record]. APA PsycTests. https://doi.org/10.1037/t01038-000

For peer review only

Global Cognitive Styles Questionnaire (CSQ) (Pearson et al. 2013)

The CSQ is a measure utilised to assess a participant's negative cognitive style through four items. This measure will be administered at baseline only.

I avoid saying what I think for fear for being rejected

1. Very like me
2. Quite like me
3. Quite unlike me
4. Very unlike me
5. Prefer not to say

I always expect criticism

1. Very like me
2. Quite like me
3. Quite unlike me
4. Very unlike me
5. Prefer not to say

I feel that I don't want people to really know me

1. Very like me
2. Quite like me
3. Quite unlike me
4. Very unlike me
5. Prefer not to say

My value as a person depends on what others think of me

1. Very like me
2. Quite like me

3. Quite unlike me
4. Very unlike me
5. Prefer not to say

Reference: Pearson, R. M., Fernyhough, C., Bentall, R., Evans, J., Heron, J., Joinson, C., Stein, A. L., & Lewis, G. (2013). Association between maternal depressogenic cognitive style during pregnancy and offspring cognitive style 18 years later. The American journal

of psychiatry, 170(4), 434–441. https://doi.org/10.1176/appi.ajp.2012.12050673

The Perceived Stress Scale (Cohen et al., 1983)

The PSS is used to measure perceived stress in the last month and will be assessed at the 2-week questionnaire only.

In the last month, how often have you been upset because of something that happened unexpectedly?

1. Never
2. Almost never
3. Sometimes
4. Faily often
5. Very often

In the last month, how often have you felt that you were unable to control important things in your life?

1. Never
2. Almost never
3. Sometimes
4. Faily often
5. Very often

In the last month, how often have you felt nervous and "stressed"?

1. Never
2. Almost never
3. Sometimes
4. Faily often
5. Very often

In the last month, how often have you dealt successfully with irritating life hassles?

1. Never
2. Almost never
3. Sometimes
4. Faily often
5. Very often

For peer review only

In the last month, how often have you felt that you were effectively coping with important changes that were occurring in your life?

1. Never
2. Almost never
3. Sometimes
4. Faily often
5. Very often

In the last month, how often have you felt confident about your ability to handle your personal problems?

1. Never
2. Almost never
3. Sometimes
4. Faily often
5. Very often

In the last month, how often have you felt that things were going your way?

1. Never
2. Almost never
3. Sometimes
4. Faily often
5. Very often

In the last month, how often have you found that you could not cope with all the things that you had to do?

1. Never
2. Almost never
3. Sometimes
4. Faily often
5. Very often

In the last month, how often have you been able to control irritations in your life?

1. Never
2. Almost never
3. Sometimes
4. Faily often
5. Very often

In the last month, how often have you felt that you were on top of things?

1. Never

For peer review only

2. Almost never
3. Sometimes
4. Faily often
5. Very often

In the last month, how often have you been angered because of things that happened that were outside of your control?

1. Never
2. Almost never
3. Sometimes
4. Faily often
5. Very often

In the last month, how often have you found yourself thinking about things that you have to accomplish?

1. Never
2. Almost never
3. Sometimes
4. Faily often
5. Very often

In the last month, how often have you been able to control the way you spend your time?

1. Never
2. Almost never
3. Sometimes
4. Faily often
5. Very often

In the last month, how often have you felt difficulties were piling up so high that you could not overcome them?

1. Never
2. Almost never
3. Sometimes
4. Faily often
5. Very often

Reference: Cohen, S., Kamarck, T., & Mermelstein, R. (1983). Perceived Stress Scale [Database record]. APA PsycTests. https://doi.org/10.1037/t02889-000

For peer review only

Big Five Inventory – 15 Item Short Form (Lang et al. 2011)

The Big Five Inventory - 15 item Short Form is a shorter version of the traditional Big Five Inventory for assessing personality. This will be measured administered at the 4 week follow up only.

I am sometimes rude to others

1. Strongly disagree
2. Disagree a little
3. Neither agree nor disagree
4. Agree a little
5. Agree strongly

I am someone who does a thorough job

1. Strongly disagree
2. Disagree a little
3. Neither agree nor disagree
4. Agree a little
5. Agree strongly

I am someone who is talkative

1. Strongly disagree
2. Disagree a little
3. Neither agree nor disagree
4. Agree a little
5. Agree strongly

I am someone who worries a lot

1. Strongly disagree
2. Disagree a little
3. Neither agree nor disagree
4. Agree a little
5. Agree strongly

I am someone who is original, comes up with new ideas

1. Strongly disagree
2. Disagree a little
3. Neither agree nor disagree
4. Agree a little
5. Agree strongly

For peer review only

I am someone who has a forgiving nature

1. Strongly disagree
2. Disagree a little
3. Neither agree nor disagree
4. Agree a little
5. Agree strongly

I am someone who tends to be lazy

1. Strongly disagree
2. Disagree a little
3. Neither agree nor disagree
4. Agree a little
5. Agree strongly

I am someone who is outgoing, sociable

1. Strongly disagree
2. Disagree a little
3. Neither agree nor disagree
4. Agree a little
5. Agree strongly

I am someone who gets nervous easily

1. Strongly disagree
2. Disagree a little
3. Neither agree nor disagree
4. Agree a little
5. Agree strongly

I am someone who values artistic things

1. Strongly disagree
2. Disagree a little
3. Neither agree nor disagree
4. Agree a little
5. Agree strongly

I am someone who is considerate and kind to almost everyone

1. Strongly disagree
2. Disagree a little
3. Neither agree nor disagree
4. Agree a little

For peer review only

5. Agree strongly

I am someone who does things efficiently

1. Strongly disagree
2. Disagree a little
3. Neither agree nor disagree
4. Agree a little
5. Agree strongly

I am someone who is reserved

1. Strongly disagree
2. Disagree a little
3. Neither agree nor disagree
4. Agree a little
5. Agree strongly

I am someone who is relaxed and handles stress well

1. Strongly disagree
2. Disagree a little
3. Neither agree nor disagree
4. Agree a little
5. Agree strongly

I am someone who has an active imagination

1. Strongly disagree
2. Disagree a little
3. Neither agree nor disagree
4. Agree a little
5. Agree strongly

Reference: Lang, F. R., John, D., Lüdtke, O., Schupp, J., & Wagner, G. G. (2011). Short assessment of the Big Five: robust across survey methods except telephone interviewing. Behavior research methods, 43(2), 548–567. https://doi.org/10.3758/s13428-011-0066-z

Maslach Burnout Inventory: MBI-GS – (Maslach & Jackson, 1981)

The MBI-GS is a short form measure of the original Maslach Burnout Inventory and is a concise measure of burnout. It will be administered at the 6-week questionnaire only.

For peer review only

I feel emotionally drained from my work.

1. Never
2. A few times a year or less
3. Once a month or less
4. Once a week
5. A few times a week
6. Every day

I feel used up at the end of the workday.

1. Never
2. A few times a year or less
3. Once a month or less
4. Once a week
5. A few times a week
6. Every day

I feel tired when I get up in the morning and have to face another day on the job.

1. Never
2. A few times a year or less
3. Once a month or less
4. Once a week
5. A few times a week
6. Every day

Working all day is really a strain for me.

1. Never
2. A few times a year or less
3. Once a month or less
4. Once a week
5. A few times a week
6. Every day

I can effectively solve the problems that arise in my work.

1. Never
2. A few times a year or less
3. Once a month or less
4. Once a week
5. A few times a week
6. Every day

I feel burned out from my work.

1. Never

For peer review only

2. A few times a year or less
3. Once a month or less
4. Once a week
5. A few times a week
6. Every day

I feel I am making an effective contribution to what this organization does.

1. Never
2. A few times a year or less
3. Once a month or less
4. Once a week
5. A few times a week
6. Every day

I have become less interested in my work since I started this job.

1. Never
2. A few times a year or less
3. Once a month or less
4. Once a week
5. A few times a week
6. Every day

I have become less enthusiastic about my work.

1. Never
2. A few times a year or less
3. Once a month or less
4. Once a week
5. A few times a week
6. Every day

In my opinion, I am good at my job.

1. Never
2. A few times a year or less
3. Once a month or less
4. Once a week
5. A few times a week
6. Every day

I feel exhilarated when I accomplish something at work.

1. Never
2. A few times a year or less
3. Once a month or less
4. Once a week

For peer review only

5. A few times a week
6. Every day

I have accomplished many worthwhile things in this job.

1. Never
2. A few times a year or less
3. Once a month or less
4. Once a week
5. A few times a week
6. Every day

I just want to do my job and not be bothered.

1. Never
2. A few times a year or less
3. Once a month or less
4. Once a week
5. A few times a week
6. Every day

I have become more cynical about whether my work contributes anything.

1. Never
2. A few times a year or less
3. Once a month or less
4. Once a week
5. A few times a week
6. Every day

I doubt the significance of my work.

1. Never
2. A few times a year or less
3. Once a month or less
4. Once a week
5. A few times a week
6. Every day

At my work, I feel confident that I am effective at getting things done.

1. Never
2. A few times a year or less
3. Once a month or less
4. Once a week
5. A few times a week
6. Every day

For peer review only

Maslach, C., & Jackson, S. E. (1981). The Measurement of Experienced Burnout. *Journal of Occupational Behaviour*, *2*(2), 99–113. https://doi.org/10.1002/job.4030020205

EMA Items (Non-Validated Measures from Surveys and Questionnaires)

Current Location and Activities

This two-item measure assess where participants have spent the most time between prompts and to report on their main activities during this time. Items assessing location and current activities may give insight into how a participant's environment or activity may be related to their reported mood and other factors reported via daily EMA measures. These items are included in all 3 brief daily surveys.

In the last 3 hours, **where have you spent the most time?**

1. At home
2. At a friend's place
3. At my romantic partner's place
4. At my family's place
5. At work / place of education
6. In a car / on public transport
7. Other indoor location
8. Outdoor location (urban/city)
9. Outdoor location (rural/in nature)
10. At a hotel or Airbnb

In the last 3 hours, **what have you mainly been doing?** Select all that apply:

1. Social activities (in person)
2. Online social activities
3. Physical activity/leisure (e.g., walking, exercising)
4. Mentally stimulating activities (e.g., reading a book, puzzles, board game)
5. Passive leisure (e.g., watching TV, scrolling social media)
6. Studying / working
7. Chores (e.g., groceries, cooking, cleaning)
8. Childcare
9. Travelling / on my way somewhere
10. Eating
11. Sleeping
12. Other

Working patterns

To gather data on non-standard working patterns and other reasons for participants systematically missing surveys and questionnaires, they will be asked the following question:

For peer review only

It's important that you answer as many surveys and questionnaires as possible throughout the study, however we understand that work and other commitments might stop you from doing so. We'd still love you to take part, even if you can't respond to every notification.

Please select one of the options below to let us know about any commitments that will regularly stop you from responding to notifications in the next 6 weeks:

1. I work shifts or have a non-typical working pattern (e.g., work starts before 7am or after 2pm, hours rotate, or regularly includes hours outside of 7am to 6pm)
2. I have caring responsibilities
3. I am unable to access my phone during working hours
4. N/A – I can normally respond to notifications at any time.

Stress

Stress will be assessed using the following single item assessed with a sliding scale, included in all 3 brief daily surveys.

In the last 3 hours, **have you been feeling stressed?**

0. Not at all

to

100. Extremely

Prefer not to say

Positive and Negative Affect

Stress will be assessed using the following two items assessed with a sliding scale for the first one and a selection of options for the second, included in all 3 brief daily surveys.

How positive or negative do you feel right now?

0. Positive

to

10. Negative

Prefer not to say

For peer review only

Reflect on what you were thinking about prior to answering this survey

1. I was conscious of my inner feelings.

2. I was self-conscious about the way I look or was concerned about the way I present myself.

3. I was conscious of what is going on around me.

4. I was reflective about my life.

5. I was concerned about what other people think of me

6. I was aware of my innermost thoughts.

7. None of the above

Prefer not to say

Exercise

Participants will be asked a variety of items on exercise across the surveys and questionnaires. The questions for the daily surveys, were adapted from a question in Hirvensalo et al. (2000) and response options from Bruening et al. (2016).

Additionally, at baseline and for every two-week follow-up, participants will be asked three further items on exercise and time spent outdoors.

[Following Question is asked in brief daily surveys]

In the last 3 hours, what has been your highest level of physical activity?

0. No movement (e.g., sitting at a desk, on the sofa, travelling by car or public transport)
1. Moved only in connection with necessary tasks (e.g., household chores)
2. Gentle exercise or leisurely activities requiring little effort (e.g., gentle walking, gardening, yoga)
3. Moderate exercise - increased your heart rate but not exhausting e.g., brisk walking, dancing, cycling, weights)
4. Strenuous exercise causing sweating / heavy breathing (e.g., running, football, fast swimming, HIIT)

[Following Questions are asked in bi-weekly questionnaires]

In the past 2 weeks, how many times have you done any strenuous exercise to the point of sweating and heavy breathing (e.g., running, HIIT, football, fast swimming)?

1. None

For peer review only

2. 1 – 2 times
3. 3 – 4 times
4. 5 – 7 times
5. 8 – 10 times
6. 11 - 13 times
7. 14 or more times

In the past 2 weeks, how many times have you done any moderate exercise that increased your heart rate but was not exhausting (e.g., walking fast, hiking, lifting weights, dancing)?

1) None
2) 1 – 2 times
3) 3 – 4 times
4) 5 – 7 times
5) 8 – 10 times
6) 11 - 13 times
7) 14 or more times

In the past 2 weeks, how many times have you done any mild exercise that required little effort (e.g., gentle walking, yoga, bowling)

1) None
2) 1 – 2 times
3) 3 – 4 times
4) 5 – 7 times
5) 8 – 10 times
6) 11- 13 times
7) 14 or more times

Thinking about the past 2 weeks, how many minutes did you spend outdoors on a typical day?

1) 0-15 minutes
2) 16-60 minutes
3) 61-120 minutes (1-2 hours)
4) 121-180 minutes (2-3 hours)
5) 181+ minutes (3+ hours)

Sleep

Participants will be asked to rate the quality of their sleep using items from the Pittsburgh Sleep Quality Index (PSQI) (Buysse et al. 1989). The sleep quality item will be included in all 3 daily surveys to reduce possible data loss in participants with atypical sleep patterns (e.g., shift-workers). However, the sleep and wake time questions will only be asked once per day.

For peer review only

Further, during baseline and bi-weekly questionnaires participants will be asked again to rate the overall quality of their sleep. If a participant indicates that their average sleep has been of bad quality (answers ≥4) they will be prompted to answer a further question about how their sleep affects their daily life.

[Following Question is asked in brief daily surveys]

How would you rate the quality of your sleep last night?

0. Very poor
1. Poor
2. Neutral
3. Good
4. Very good

What time did you fall asleep last night ________ [answer in hours/minutes]

What time did you wake up today ____________ [answer in hours/minutes]

[Following Questions are asked in bi-weekly questionnaires]

In the past 2 weeks, how would you rate the quality of your sleep on average?

1. Very good
2. Fairly good
3. Average
4. Fairly poor
5. Very poor

In the past 2 weeks, have your sleep difficulties interfered with your daily life? [only for participants who answer 4 or 5 to preceding question]

1. Not at all
2. A little bit
3. Moderately
4. Quite a bit
5. Extremely

Diet

To assess diet and diet quality in participants we will administer two items. The first will ask participants if they have eaten since the last prompt. Those who indicate that they have eaten,

For peer review only

they will be directed to the second item asking about the type of food they have eaten (e.g., processed or homemade food). These items are adapted from Berge et al. (2023) and will be included in all 3 daily surveys.

Have you eaten in the last 3 hours? Select all that apply:

1. Yes, I have eaten a main meal (breakfast, lunch, or dinner)
2. Yes, I have eaten a snack
3. No, I have not eaten since the last prompt

Which best describes the type of food you ate on this occasion? Select all that apply: **[only for participants who answer 1 or 2 to preceding question]**

1. Fast food / take-out (consumed at fast food restaurant, home, or other location)
2. Other food eaten outside the home (e.g., a restaurant, café)
3. Pre-prepared or processed foods (e.g., frozen, oven-cook or microwaveable meals, breakfast cereals, crisps, biscuits, etc.)
4. Homemade / freshly prepared food (e.g., made from fresh fruits or vegetables, meat, fish, dairy, grains, pulses)

Menstruation

A single item will enquire as to whether participants have menstruated in the last 24 hours. There will be a '*prefer not to say*' option for those who do not wish to answer.

Did you have your period **in the last 24 hours?**

1. Yes
2. No

Hormonal Contraception/Medication Use and Gynaecological or Reproductive Health Conditions

Three questions will enquire as to whether participants are currently taking any hormonal contraceptives or medications at baseline and every 2-weekly questionnaire. A single question about experience of gynaecological or reproductive health conditions will be included in the baseline questionnaire.

Are you currently using any hormonal contraceptives or medications? This can be for any reason (e.g., birth control, acne, pain/cramps)

1. Yes

For peer review only

2. N/A
3. I don't currently take any hormonal contraception or medication
4. I've stopped taking hormonal contraceptives or medications within the last three months.
5. I use a non-hormonal copper coil/IUD

Please select the type of hormonal contraceptives or medications you are currently taking (select all that apply)

[Only for participants who answer 'Yes' to previous question]

1. Combined pill (e.g., Microgynon, Rigevidon, Ovranette)
2. Progestogen only pill ("mini-pill" e.g., Cerazette, Cerelle, Norgeston)
3. Contraceptive injection (e.g., Depo-Provera, Sayana Press)
4. Contraceptive patches (e.g., Evra Patches)
5. Vaginal ring (e.g., NuvaRing)
6. Hormonal coil/IUS (Mirena, Levosert, Benilexa, Kyleena, Jaydess)
7. Contraceptive implant (e.g., Nexplanon)
8. Other (please give details): [Prompts free text box]

How long have you been taking any type of hormonal contraceptive for?

[Only for participants who answer 'Yes' to taking hormonal contraception/medication]

1. 0 – 3 months
2. 3 – 6 months
3. 6 – 12 months
4. 1 year – 2 years
5. 2 years plus

Have you ever had any of the following gynaecological or reproductive health conditions? Select all that apply:

[baseline questionnaire only]

1. N/A
2. No
3. Endometriosis
4. Polycystic ovary syndrome (PCOS)
5. Fibroid(s)
6. Ovarian cyst
7. Pelvic inflammatory disease (PID)
8. Other (please give details) [Prompts free text box]

For peer review only

Substance Use (Smoking and Alcohol)

We will assess substance use with a single item in all 3 brief daily surveys.

In the last 3 hours, have you done any of the following? Select all that apply:

1. Smoked
2. Vaped
3. Drank alcohol
4. None of the above

Social Connectedness

Social connectedness will be assessed with three items asking participants how enjoyable they have found different types of social contact, including in-person and online. Items are adapted from Fried et al. (2023). These will be administered in all 3 brief daily surveys.

In the last 3 hours, **how enjoyable was spending time with other people in person?**

1. Not at all
2. Somewhat
3. Moderately
4. Very
5. Extremely
6. I haven't had any in-person contact in the last 3 hours

In the last 3 hours, **how enjoyable was spending time online using social media sites or apps such as Instagram, TikTok or X?**

1. Not at all
2. Somewhat
3. Moderately
4. Very
5. Extremely
6. I haven't spent any time using social media in the last 3 hours

In the last 3 hours, **how enjoyable was spending time with other people online through messaging sites (e.g., WhatsApp), video calls, or by phone?**

1. Not at all
2. Somewhat
3. Moderately
4. Very
5. Extremely
6. I haven't had any online social interactions in the last 3 hours

For peer review only

Pain

Participants will be asked about their experience of pain in the baseline questionnaire and at every two-week follow-up questionnaire. Those who report experiencing any pain will be asked a follow up question about the impact this has had on their life or work activities.

In the past 2 weeks, how often have you had any pain?

1. Never
2. Some days
3. Most days
4. Every day
5. Prefer not to say

In the past 2 weeks, how often did pain limit your life or work activities? [only for participants who answer 2 -4 to preceding question]

1. Never
2. Some days
3. Most days
4. Every day
5. Prefer not to say

Recent Life Events

At baseline and at every two-week follow-up questionnaire, participants will be asked if they have experienced a range of recent life events. By assessing life events over the previous two weeks the questionnaire shall elucidate potential positive or negative circumstances which may impact depressive symptoms and mood.

In the past 2 weeks, have any of the following happened to you [tick all that apply]?

1. You entered a new romantic relationship
2. You had a mild illness (e.g., cough, cold, flu, fever)
3. You became seriously ill
4. You accomplished a personal achievement
5. You got into serious financial problems
6. You or your partner became pregnant
7. You started a new job
8. A romantic relationship ended
9. You took a holiday
10. A friend or family member died
11. You or your partner had a baby
12. You had a big argument with a friend or family member
13. You lost your job
14. No significant life events in the past 2 weeks
15. Other (please give details):

For peer review only

16. Prefer not to say

Treatment and Medication

Participants will be asked if they have received any of the following treatments for anxiety or depression: antidepressant medication, cognitive behavioural therapy, any other form of psychological therapy, or any self-help guidance. Participants will be asked to provide this information at baseline and at every two-week questionnaire following this.

In the past 2 weeks, have you taken or received any of the following? Select all that apply:

1. Antidepressant medication for anxiety or depression
2. Cognitive behavioural therapy for anxiety or depression
3. Other forms of psychological therapy for anxiety or depression
4. Any self-help guidance for anxiety or depression
5. None of the above
6. Prefer not to say

Current and Previous Mental Health

Participants will be asked about current and previous mental health diagnoses. Participants will be asked about this at baseline only.

Have you ever been diagnosed with a mental health condition by a health-care professional (e.g., doctor, psychiatrist, therapist, nurse, healthcare professional with specialist training)?

1. Yes, I have a current mental health diagnosis.
2. Yes, I have had a past mental health diagnosis.
3. Yes, I have **both a previous and current** mental health diagnosis.
4. No, I have never received a mental health diagnosis.
5. I'm not sure.
6. Prefer not to say

Please select which mental health diagnosis [select all that apply]

[Only for participants who answer 'Yes' to even been diagnosed with a mental health condition]

1. Depression

2. Anxiety

3. Phobia

4. Bi-polar disorder

For peer review only

4. Body dysmorphia

5. Premenstrual dysphoric disorder

6. Post Traumatic Stress Disorder

7. Obsessive Compulsive Disorder

8. An eating disorder

9. Psychosis

10. Schizophrenia or schizoaffective disorder

11. A personality disorder

12. Other (not listed)

Questionnaire and Study Feedback

Following the completion of the EMA study at six weeks participants will be asked to provide feedback on their experience of using the m-Path application and of participation in the study in general.

Helplines

The helplines below will be shown at the end of every daily survey and at the end of every questionnaire. Additionally, if someone answers the PHQ-9 with a positive score of ≥1 they will immediately be shown the helplines below before being able to progress with the survey.

- **SAMARITANS** - Emotional support for everyone
  Website: https://www.samaritans.org/scotland/ | *Tel:* 116 123 (24 hours)
- **MIND** - Advice and support for anyone with a mental health problem
  Website: https://www.mind.org.uk | *Tel:* 0300 123 3393 Text: 86463
- **SHOUT** - Support around self-harm
  Website: https://giveusashout.org/get-help/issues/help-self-harm/ | *Text:* 85258
- **NHS 111** - If you need medical help right now, 111 online can tell you what to do next | *Website:* https://111.nhs.uk.

For peer review only

BMJ Open

Mood in the Moment: a study protocol for embedding ecological momentary assessments into established longitudinal cohorts to examine depression in real-time

Journal:	*BMJ Open*
Manuscript ID	bmjopen-2026-122195.R1
Article Type:	Protocol
Date Submitted by the Author:	03-Jun-2026
Complete List of Authors:	Kwong, Alex S F; The University of Edinburgh, Division of Psychiatry; University of Bristol, Population Health Sciences; University of Bristol, MRC Integrative Epidemiology Unit<br>Moody, Sarah ; The University of Edinburgh, Division of Psychiatry<br>Taylor, Adele; The University of Edinburgh, Division of Psychiatry<br>Lockhart, Celestine ; King's College London, Social, Genetic and Developmental Psychiatry<br>Ogden, Rachel ; King's College London, Social, Genetic and Developmental Psychiatry<br>Leal, Ray; King's College London, Social, Genetic and Developmental Psychiatry<br>Mcmillan, Andrew ; King's College London, Social, Genetic and Developmental Psychiatry<br>Harvey, Jessica ; University of Bristol, Population Health Sciences<br>Brierley, Elizabeth ; University of Bristol, Population Health Sciences<br>Simmons, Anne ; University of Bristol, Population Health Sciences<br>Matthews, Sarah ; University of Bristol, Population Health Sciences<br>Hobbs, Richard ; University of Bristol, Population Health Sciences<br>Eley, Thalia; King's College London, Social, Genetic, and Developmental Psychiatry Centre; National Institute for Health and Care Research (NIHR) Maudsley Biomedical Research Centre<br>Jacobson, Nicholas ; Dartmouth College, Center for Technology and Behavioral Health, Geisel School of Medicine<br>Murray, Aja; The University of Edinburgh, Department of Psychology
**Primary Subject Heading**:	Mental health
Secondary Subject Heading:	Epidemiology, Public health
Keywords:	Depression & mood disorders < PSYCHIATRY, Longitudinal studies, Child & adolescent psychiatry < PSYCHIATRY, Adult psychiatry < PSYCHIATRY, STATISTICS & RESEARCH METHODS

Mood in the Moment: a study protocol for embedding ecological momentary assessments into established longitudinal cohorts to examine depression in real-time

Alex S. F. Kwong[1,2,3*], Sarah Moody[1^], Adele Taylor[1^], Celestine Lockhart[4], Rachel Ogden[4], Ray Leal[4], Andrew McMillan[4], Jessica Harvey[2], Elizabeth Brierley[2], Sarah Matthews[2], Richard Hobbs[2], Anne Simmons[2], Thalia C. Eley[4,5], Nicholas C. Jacobson[6], Aja L. Murray[7]

[1]Division of Psychiatry, Centre for Clinical Brain Sciences, University of Edinburgh, UK

[2]Population Health Sciences, Bristol Medical School, University of Bristol, UK

[3]MRC Integrative Epidemiology Unit, University of Bristol, UK

[4]Social, Genetic and Developmental Psychiatry, King's College London

[5]National Institute for Health and Care Research (NIHR) Maudsley Biomedical Research Centre, South London and Maudsley NHS Foundation Trust, London, UK

[6]Center for Technology and Behavioral Health, Geisel School of Medicine, Dartmouth College

[7]Department of Psychology, University of Edinburgh, Edinburgh, UK

*Corresponding author: Division of Psychiatry, University of Edinburgh, 49 Little France Crescent, BioQuarter, Edinburgh, EH16 4TJ; akwong@ed.ac.uk

^joint second authors

Word Count: 3839 (excluding abstract and references)

For peer review only

Mood in the moment: an EMA study on depression

Abstract

Introduction

Depression is a major global health challenge, with onset commonly occurring in youth. There is an urgent need to better understand the epidemiology of depression to facilitate better interventions and preventions that are person and timing specific. Long-term depression trajectories and Ecological Momentary Assessment (EMA) can help address this need. Embedding EMA designs within established longitudinal cohorts offers a uniquely powerful approach to examine depression in both long-and-short term settings and to identify distal and proximal modifiable risk factors for depression.

Methods and analyses

Our study will include approximately 450 participants from the Twins Early Development Study (TEDS) and 250 participants from the Avon Longitudinal Study of Parents and Children (ALSPAC) who have depression trajectories data previously collected between late childhood and early adulthood. Participants will be recruited from four different depression trajectories in each cohort, based upon participants prior symptoms. They will undertake EMA surveys of depression, sleep, physical activity, substance use, diet, recent activities and social interactions, three times a day for six weeks, with more detailed questionnaires at baseline, 2, 4 and 6 weeks. We will use descriptive analysis, mixed effects models and dynamic structural equation modelling to examine how depression occurs over this time, how potentially modifiable factors affect depression and vice versa and how these effects vary by different life-course trajectories. This study is designed to identify people at heightened risk of depression and/or identify modifiable targets that could inform more effective prevention and intervention strategies.

Ethics and dissemination

Favourable ethical opinions were given by the Edinburgh Medical School Research Ethics Committee (REC References: 25-EMREC-001 and 25-EMREC-030) and the ALSPAC Law and Ethics Committee (Ref: 0027 B4792). The results will be disseminated through journal publications, conferences and seminar presentations and to relevant stakeholders, such as those with a history of depression, policy makers and clinicians.

For peer review only

Strengths and limitations of the study

- This study will embed ecological momentary assessment (EMA) within two established longitudinal cohort studies.
- Around 700 participants will complete brief EMA assessments up to three time a day, for six weeks, with additional baseline and fortnightly questionnaires.
- A range of measures will be used during the EMA study, allowing for the identification of important modifiable factors related to depression in real-time, for specific groups of individuals with different depression histories.
- In ALSPAC, a subset of participants will also wear smartwatches to passively collect data on sleep, activity and heart rate.
- Attrition is likely to play a role in the EMA data collection, especially for participants with a history of depression.

For peer review only

Introduction

Depression is a global health challenge, affecting over an estimated 330 million people worldwide [1], and predicted to be the leading cause of disability by 2030 [2]. Young people are at particular risk of depression [3], with emerging evidence that depression is worsening within this population [4, 5]. Depression during this period is associated with wide-ranging and long-lasting economic, social, and physiological consequences [6-9]. Evidence suggests that 50% of people who have ever had depression, had an onset before the age of 25 [10], and almost 50% of those who develop depression between 18 and 40 will have a subsequent episode [11]. This makes it crucial to prevent or limit depression during earlier life to mitigate later effects. Understanding what modifiable factors and mechanisms influence the course of depression is therefore of considerable clinical, economic and societal importance. Such research could ultimately help identify people at heightened risk of worsening depression and/or identify modifiable targets that could inform more effective prevention and intervention strategies.

Longitudinal studies that track the same people over time are critical to addressing this line of research. Such studies can estimate depression trajectories across development and highlight how depression develops, persists, and remits across the life course, as well as what trajectories are specific for different aetiologies and outcomes (for reviews see [12-14]). Importantly, they have also identified key risk and protective factors such as sex and pubertal timing [15, 16], genetic vulnerability [17, 18], early adversity [7] and the family environment [19, 20] that likely shape depression over time. Whilst such research is important, these studies typically assess depression at yearly intervals or longer. As such, there is a current paucity for what depression looks like in between these assessments, and how depression and its correlates may fluctuate and drive one another in the short-term, from day-to-day, or even moment to moment settings. A greater understanding of more granular short-term manifestation could inform treatment provision and illuminate the perpetuating or proximal; mechanisms that drive depression in day-to-day life.

Ecological momentary assessment (EMA) has emerged as a powerful tool for addressing this limitation [21-23]. EMA designs can collect data multiple times a day whilst participants go about

For peer review only

their daily routines, enabling researchers to examine depression as it unfolds in naturalistic settings and to identify how specific contextual factors can shape mood and vice versa, all in close to real time [24, 25]. EMA designs can be paired with passive data collection (i.e., through smartwatches) to provide objective measures of factors such as sleep, exercise or heart rate, and their relation to daily mood [26] in a continuous, non-invasive manner. EMA is particularly well suited to capturing the dynamic, person and context-dependent nature of depression, providing the opportunity to measure short-term depression trajectories over a given period. It can also capture symptom variability, the occurrence of correlated factors and perhaps most importantly, reactivity to daily stressors and life events in a way that retrospective designs cannot reliably detect. A growing body of EMA research has demonstrated that short-term fluctuations in depression are both clinically meaningful and informative [27]. For example, EMA designs have shown that depression is not stable, even across a single day [28]. Furthermore, momentary factors such as disrupted sleep [28, 29], reduced physical activity [28, 30, 31] and worse social interactions are believed to influence depressive states in the short term [24, 32, 33]. However, existing EMA studies of depression have predominantly been conducted in small samples (often clinical populations or student cohorts), without access to broader phenotypic or prospectively ascertained life-course data. This limits their capacity to examine how short-term depressive dynamics fit within the broader context of development and the life-course.

To date, long-term trajectory research and short-term intensive data collection have not been conducted in depression research, with only a handful of studies embedding broader EMA designs into longitudinal studies, such as in ADHD research [34]. This represents a significant missed opportunity to combine detailed data on depression in the moment and short-term depression trajectories, alongside detailed longer-term depression trajectories derived from routinely collected data. Short-term depression symptoms and the modifiable factors that drive them such as sleep, exercise, diet, substance use, and social interactions could illuminate proximal mechanisms that underpin longer-term depression trajectories - thus highlighting opportunities for more tailored and targeted interventions and preventions that are timing and person specific. Embedding EMA within established longitudinal cohorts, whose participants have well-characterised life-course depression

For peer review only

trajectories, offers a uniquely powerful approach to address this gap. By selecting participants based upon previously characterised depression trajectories, it is possible to examine the real-time epidemiology of depression and how it varies across individuals with different histories of depressive experience. This can also be combined with data collected across development such as psycho-social factors, family history and genetic risk, and identify the momentary risk and protective factors that are most relevant within different trajectories.

In this study, we will use an EMA design embedded within two established longitudinal studies: the Twins Early Development Study (TEDS) and the Avon Longitudinal Study of Parents and Children (ALSPAC) to study depression in both the long- and short-term. Both studies have extensive life-course data on depression and its determinants, allowing us to derive clinically meaningful and robust depression trajectories from childhood to early/emerging adulthood. The nature of this study will allow us to examine how short-term depressive dynamics occur and vary within the context of longer-term depression trajectories, and how momentary risk and protective factors such as sleep, physical activity, diet, substance use, social interactions and stress contribute to these fluctuations in real time. Furthermore, in a subsample of ALSPAC participants, concurrent passive data collection via wearable smartwatch devices will be collected to enrich this picture by providing objective physiological measures of sleep, physical activity, and heart rate, complementing and contextualising the self-reported EMA data [35, 36]. Our core research questions are:

- RQ1) How does depression occur and vary in the short-term - from day-to-day or even moment-to-moment settings in people with different prior life-course depression trajectories?
- RQ2) Which momentary risk and protective factors such as sleep, stress, physical activity, diet, substance use, and social interactions are most strongly associated with short-term fluctuations and symptoms in depression in daily life, and how do these vary by different depression trajectories?
- RQ3) How do short-term depression dynamics relate to longer-term depression trajectories previously characterised in the cohort, and do they mediate or moderate the associations between established risk factors and longer-term outcomes?

For peer review only

Exploratory analyses may be used to address a range of additional questions, including examination of individual differences in the links between momentary risk factors and depression, using machine learning to predict greater levels of depression and to identify further potential targets for personalised and timely intervention. For more ambitious questions like RQ3, we will pre-register analysis on the open science framework.

For peer review only

Mood in the moment: an EMA study on depression

Methods and analysis

Existing study participants

Participants will be drawn from two existing longitudinal cohort studies: The Twins Early Development Study (TEDS) and the Avon Longitudinal Study of Parents and Children. In TEDS, the original sample comprised of 13,759 families with newborn twins, who participated in the first assessment wave at 18 months. Families were recruited from the Office of National Statistics' national register of twins born in England and Wales between 1994 and 1996 [37]. In ALSPAC, pregnant women residing in Avon, UK with expected delivery between 1st April 1991 and 31st December 1992 were invited to take part in the study. A total of 14,901 children alive at 1 year of age participated in ALSPAC [38-40]. Further cohort information is provided in the supplementary materials. Overall ethical approval for TEDS was granted by the King's College London Research Ethics Committee (References: PNM/09/10–104, HR/DP-20/2122060, and RESCM-24/25-22060), and consent was given by participants at each wave. In ALSPAC, overall ethical approval for the study was obtained from the ALSPAC Ethics and Law Committee and the Local Research Ethics Committees. Informed consent for the use of existing data collected was obtained from participants following the recommendations of the ALSPAC Ethics and Law Committee at the time. Participants can contact the study team at any time to retrospectively withdraw consent for their data to be used. Study participation is voluntary and during all data collection sweeps, information was provided on the intended use of data. In TEDS, the study website contains details of all data that is available: https://datadictionary.teds.ac.uk/home.htm. In ALSPAC, the study website contains details of all data that is available through a fully searchable data dictionary and variable search tool and reference the following webpage: http://www.bristol.ac.uk/alspac/researchers/our-data/.

EMA participants

The present study will include approximately 450 participants from TEDS and 250 participants from ALSPAC who have depression trajectories data previously collected between late childhood and early adulthood. We will only recruit participants who have responded to the most recent questionnaire on

depression within each study (both assessed in 2025) to ensure that participant belong to the most up to date depression trajectories. Participants in the EMA study will have a mean age of 29.5 years in TEDS and 32.6 years in ALSPAC. All participants will be required to have completed a minimum of two depression assessments (one in childhood/adolescence and another in late adolescence/early adulthood) to be included in the initial trajectory analysis in which to recruit from. Based upon recent questionnaire data collected in both cohorts, we have a maximum sample of 3275 participants in TEDS and 3866 participants in ALSPAC from which to recruit participants into the EMA section of the study. Based upon previous research [7, 41], we estimated four similar trajectories per cohort (a stable low trajectory, an adolescent-onset trajectory, an adolescent-limited trajectory and a persistent trajectory), see Figure 1. We will recruit equally from each trajectory group to ensure a matched case-control analysis, randomising the order in which we invite participants to join the EMA study. In ALSPAC, ~100 participants will be given Withings smartwatches to passively collect data on sleep, activity and heart rate throughout the study. Figure 2 shows a graphical representation of our study.

Inclusion criteria

The inclusion criteria for this study require participants to be active and consenting participants of the TEDS and ALSPAC studies and have previously measured data on depression (i.e., a minimum of two depression assessments [one in childhood/adolescence and another in late adolescence/early adulthood]) and have completed the most recent questionnaire on mental health. Eligible participants will be invited to join the study if they meet these criteria and will be invited by the TEDS and ALSPAC teams directly through existing contact information held by the studies. In TEDS, only participated who have consented to be contacted about future sub-studies will be invited to take part. Participants will be required to have a sufficient understanding of written and spoken English language to understand study information and instructions, provide informed consent, and participate in the text-based EMA surveys to be completed via smartphone application. Participants will also be required to own a smartphone device with access to either the app store (Apple iPhones) or Google Play Store (Android phones) to download the smartphone application m-Path to allow participation in the study for a period of six consecutive weeks. In ALSPAC, participants must be willing and able to

For peer review only

download the Withings smartwatch application and wear a Withings smartwatch for a period of six weeks (if consented to this aspect of the study). Across both studies, participants must be available to participate in the study for a consecutive six-week period without significant breaks while the data collection is live.

Patient and public involvement

Individuals with and without depression have contributed to the design of this study. We first consulted people with lived experience of depression as part of an ongoing user-led citizen science project called ‘The Depression Detectives’. In their capacity as advisors, they saw value in identifying patterns of depression early through smartphones and to identify key factors that could prevent depression from getting worse and leading to long-term problems. Next, a group of adolescents recruited as part of a patient and public lived experience group determined feasibility of the study, recommending the types of factors to include and duration of the study. Finally, active participants from the ALSPAC study provided guidance on the duration of the study, measures used and amount of reimbursement that would be suitable for the study.

Data collection

Data collection began in September 2025 and is expected to complete by August 2026. Analysis will be staggered across this period to avoid seasonality effects. Figure 2 shows a graphical representation of our study where participants will be recruited based upon their existing depression trajectories. Participants will then be invited to join this EMA study and provide consent as described above.

Participants who give consent and begin the study will be sent instructions on how to download the ‘M-Path’ EMA smartphone application. In ALSPAC, ~100 random (equal across trajectories) participants will be recruited into the smartwatch element of the study will also be sent instructions on how to download the ‘Withings’ smartphone application. Each participant will be sent their own unique and pseudo-anonymised login that only the respective study teams can use to identify participants.

For peer review only

Participants who successfully join the study will first complete a baseline questionnaire and have seven days to complete this before they are removed from the study. Completion of this baseline questionnaire will initiate EMA surveys starting the next morning. EMA surveys will occur 3 times a day (randomly in the morning [8am to 9am], afternoon [1pm to 2pm] and evening [6pm-7pm]) for six weeks (for a maximum of 126 surveys), all through the 'M-Path" smartphone application. These EMA surveys are designed to be completed in under 3 minutes, and each daily survey is available for 3 hours from the initial prompt. Reminders were sent after 60 and 120 minutes. Slightly longer questionnaires (taking approximately 5 minutes to complete) are also available to complete at the two- and four-week intervals. A final end-of-study questionnaire is then available to complete at the end of the study at week six. Participants will be reimbursed £1 for every EMA survey they complete, and £2 for each questionnaire they complete (baseline, two-week, four-week and end of study questionnaires). We have also implemented a bonus structure for the EMA surveys. If participants complete 50% of the EMA surveys (63 surveys), they will receive a further £5. Completing 60%, 70%, 80%, 90% or 100% results in a further £10, £15, £20, £25 or £30 bonus, respectively. Therefore, a participant completing 50% of the EMA surveys and 2 longer questionnaires would be reimbursed £72 (£63 for the 63 EMA surveys, the £5 bonus for 50% completed EMA surveys and £4 for the two longer questionnaires). A participant completing 100% of the EMA surveys and all four longer questionnaires would be reimbursed the maximum £164 (£126 for the 126 EMA surveys, the £30 bonus for 100% completed EMA surveys and £8 for the two longer questionnaires). ALSPAC participants with the smartwatches will receive an additional £10 reimbursement for taking part in smartwatch component of the study. Reimbursement will be given in the form of Love2Shop vouchers, administered by the respective study teams.

Measures

A full list of measures that will be assessed throughout the study and their schedules are presented in Table 1. A full description of the measures is detailed in the online supplementary material.

Statistical analysis

For peer review only

Data will be analysed using descriptive and advanced intensive longitudinal data analysis techniques, including descriptive analysis of each item [22], mixed effects models [28], and dynamic structural equation modelling (DSEM) [42]. Using these techniques will allow us to examine group and participant-level effects and derive individual-level variability of depressive symptoms and their associated risk and protective factors as measured across the EMA period. For example, we can derive indices of depressive dynamics including: the average total or specific depressive symptoms experienced across the study period, the degree of fluctuation in depression from one assessment to the next (symptom variability), the extent to which prior depression states predict subsequent ones, and the strength of the association between specific daily experiences (i.e., previous sleep, a period of social isolation) and subsequent momentary depressive mood. These indices can then be examined in relation to longer-term depression trajectory membership as previously characterised in each study, enabling investigation of whether individuals with different depression histories show systematically different patterns of short-term depressive dynamics. For smartwatch participants, these within-person dynamics will also be examined alongside passively collected physiological data from wearable devices, including objective sleep metrics, step count, and heart rate, to assess the contribution of passively collected data to real-time depressive fluctuations.

Sample size justification

Our sample size of approximately 450 participants in TEDS and approximately 250 participants in ALSPAC is based upon previous research comparing long term EMA data collection in case-control designs [42]. Power calculations for this particular EMA design (recruited from four different trajectories) are not available in current packages. However, simulation data indicated that ~150 participants per trajectory, completing ~50% of their measures would give 80% power to detect associations between depression and modifiable risk factors with small to moderate effect sizes (~0.2-0.3), within each trajectory.

Ethics and dissemination

For peer review only

Ethical approval to conduct this research in both TEDS and ALSPAC was given favourable opinions by the Edinburgh Medical School Research Ethics Committee (REC References: 25-EMREC-001 and 25-EMREC-030) and the ALSPAC Law and Ethics Committee (Ref: 0027 B4792). The results will be disseminated through journal publications, conferences and seminar presentations and to relevant stakeholders, such as those with a history of depression, policy maker and clinicians.

For peer review only

Discussion

This protocol outlines the design for a unique EMA study nested within two established longitudinal studies: TEDS and ALSPAC. The purpose of this research is to examine what depression, and its correlates look like in day-to-day, or moment-to-moment settings in people with different histories of depression trajectories. Building on decades of cohort data that have characterised the long-term epidemiology of depression from childhood into adulthood, our study seeks to extend traditional longitudinal research to examine both long- and short-term depression trajectories. We will examine how depression occurs in close to 'real time', and aim to identify proximal, modifiable factors that drive short-term depressive experiences. Our present study is motivated by the need for new evidence to inform interventions that are ecologically valid, timely, and responsive to people with different levels of depression risk.

A central strength of this study is the integration of intensive short-term data collection with extensive life-course phenotypic data. Existing EMA studies of depression have largely been conducted in opportunity or clinical samples without access to longitudinal background data [23], limiting their ability to contextualise short-term findings within the broader developmental context. In contrast, our study capitalises on the rich existing data of two large, well-characterised cohorts to select participants on the basis of previously established depression trajectory groups. This design enables a level of contextualisation that is not achievable in traditional EMA research, allowing short-term depressive dynamics to be examined against a backdrop of observed genetic, and potentially modifiable factors that may have accumulated across the life-course. This approach has the potential to yield insights into the real-time epidemiology of depression and ultimately to more time and person specific interventions and preventions.

A further strength lies in the multimodal nature of the data collection. We are collecting a variety of data encompassing sleep, diet, substance use, exercise, social interactions, daily activities, stress and other domains of mental health. This will allow us to thoroughly examine the epidemiology of depression in people with different depression trajectories for the first time. In addition, the combination of self-reported EMA data with passively collected objective physiological measures

For peer review only

such as sleep, activity and heart rate via wearable smartwatch devices provides a more complete picture of the biological and behavioural context in which depressive fluctuations occur than either data source could provide alone [35, 36, 43]. Self-report measures of some behaviours such as sleep and physical activity, while valuable, are subject to recall bias and may not accurately reflect objective physiological states [44]. Passive wearable data can help to validate such self-reported information, and together they can provide large amounts of powerful data that are suitable for advanced analytical methods such as machine learning and depression prediction [26, 45].

The aim of our study is to provide valuable insights into the epidemiology of depression and to inform the potential development of smartphone-based and ecological momentary interventions for depression [25]. Digital mental health interventions show considerable promise as scalable, accessible tools for improving depression outcomes, but their application is still very much in development. By identifying specific momentary risk and protective factors that strongly predict short-term depressive fluctuations, our study can provide a robust empirical foundation for the development of interventions that are triggered by, and responsive to, real-time signals of elevated risk. Such just-in-time adaptive interventions represent a promising frontier for depression treatment and prevention [23, 25].

Furthermore, we hope our study highlights a unique opportunity to conduct novel study designs that can be embedded within existing studies. Integrating intensive and/or passive smartphone and smartwatch data collection with the vast amount of data available in longitudinal cohort studies could hold significant opportunities to improve our understanding of depression and other diseases and behaviours.

Limitations

There are several limitations of our study that warrant acknowledgement. First, the EMA component captures depressive experience over a period of six weeks, providing a relatively brief window into short-term depressive fluctuations and not a continuous record of daily life over months or years. While such a study design is possible, the burden on participants to complete such measures over months or even years is likely to be too great. As such a key strength remains the ability to link these

For peer review only

short-term depressive symptoms with the longitudinal cohort data. Future studies could look to embed regular periodic assessments of EMA into existing longitudinal studies (i.e., once every couple of years) to address this.

Second, as with all EMA studies, our present design is likely to be subject to low compliance, participants rapidly completing the surveys and attrition over the study period. Participants who find the EMA schedule burdensome, or who experience significant worsening of depression during the study, may be less likely to complete all assessments or even consent to join the study to begin with, potentially introducing selection bias, as per some previous research [46-48]. Although our study protocol has been designed to minimise burden with surveys designed to take no more than a few minutes each, and appropriate incentives and support structures will be in place, some degree of non-random attrition is to be expected. Sensitivity analyses will be used to examine the potential impact of missing data on key findings.

Finally, while our study spans two different cohorts and is designed to maximise sample size within our available resources, statistical power for some of the more complex within-person and between-group analyses may be limited, particularly in the subsample completing smartwatches. Power analyses and simulation studies have been used to inform the study design but replication of key findings in independent samples will ultimately be necessary to establish their robustness.

For peer review only

Table 1. Study measures for the Mood in the Moment Study

Phase	Measure	Source(s) & Details	Items (n)	Data Collection Week						
**Pre-study**				**0**	**1**	**2**	**3**	**4**	**5**	**6**
	Informed consent		8	x	-	-	-	-	-	-
**EMA Questionnaires (baseline, two-week, four-week and end of survey only)**										
	Menstruation	Does participant currently menstruate	1	x	-	-	-	-	-	-
	Work pattern & caregiving	Adapted from prior shift-work studies; Typical vs. non-typical work pattern or caring responsibilities	1	x	-	-	-	-	-	-
	Depression	Short Mood and Feelings Questionnaire (SMFQ)[49]	13	x	-	x	-	x	-	x
	Anxiety	Generalised Anxiety Disorder Scale (GAD 7)[50]	7	x	-	x	-	x	-	x
	Resilience	Brief Resilience Scale (BRS)[51]	6	x	-	#	-	#	-	#
	Self esteem	Rosenberg Self-Esteem Scale (RSES)[52]	10	x	-	#	-	#	-	#
	Cognitive style	Global Cognitive Styles Questionnaire (CSQ)[53]	4	x	-	#	-	#	-	#
	Social support	Social Support Scale[54]	6	x	-	x	-	x	-	x
	Exercise	Adapted from prior studies; frequency of mild, moderate, & strenuous exercise	3	x	-	x	-	x	-	x
	Time outdoors	Newly developed; typical daily duration	1	x	-	x	-	x	-	x
	Sleep	Adapted from Pittsburgh Sleep Quality Index (PSQI); sleep quality & impact on daily life[55]	1 to 2*	x	-	x	-	x	-	x
	Pain	Newly developed; pain frequency & impact on life or work	1 to 2*	x	-	x	-	x	-	x
	Recent Life Events	Adapted from prior life events studies; occurrence of positive & negative life events	1	x	-	x	-	x	-	x
	Hormonal or contraceptive medication	Newly developed; medication type & duration of use	1 to 3*	x	-	x	-	x	-	x
	Gynaecological or reproductive health conditions	Newly developed; history of conditions	1	x	-	-	-	-	-	-
	Mental health conditions	Newly developed; history & type of mental health conditions	1 to 2*	x	-	-	-	-	-	-
	Mental health medication & treatment	Newly developed; recent use of mental health medication or treatment	1	x	-	x	-	x	-	x
	Perceived stress**	Perceived Stress Scale (PSS)[56]	14			x				

Personality**	Big Five Inventory – Short Form (BFI-SF)[57]	15					x		
Burnout**	Maslach Burnout Inventory (MBI-GS)[58]	16							x
**EMA Surveys (asking about last 3 hours, 3 x day)**									
Depression***	Adapted version of Patient Health Questionnaire (PHQ-8/9)[59-61]	8 or 9	-	ooo	ooo	ooo	ooo	ooo	ooo
Anxiety	Adapted version of Generalised Anxiety Disorder Scale (GAD-2) + 1 item on irritability from GAD-7[50]	3	-	ooo	ooo	ooo	ooo	ooo	ooo
Current location	Where participant has spent most time	1	-	ooo	ooo	ooo	ooo	ooo	ooo
Current activities	What participant has mostly been doing	1	-	ooo	ooo	ooo	ooo	ooo	ooo
Diet and food quality	Adapted from prior diet and food insecurity studies; Food consumed & type of food	1 to 2*	-	ooo	ooo	ooo	ooo	ooo	ooo
Physical activity	Highest level of physical activity (from no movement to strenuous exercise)	1	-	ooo	ooo	ooo	ooo	ooo	ooo
Substance use	Smoking, vaping, & alcohol consumption	1	-	ooo	ooo	ooo	ooo	ooo	ooo
Stress	Feelings of stress	1	-	ooo	ooo	ooo	ooo	ooo	ooo
Confiding in someone	Feeling able to confide in someone close[62]	1	-	ooo	ooo	ooo	ooo	ooo	ooo
Social connectedness	Newly developed; enjoyment of social contact online, in-person, & via social media	3	-	ooo	ooo	ooo	ooo	ooo	ooo
Sleep	Adapted from Pittsburgh Sleep Quality Index (PSQI); sleep quality, sleep & wake time[55]	1 to 3*	-	ooo	ooo	ooo	ooo	ooo	ooo
Daily Menstruation status****	Has period started in past 24 hours	1	-	ooo	ooo	ooo	ooo	ooo	ooo
**Passive Data Collection in ALSPAC only**									
Withings Steel HR or ScanWatch Light Smart Watch	Step count, heart rate and sleep		Continuous throughout 6 weeks						

Note. x = Administered at baseline or follow up
# = Administered at baseline or follow up in TEDS only
ooo = Administered three times a day across each week.
* Follow-up items dependent on answers provided to initial item.
** Only administered within ALSPAC.
*** PHQ-9 administered 1x per day in afternoon survey, PHQ-8 administered in morning and evening survey.
**** Only administered in evening survey to participants who indicated they menstruated in baseline questionnaire.

For peer review only

Acknowledgments

We gratefully acknowledge the ongoing contribution of the Twins Early Development Study (TEDS) participants and their families. We are extremely grateful to all the ALSPAC families who took part in this study, the midwives for their help in recruiting them, and the whole ALSPAC team, which includes interviewers, computer and laboratory technicians, clerical workers, research scientists, volunteers, managers, receptionists and nurses.

Contributions

Conceptualisation: ASFK. Funding acquisition: ASFK. Methodology: All authors. Writing original draft: ASFK. Writing review and editing: All authors.

Funding

This research was funded by Wellcome (Grant ref: 227063/Z/23/Z). The UK Medical Research Council and Wellcome (Grant ref: MR/Z505924/1) and the University of Bristol provide core support for ALSPAC. A comprehensive list of grants funding is available on the ALSPAC website: http://www.bristol.ac.uk/alspac/external/documents/grant-acknowledgements.pdf. TEDS is supported by the UK Medical Research Council (MR/V012878/1 and previously MR/M021475/1). This publication is the work of the authors and ASFK will serve as guarantor for the contents of this paper. ASFK is supported by a Wellcome Early Career Award (Grant ref: 227063/Z/23/Z). The funders did not influence the results or outcomes of the study, despite author affiliations with the funder (JH, EB, AS, SM and RH).

Data availability

The informed consent obtained from ALSPAC (Avon Longitudinal Study of Parents and Children) participants does not allow the data to be made available through any third party maintained public repository. Supporting data are available from ALSPAC on request under the approved proposal number, B4792. Full instructions for applying for data access can be found here:

For peer review only

http://www.bristol.ac.uk/alspac/researchers/access/. The ALSPAC study website contains details of all available data (http://www.bristol.ac.uk/alspac/researchers/our-data/).

Competing interests

None.

Patient consent for publication

Not applicable.

For peer review only

References

1. W.H.O, *World mental health today: latest data*. 2025.
2. W.H.O. *Depression: let's talk*. 2017 [cited 2017.
3. Thapar A, et al., *Depression in young people.* Lancet, 2022. **400**(10532): p. 617-631.
4. Armitage, J.M., et al., *Cross-cohort change in parent-reported emotional problem trajectories across childhood and adolescence in the UK.* Lancet Psychiatry, 2023. **10**(7): p. 509-517.
5. McGorry, P.D., et al., *The Lancet Psychiatry Commission on youth mental health.* Lancet Psychiatry, 2024. **11**(9): p. 731-774.
6. Lopez-Lopez, J.A., et al., *Trajectories of depressive symptoms and adult educational and employment outcomes.* BJPsych Open, 2019. **6**(1): p. e6.
7. Weavers, B., et al., *The antecedents and outcomes of persistent and remitting adolescent depressive symptom trajectories: a longitudinal, population-based English study.* Lancet Psychiatry, 2021. **8**(12): p. 1053-1061.
8. Copeland, W.E., et al., *Adult Functional Outcomes of Common Childhood Psychiatric Problems: A Prospective, Longitudinal Study.* JAMA Psychiatry, 2015. **72**(9): p. 892-9.
9. Fergusson, D.M., J.M. Boden, and J. Horwood, *Recurrence of major depression in adolescence and early adulthood, and later mental health, educational and economic outcomes.* Br J Psychiatry, 2007. **191**: p. 335-342.
10. Solmi, M., et al., *Age at onset of mental disorders worldwide: large-scale meta-analysis of 192 epidemiological studies.* Mol Psychiatry, 2022. **27**(1): p. 281-295.
11. Desai Bostrom, A.E., et al., *Recovery and Recurrence From Major Depression in Adolescence and Adulthood.* Acta Psychiatr Scand, 2025. **151**(5): p. 625-633.
12. Shore, L., et al., *Review: Longitudinal trajectories of child and adolescent depressive symptoms and their predictors - a systematic review and meta-analysis.* Child and Adolescent Mental Health, 2018. **23**(2): p. 107-120.
13. Schubert, K.O., et al., *Depressive symptom trajectories in late adolescence and early adulthood: A systematic review.* Aust N Z J Psychiatry, 2017. **51**(5): p. 477-499.
14. Musliner, K.L., et al., *Heterogeneity in long-term trajectories of depressive symptoms: Patterns, predictors and outcomes.* J Affect Disord, 2016. **192**: p. 199-211.
15. Ge, X. and M.N. Natsuaki, *In Search of Explanations for Early Pubertal Timing Effects on Developmental Psychopathology.* Current Directions in Psychological Science, 2009. **18**(6).
16. Kwong, A.S.F., et al., *Identifying Critical Points of Trajectories of Depressive Symptoms from Childhood to Young Adulthood.* J Youth Adolesc, 2019. **48**: p. 815-827.
17. Kwong, A.S.F., et al., *Polygenic risk for depression, anxiety and neuroticism are associated with the severity and rate of change in depressive symptoms across adolescence.* Journal of Child Psychology and Psychiatry, 2021.
18. Grimes, P.Z., et al., *Genetic Architectures of Adolescent Depression Trajectories in 2 Longitudinal Population Cohorts.* JAMA Psychiatry, 2024.

For peer review only

19. Cadman, T., et al., *Joint associations of parental personality traits and socio-economic position with trajectories of offspring depression: Findings from up to 6925 families in a UK birth cohort.* JCPP Adv, 2021. **1**(3): p. e12028.
20. Rajyaguru, P., et al., *Maternal and paternal depression and child mental health trajectories: evidence from the Avon Longitudinal Study of Parents and Children.* BJPsych Open, 2021. **7**(5): p. e166.
21. Fritz, J., et al., *So You Want to Do ESM? 10 Essential Topics for Implementing the Experience-Sampling Method.* Advances in Methods and Practices in Psychological Science, 2024. **7**(3): p. 1-27.
22. Siepe, B.S., et al., *Understanding Ecological-Momentary-Assessment Data: A Tutorial on Exploring Item Performance in Ecological-Momentary-Assessment Data.* Advances in Methods and Practices in Psychological Science, 2025. **8**(1): p. 1-20.
23. Harari, G.M. and S.D. Gosling, *Understanding behaviours in context using mobile sensing.* Nature Reviews Psychology, 2023. **2**(12): p. 767-779.
24. Dejonckheere, E., et al., *Complex affect dynamics add limited information to the prediction of psychological well-being.* Nat Hum Behav, 2019. **3**(5): p. 478-491.
25. Fried, E.I., R.K.K. Proppert, and C.L. Rieble, *Building an Early Warning System for Depression: Rationale, Objectives, and Methods of the WARN-D Study.* Clin Psychol Eur, 2023. **5**(3): p. e10075.
26. Ware, S., et al., *Predicting depressive symptoms using smartphone data.* Smart Health, 2020. **15**.
27. Volovic-Shushan, S., et al., *Real-time experience: A systematic review of ecological momentary assessments of everyday functioning in major depressive disorder.* Aust Occup Ther J, 2026. **73**(2): p. e70080.
28. Unzueta Saavedra, J., et al., *Smartphone Ecological Momentary Assessment and Wearable Activity Tracking in Pediatric Depression: Cohort Study.* JMIR Form Res, 2025. **9**: p. e66187.
29. Triantafillou, S., et al., *Relationship Between Sleep Quality and Mood: Ecological Momentary Assessment Study.* JMIR Ment Health, 2019. **6**(3): p. e12613.
30. El-Awad, U., et al., *Physical activity is associated with subsequent affective well-being in day hospital patients with a diagnosis of depression: A combined ecological momentary assessment and accelerometry study.* Mental Health and Physical Activity, 2025. **29**.
31. Li, Y.M., et al., *Temporal associations between objectively measured physical activity and depressive symptoms: An experience sampling study.* Front Psychiatry, 2022. **13**: p. 920580.
32. Perski, O., et al., *Understanding health behaviours in context: A systematic review and meta-analysis of ecological momentary assessment studies of five key health behaviours.* Health Psychol Rev, 2022. **16**(4): p. 576-601.
33. Kuczynski, A.M., et al., *Characterizing the momentary association between loneliness, depression, and social interactions: Insights from an ecological momentary assessment study.* J Affect Disord, 2024. **360**: p. 376-386.
34. Speyer, L.G., et al., *The role of moment-to-moment dynamics of perceived stress and negative affect in co-occurring ADHD and internalising symptoms.* J Autism Dev Disord, 2023. **53**(3): p. 1213-1223.

For peer review only

35. Price, G.D., et al., *Use of passively collected actigraphy data to detect individual depressive symptoms in a clinical subpopulation and a general population.* J Psychopathol Clin Sci, 2025. **134**(1): p. 31-40.
36. Price, G.D., et al., *Detecting major depressive disorder presence using passively-collected wearable movement data in a nationally-representative sample.* Psychiatry Res, 2024. **332**: p. 115693.
37. Lockhart, C., et al., *Twins Early Development Study (TEDS): A genetically sensitive investigation of mental health outcomes in the mid-twenties.* JCPP Adv, 2023. **3**(2): p. e12154.
38. Boyd, A., et al., *Cohort Profile: the 'children of the 90s'--the index offspring of the Avon Longitudinal Study of Parents and Children.* Int J Epidemiol, 2013. **42**(1): p. 111-27.
39. Fraser, A., et al., *Cohort Profile: the Avon Longitudinal Study of Parents and Children: ALSPAC mothers cohort.* Int J Epidemiol, 2013. **42**(1): p. 97-110.
40. Northstone, K., et al., *The Avon Longitudinal Study of Parents and Children (ALSPAC): an update on the enrolled sample of index children in 2019.* Wellcome Open Res, 2019. **4**: p. 51.
41. Grimes, P.Z., et al., *Genome-wide association study of adolescent-onset depression.* Under review.
42. Murray, A., et al., *Illuminating the daily life experiences of adolescents with and without ADHD: protocol for an ecological momentary assessment study.* BMJ Open, 2023. **13**(9): p. e077222.
43. Ringwald, W.R., et al., *Passive Smartphone Sensors for Detecting Psychopathology.* JAMA Netw Open, 2025. **8**(7): p. e2519047.
44. Lekkas, D., et al., *Depression deconstructed: Wearables and passive digital phenotyping for analyzing individual symptoms.* Behav Res Ther, 2023. **168**: p. 104382.
45. Kim, E.Y., et al., *Diagnosis of major depressive disorder by combining multimodal information from heart rate dynamics and serum proteomics using machine-learning algorithm.* Prog Neuropsychopharmacol Biol Psychiatry, 2017. **76**: p. 65-71.
46. Murray, A., et al., *Predicting Participation Willingness in Ecological Momentary Assessment of General Population Health and Behavior: Machine Learning Study.* J Med Internet Res, 2023. **25**: p. e41412.
47. Murray, A., et al., *Respondent characteristics associated with adherence in a general population ecological momentary assessment study.* International Journal of Methods in Psychiatric Research, 2023. **32**(4).
48. Murray, A.L., et al., *Prompt-level predictors of compliance in an ecological momentary assessment study of young adults' mental health.* J Affect Disord, 2023. **322**: p. 125-131.
49. Angold, A., et al., *Development of a short questionnaire for use in epidemiological studies of depression in children and adolescents.* International Journal of Methods in Psychiatric Research, 1995. **5**(4): p. 237-249.
50. Spitzer, R.L., et al., *A Brief Measure for Assessing Generalized Anxiety Disorder.* Arch Intern Med, 2006. **166**: p. 1092-1097.
51. Smith, B., et al., *The Brief Resilience Scale: Assessing the Ability to Bounce Back.* International Journal of Behavioral Medicine, 2008. **15**: p. 194-200.
52. Rosenberg, M., *Rosenberg self-esteem scale.* Journal of Religion and Health, 1965.

For peer review only

53. Pearson, R.M., et al., *Association Between Maternal Depressogenic Cognitive Style During Pregnancy and Offspring Cognitive Style 18 Years Later.* Am J Psychiatry, 2013. **170**(4): p. 434-441.
54. Shakespeare-Finch, J. and P.L. Obst, *The Development of the 2-Way Social Support Scale: A Measure of Giving and Receiving Emotional and Instrumental Support.* Journal of Personality Assessment, 2011. **93**(483-490).
55. Buysse, D.J., et al., *The Pittsburgh Sleep Quality Index: a new instrument for psychiatric practice and research.* Psychiatry Research, 1989. **28**(2): p. 193-213.
56. Cohen, S., T. Kamarck, and R. Mermelstein, *A global measure of perceived stress.* Journal of Health and Social Behavior, 1983. **24**(4): p. 385-396.
57. Lang, F.R., et al., *Short assessment of the Big Five: robust across survey methods except telephone interviewing.* Behavior research methods, 2011. **43**(2): p. 548–567.
58. Maslach, C. and S.E. Jackson, *The Measurement of Experienced Burnout.* Journal of Occupational Behaviour, 1981. **2**(2): p. 99-113.
59. Kroenke, K., R.L. Spitzer, and J.B. Williams, *The PHQ-9: Validity of a Brief Depression Severity Measure.* J Gen Intern Med, 2001. **16**(9): p. 606-613.
60. Kroenke, K., et al., *The PHQ-8 as a measure of current depression in the general population.* Journal of Affective Disorders, 2009. **114**(1): p. 163-173.
61. Haddox, D., et al., *Psychometric properties and validity of a Mobile Patient Health Questionnaire-9 (MPHQ-9) for ecological momentary assessment in depressed adults.* Psychol Assess, 2025.
62. Choi, K.W., et al., *An Exposure-Wide and Mendelian Randomization Approach to Identifying Modifiable Factors for the Prevention of Depression.* Am J Psychiatry, 2020. **177**(10): p. 944-954.

For peer review only

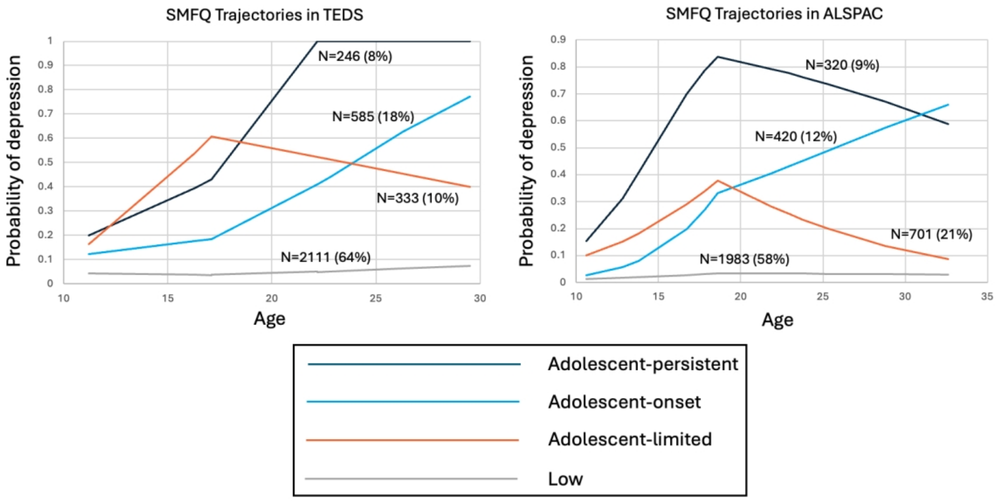

Figure 1. Depression trajectories in each cohort. TEDS: Twins Early Development Study; ALSPAC: Avon Longitudinal Study of Parents and Children. N (and percentage of total sample) are given for each trajectory.

566x288mm (72 x 72 DPI)

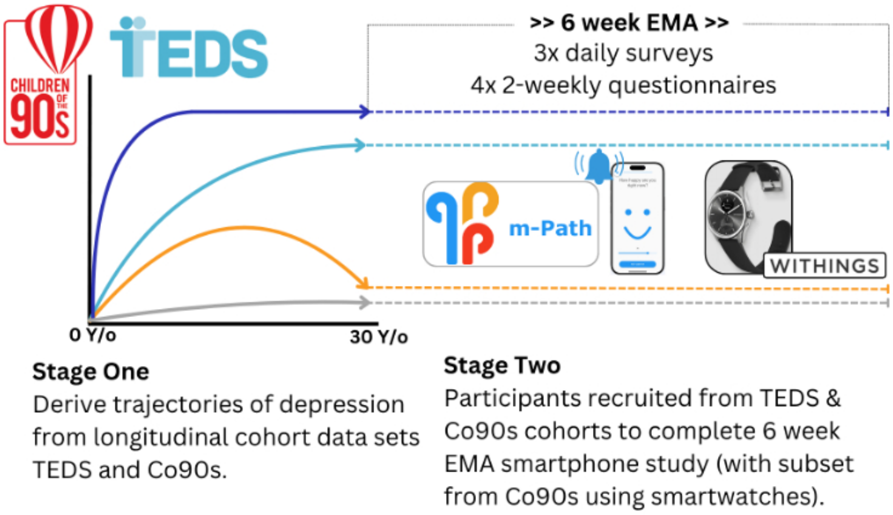

Figure 2. Summary of the Mood in the Moment Study design with schematic depression trajectories. TEDS: Twins Early Development Study; ALSPAC/Co90s: Avon Longitudinal Study of Parents and Children/Children of the 90s; EMA: Ecological Momentary Assessment.

218x122mm (96 x 96 DPI)

Supplementary materials

Mood in the Moment: a study protocol for embedding ecological momentary assessments into established longitudinal cohorts to examine depression in real-time

Additional cohort information - ALSPAC

Pregnant women resident in Avon, UK with expected dates of delivery between 1st April 1991 and 31st December 1992 were invited to take part in the study. 20,248 pregnancies have been identified as being eligible and the initial number of pregnancies enrolled was 14,541. Of the initial pregnancies, there was a total of 14,676 foetuses, resulting in 14,062 live births and 13,988 children who were alive at 1 year of age. When the oldest children were approximately 7 years of age, an attempt was made to bolster the initial sample with eligible cases who had failed to join the study originally. As a result, when considering variables collected from the age of seven onwards (and potentially abstracted from obstetric notes) there are data available for more than the 14,541 pregnancies mentioned above: The number of new pregnancies not in the initial sample (known as Phase I enrolment) that are currently represented in the released data and reflecting enrolment status at the age of 24 is 906, resulting in an additional 913 children being enrolled (456, 262 and 195 recruited during Phases II, III and IV respectively). The phases of enrolment are described in more detail in the cohort profile paper and its update. The total sample size for analyses using any data collected after the age of seven is therefore 15,447 pregnancies, resulting in 15,658 foetuses. Of these 14,901 children were alive at 1 year of age. From the age of 30, there were an additional 101 new participants recruited from 101 pregnancies during Phase V enrolment. The total sample size for analyses using any data collected after the age of 30 is therefore 15,548 pregnancies, resulting in 15,690 foetuses. Of these 15,002 children were alive at 1 year of age. Study data were collected and managed using REDCap (Research Electronic Data Capture) electronic data capture tools hosted at the University of Bristol. REDCap is a secure, web-based software platform designed to support data capture for research studies (Harris et al., 2009).

Reference: Harris, P., et al., *Research electronic data capture (REDCap) – A metadata-driven methodology and workflow process for providing translational research informatics support.* J Biomed Inform, 2009. **42**(2): p. 377-81.

For peer review only

Survey Measures used in Mood and the Moment Study

EMA Schedule and Key Information

Brief Daily Surveys (3 x per day unless stated)

Number of measures per survey: 11 – 13.

Approx time to complete: 3 minutes

The content of the 3 x daily brief daily surveys is almost identical except for order of administration. A summary of each survey (morning, afternoon, evening) is provided below, along with timings of the survey notifications and reminders sent in the m-Path app. A brief description of each survey item and its application in the current EMA study follows, and a detailed description of all survey and questionnaire items including item and response wording and references are provided at the end of the document.

Morning Survey Order	Afternoon Survey Order	Evening Survey Order
Current location and activity	PHQ-9	GAD-2
Diet	Current location and activity	Confiding in others
PHQ-8	Diet	Social connectedness
GAD-2	Exercise	Social media
Sleep	Substance use	Menstruation
Exercise	GAD-2	PHQ-8
Substance use	Stress	Stress
Stress	Positive and negative affect	Positive and negative affect
Positive and negative affect	Social media use	Current location and activity
Confiding in others	Social connectedness	Sleep*
Social connectedness	Confiding in others	Substance use
Social media use	Sleep*	Exercise
		Diet
**Notification time: 8:00am to 9:00am**	**Notification time: 1:00pm to 2:00pm**	**Notification time: 6:00pm to 7:00pm**
**Survey open for 3 hour**	**Survey open for 3 hour**	**Survey open for 3 hour**
**Reminders x 2, every 60 minutes**	**Reminders x 2, every 60 minutes**	**Reminders x 2, every 60 minutes**
	***only asked if previously missed**	***only asked if previously missed**

PHQ-8

- Administered with morning and evening prompts only
- 8 items
- Clinically validated measure
- Adapted version for EMA: replaced ‘Over the last 2 weeks’ with ‘In the last 3 hours’

PHQ 9

- Administered with afternoon prompt only
- 9 items

For peer review only

- Clinically validated measure
- Adapted version for EMA: replaced 'Over the last 2 weeks' with 'In the last 3 hours'

GAD-2 (+ irritability item from GAD-7)

- 3 items
- Clinically validated measure
- Adapted version for EMA: replaced 'Over the last 2 weeks' with 'In the last 3 hours'

Confiding in others

- 1 item
- Validated measure

Positive and negative affect

- 2 items
- Developed by research team at UofE

Stress

- 1 item
- Developed by research team at UofE

Sleep

- 3 items total
- Validated measure: single item on sleep quality from PSQI + 2 items on sleep/awake time
- **Once a day only**

Diet (food quality)

- 2 items
- Developed by research team at UofE

Menstruation

- Administered with evening prompt only
- 1 item
- Developed by research team at UofE

Exercise

- 1 item

For peer review only

- Developed by research team at UofE

Substance use (smoking/vaping & alcohol)

- 1 item
- Developed by research team at UofE

Current location & current activity

- 2 items
- Developed by research team at UofE

Social connectedness

- 2 items
- Developed by research team at UofE

Social media use

- 1 item
- Developed by research team at UofE

Baseline, week-2, -4 and -6 exit Questionnaires

Approx time to complete: 5 minutes

A summary of the questionnaire content is provided below, along with timings of the questionnaire notifications and reminders sent in the m-Path app. A brief description of each questionnaire item and its application in the current study follows, and a detailed description of all survey and questionnaire items including item and response wording and references are provided at the end of the document.

Short mood and feelings questionnaire (SMFQ)

- 13 items
- Clinically validated measure
- Assessed at every sweep

GAD-7

- 7 items
- Clinically validated measure
- Assessed at every sweep

For peer review only

Brief Resilience Scale (BRS)

- 6 items
- Validated measure
- ***Assessed at every sweep in TEDS, baseline questionnaire only in ALSPAC***

Rosenberg Self-Esteem Scale (RSE)

- 10 items
- Validated measure
- ***Assessed at every sweep in TEDS, baseline questionnaire only in ALSPAC***

Global cognitive styles

- 4 items
- Validated measure
- ***Assessed at every sweep in TEDS, baseline questionnaire only in ALSPAC***

Perceived Stress Scale

- 14 items
- Validated measure
- ***2 week follow up questionnaire in ALSPAC only***

Big Five Inventory 15 Item Version

- 15 items
- Validated measure
- ***4 week follow up questionnaire in ALSPAC only***

Maslach Burnout Inventory – MBI-GS

- 16 items
- Validated measure
- ***6 week follow up questionnaire in ALSPAC only***

Hormonal Contraception/Medication Use

- *3 items*
- Developed by research team at UofE
- Assessed at every sweep

Gynaecological or Reproductive Health Conditions

- *1 item*
- Developed by research team at UofE

For peer review only

- ***Baseline questionnaire only***

Pain

- 2 items
- Developed by research team at UofE
- Assessed at every sweep

Working pattern

- 1 item
- Developed by research team at UofE
- Assessed at every sweep

Exercise

- 3 items
- Developed by research team at UofE
- Assessed at every sweep

Time spent outdoors

- 1 item
- Developed by research team at UofE
- Assessed at every sweep

Social Support

- 6 items
- Validated measure
- Assessed at every sweep

Life in the last 2 weeks (exercise, sleep, pain and life events)

- 9 items
- Developed by research team at UofE
- Assessed at every sweep

Antidepressant medication or therapy use in the last 2 weeks

- 1 item
- Developed by research team at UofE
- Assessed at every sweep

Current and previous history of mental health

For peer review only

- 1 item
- Developed by research team at UofE
- ***Baseline questionnaire only***

Exit survey (only at week 6 at the end of the study)

- 1 item
- Developed by research team at UofE

For peer review only

EMA items (Validated Measures from Surveys and Questionnaires)

Adapted Patient Health Questionnaire (PHQ-8 and PHQ-9) (Kroenke et al. 2001)

The PHQ-8 is an 8-item self-administered measure which can be used to screen for depression and assess the severity of depressive symptoms in the previous two weeks. Here we adapt the time frame for the items to ask, ***'In the last 3 hours,** I have…' to make it suitable for use as an EMA measure*, and we adapt the sleep question to, 'Last night, I had trouble with sleep' (included in morning survey only). The PHQ-8 is included in the morning and evening surveys.

The PHQ-9, administered in the afternoon survey only, includes an additional item assessing suicidality. Participants answer using a sliding scale with a range between 0 and 100 with the further option for prefer not to say.

Participants will be informed in the participant information sheet and the consent form that we will not be actively monitoring their responses and as such they cannot use the m-Path app and their response to this item to attain help and support. However, if this question is answered positively (i.e., ≥ 1), participants will instantly be directed to help lines and advised to seek support through the application before being able to proceed with the survey.

PHQ-8 and PHQ-9 items [daily surveys]:

In the last 3 hours, I have **had little interest or pleasure in doing things**

0. Not at all

to

100. Constantly

Prefer not to say

In the last 3 hours, I have **felt down, depressed, or hopeless**

0. Not at all

to

100. Constantly

Prefer not to say

For peer review only

Last night, I **had trouble with sleep**

0. Not at all

to

100. Constantly

Prefer not to say

In the last 3 hours, I have **felt tired or have had little energy**

0. Not at all

to

100. Constantly

Prefer not to say

In the last 3 hours, I have **had poor appetite or have been overeating**

0. Not at all

to

100. Constantly

Prefer not to say

In the last 3 hours, I have **felt bad about myself**

0. Not at all

to

100. Constantly

Prefer not to say

In the last 3 hours, I have **had trouble concentrating**

0. Not at all

to

100. Constantly

Prefer not to say

For peer review only

In the last 3 hours, I have **been moving or speaking slowly, or fidgeting more**

0. Not at all

to

100. Constantly

Prefer not to say

In the last 3 hours, I have **had thoughts of hurting myself or that I would be better off dead** *[PHQ-9 question, included in the afternoon survey only. Participant directed to support lines if respond with answers ≥1]*

0. Not at all

to

100. Constantly

Prefer not to say

Reference: Kroenke, K., Spitzer, R. L., & Williams, J. B. W. (2001). The PHQ-9 - Validity of a brief depression severity measure. Journal of General Internal Medicine, 16, 606-613.

Adapted Generalised Anxiety Disorder Scale [Two-item version + irritability item] (GAD-2) (Spitzer et al. 2006)

The GAD-2 uses the first two items of the GAD-7 to assess critical facets of anxiety disorders. This shorter measure has shown to have good sensitivity and specificity to that of the GAD-7 (Plummer et al. 2016). Here we use the GAD-2 along with an additional question on irritability from the GAD-7, to be administered in all 3 brief daily surveys. We adapt the time frame for the items to ask, *'**In the last 3 hours,** have you…' to make it suitable for use as an EMA measure,* as with the PHQ-8/9 above.

Generalised Anxiety Disorder 2 + irritability [daily surveys]

In the last 3 hours, have you been **feeling nervous, anxious or on edge?**

0. Not at all

to

100. Constantly

Prefer not to say

In the last 3 hours, have you been **unable to stop or control worrying?**

0. Not at all

to

100. Constantly

Prefer not to say

In the last 3 hours, have you been **feeling easily annoyed or irritable?**

0. Not at all

to

100. Constantly

Prefer not to say

Generalised Anxiety Disorder Scale [Seven-Item] (GAD-7) (Spitzer et al. 2006)

The GAD-7 is a seven-item measure which screens for anxiety severity in clinical and general populations. Participants will also be administered the full GAD-7 during the baseline questionnaire and at every 2-week follow up questionnaire. The original item framing of '*Over the last 2 weeks...*' will be used for administering the GAD-7 in these bi-weekly questionnaires, but for ease of comprehension we have removed the "been bothered with" phrasing in each question. See reference for full list of questions.

Reference: Spitzer, R. L., Kroenke, K., Williams, J. B., & Löwe, B. (2006). A brief measure for assessing generalized anxiety disorder: the GAD-7. Archives of internal medicine, 166(10), 1092-1097.

Social Support Scale (Shakespeare-Finch & Obst, 2011)

The social support scale is a six-item scale to assess self-reported/perceived social supports. This measure will be administered at baseline and at every two-week follow-up questionnaire. See reference for full list of questions.

Reference: Shakespeare-Finch, J., & Obst, P.L. (2011). The Development of the 2-Way Social Support Scale: A Measure of Giving and Receiving Emotional and Instrumental Support. Journal of Personality Assessment, 93, 483 - 490.

For peer review only

Short Mood and Feelings Questionnaire (SMFQ) (Angold & Costello, 1987)

The SMFQ is a 13-item self-report questionnaire in which participants answer items to assess key depression symptoms. This measure has been used in clinical and general populations. Participants will be administered the SMFQ at baseline and at every two-week follow-up questionnaire. See reference for full list of questions.

Reference: Angold, A., & Costello, E. J. (1987). *Short Mood and Feelings Questionnaire (SMFQ, SMFQ-C, SMFQ-P)* [Database record]. APA PsycTests. https://doi.org/10.1037/t15197-000

Brief Resilience Scale (BRS) (Smith et al. 2008)

The BRS is a brief self-report scale in which participants answer six items to measure their ability to bounce back or recover from stress. This measure will be administered at baseline in ALSPAC and TEDS and at every two-week follow up in TEDS only. See reference for full list of questions.

Reference: Smith, B. W., Dalen, J., Wiggins, K., Tooley, E., Christopher, P., & Bernard, J. (2008). The brief resilience scale: assessing the ability to bounce back. *International journal of behavioral medicine*, *15*(3), 194–200. https://doi.org/10.1080/10705500802222972

Rosenberg Self-Esteem Scale (RSE) (Rosenberg, 1965)

The RSE is a ten-item self-report measure used to assess global self-esteem in adults. This measure will be administered at baseline in ALSPAC and TEDS and at every two-week follow up in TEDS only. See reference for full list of questions.

Reference: Rosenberg, M. (1965). *Rosenberg Self-Esteem Scale (RSES)* [Database record]. APA PsycTests. https://doi.org/10.1037/t01038-000

Global Cognitive Styles Questionnaire (CSQ) (Pearson et al. 2013)

The CSQ is a measure utilised to assess a participant's negative cognitive style through four items. This measure will be administered at baseline in ALSPAC and TEDS and at every two-week follow up in TEDS only. See reference for full list of questions.

Reference: Pearson, R. M., Fernyhough, C., Bentall, R., Evans, J., Heron, J., Joinson, C., Stein, A. L., & Lewis, G. (2013). Association between maternal depressogenic cognitive style during pregnancy and offspring cognitive style 18 years later. The American journal of psychiatry, 170(4), 434–441. https://doi.org/10.1176/appi.ajp.2012.12050673

For peer review only

The Perceived Stress Scale (Cohen et al., 1983)

The PSS is used to measure perceived stress in the last month and will be assessed in ALSPAC at the 2-week questionnaire only. See reference for full list of questions.

Reference: Cohen, S., Kamarck, T., & Mermelstein, R. (1983). Perceived Stress Scale [Database record]. APA PsycTests. https://doi.org/10.1037/t02889-000

Big Five Inventory – 15 Item Short Form (Lang et al. 2011)

The Big Five Inventory - 15 item Short Form is a shorter version of the traditional Big Five Inventory for assessing personality. This will be measured administered in ALSPAC at the 4 week follow up only. See reference for full list of questions.

Reference: Lang, F. R., John, D., Lüdtke, O., Schupp, J., & Wagner, G. G. (2011). Short assessment of the Big Five: robust across survey methods except telephone interviewing. Behavior research methods, 43(2), 548–567. https://doi.org/10.3758/s13428-011-0066-z

Maslach Burnout Inventory: MBI-GS – (Maslach & Jackson, 1981)

The MBI-GS is a short form measure of the original Maslach Burnout Inventory and is a concise measure of burnout. It will be administered in ALSPAC at the 6-week questionnaire only. See reference for full list of questions.

Maslach, C., & Jackson, S. E. (1981). The Measurement of Experienced Burnout. *Journal of Occupational Behaviour*, *2*(2), 99–113. https://doi.org/10.1002/job.4030020205

EMA Items (Non-Validated Measures from Surveys and Questionnaires)

Current Location and Activities

This two-item measure assess where participants have spent the most time between prompts and to report on their main activities during this time. Items assessing location and current activities may give insight into how a participant's environment or activity may be related to their reported mood and other factors reported via daily EMA measures. These items are included in all 3 brief daily surveys.

In the last 3 hours, **where have you spent the most time?**

1. At home

For peer review only

2. At a friend's place
3. At my romantic partner's place
4. At my family's place
5. At work / place of education
6. In a car / on public transport
7. Other indoor location
8. Outdoor location (urban/city)
9. Outdoor location (rural/in nature)
10. At a hotel or Airbnb

In the last 3 hours, **what have you mainly been doing?** Select all that apply:

1. Social activities (in person)
2. Online social activities
3. Physical activity/leisure (e.g., walking, exercising)
4. Mentally stimulating activities (e.g., reading a book, puzzles, board game)
5. Passive leisure (e.g., watching TV, scrolling social media)
6. Studying / working
7. Chores (e.g., groceries, cooking, cleaning)
8. Childcare
9. Travelling / on my way somewhere
10. Eating
11. Sleeping
12. Other

Working patterns

To gather data on non-standard working patterns and other reasons for participants systematically missing surveys and questionnaires, they will be asked the following question:

It's important that you answer as many surveys and questionnaires as possible throughout the study, however we understand that work and other commitments might stop you from doing so. We'd still love you to take part, even if you can't respond to every notification.

Please select one of the options below to let us know about any commitments that will regularly stop you from responding to notifications in the next 6 weeks:

1. I work shifts or have a non-typical working pattern (e.g., work starts before 7am or after 2pm, hours rotate, or regularly includes hours outside of 7am to 6pm)
2. I have caring responsibilities
3. I am unable to access my phone during working hours
4. N/A – I can normally respond to notifications at any time.

For peer review only

Stress

Stress will be assessed using the following single item assessed with a sliding scale, included in all 3 brief daily surveys.

In the last 3 hours, **have you been feeling stressed?**

0. Not at all

to

100. Extremely

Prefer not to say

Confiding in Someone

Participants are asked a single item *'In the last 3 hours, have you felt like you could confide in someone close to you?'*. This question is included in all 3 brief daily surveys.

In the last 3 hours, **have you *felt like* you could confide in someone close to you?**

0. Not at all

1. Some of the time

2. More than half the time

3. Nearly all the time

4. Prefer not to say

Positive and Negative Affect

Positive and negative affect will be assessed using the following two items assessed with a sliding scale for the first one and a selection of options for the second, included in all 3 brief daily surveys in ALSPAC only.

How positive or negative do you feel right now?

0. Positive

to

For peer review only

10. Negative

Prefer not to say

Reflect on what you were thinking about prior to answering this survey

1. I was conscious of my inner feelings.

2. I was self-conscious about the way I look or was concerned about the way I present myself.

3. I was conscious of what is going on around me.

4. I was reflective about my life.

5. I was concerned about what other people think of me

6. I was aware of my innermost thoughts.

7. None of the above

Prefer not to say

Exercise

Participants will be asked a variety of items on exercise across the surveys and questionnaires. The questions for the daily surveys, were adapted from a question in Hirvensalo et al. (2000) and response options from Bruening et al. (2016).

Additionally, at baseline and for every two-week follow-up, participants will be asked three further items on exercise and time spent outdoors.

[Following Question is asked in brief daily surveys]

In the last 3 hours, what has been your highest level of physical activity?

0. No movement (e.g., sitting at a desk, on the sofa, travelling by car or public transport)
1. Moved only in connection with necessary tasks (e.g., household chores)
2. Gentle exercise or leisurely activities requiring little effort (e.g., gentle walking, gardening, yoga)
3. Moderate exercise - increased your heart rate but not exhausting e.g., brisk walking, dancing, cycling, weights)
4. Strenuous exercise causing sweating / heavy breathing (e.g., running, football, fast swimming, HIIT)

For peer review only

[The following questions are asked in bi-weekly questionnaires]

In the past 2 weeks, how many times have you done any strenuous exercise to the point of sweating and heavy breathing (e.g., running, HIIT, football, fast swimming)?

1. None
2. 1 – 2 times
3. 3 – 4 times
4. 5 – 7 times
5. 8 – 10 times
6. 11 - 13 times
7. 14 or more times

In the past 2 weeks, how many times have you done any moderate exercise that increased your heart rate but was not exhausting (e.g., walking fast, hiking, lifting weights, dancing)?

1) None
2) 1 – 2 times
3) 3 – 4 times
4) 5 – 7 times
5) 8 – 10 times
6) 11 - 13 times
7) 14 or more times

In the past 2 weeks, how many times have you done any mild exercise that required little effort (e.g., gentle walking, yoga, bowling)

1) None
2) 1 – 2 times
3) 3 – 4 times
4) 5 – 7 times
5) 8 – 10 times
6) 11- 13 times
7) 14 or more times

Thinking about the past 2 weeks, how many minutes did you spend outdoors on a typical day?

1) 0-15 minutes
2) 16-60 minutes
3) 61-120 minutes (1-2 hours)
4) 121-180 minutes (2-3 hours)
5) 181+ minutes (3+ hours)

For peer review only

Sleep

Participants will be asked to rate the quality of their sleep using items from the Pittsburgh Sleep Quality Index (PSQI) (Buysse et al. 1989). The sleep quality item will be included in all 3 daily surveys to reduce possible data loss in participants with atypical sleep patterns (e.g., shift-workers). However, the sleep and wake time questions will only be asked once per day.

Further, during baseline and bi-weekly questionnaires participants will be asked again to rate the overall quality of their sleep. If a participant indicates that their average sleep has been of bad quality (answers ≥4) they will be prompted to answer a further question about how their sleep affects their daily life.

[Following Question is asked in brief daily surveys]

How would you rate the quality of your sleep last night?

0. Very poor
1. Poor
2. Neutral
3. Good
4. Very good

What time did you fall asleep last night ________ [answer in hours/minutes]

What time did you wake up today ____________ [answer in hours/minutes]

[Following Questions are asked in bi-weekly questionnaires]

In the past 2 weeks, how would you rate the quality of your sleep on average?

1. Very good
2. Fairly good
3. Average
4. Fairly poor
5. Very poor

In the past 2 weeks, have your sleep difficulties interfered with your daily life? [only for participants who answer 4 or 5 to preceding question]

1. Not at all

For peer review only

2. A little bit
3. Moderately
4. Quite a bit
5. Extremely

Diet

To assess diet and diet quality in participants we will administer two items. The first will ask participants if they have eaten since the last prompt. Those who indicate that they have eaten, they will be directed to the second item asking about the type of food they have eaten (e.g., processed or homemade food). These items are adapted from Berge et al. (2023) and will be included in all 3 daily surveys.

Have you eaten in the last 3 hours? Select all that apply:

1. Yes, I have eaten a main meal (breakfast, lunch, or dinner)
2. Yes, I have eaten a snack
3. No, I have not eaten since the last prompt

Which best describes the type of food you ate on this occasion? Select all that apply: **[only for participants who answer 1 or 2 to preceding question]**

1. Fast food / take-out (consumed at fast food restaurant, home, or other location)
2. Other food eaten outside the home (e.g., a restaurant, café)
3. Pre-prepared or processed foods (e.g., frozen, oven-cook or microwaveable meals, breakfast cereals, crisps, biscuits, etc.)
4. Homemade / freshly prepared food (e.g., made from fresh fruits or vegetables, meat, fish, dairy, grains, pulses)

Menstruation

A single item will enquire as to whether participants have menstruated in the last 24 hours. There will be a '*prefer not to say*' option for those who do not wish to answer.

Did you have your period **in the last 24 hours?**

1. Yes
2. No

For peer review only

Hormonal Contraception/Medication Use and Gynaecological or Reproductive Health Conditions

Three questions will enquire as to whether participants are currently taking any hormonal contraceptives or medications at baseline and every 2-weekly questionnaire. A single question about experience of gynaecological or reproductive health conditions will be included in the baseline questionnaire.

Are you currently using any hormonal contraceptives or medications? This can be for any reason (e.g., birth control, acne, pain/cramps)

1. Yes
2. N/A
3. I don't currently take any hormonal contraception or medication
4. I've stopped taking hormonal contraceptives or medications within the last three months.
5. I use a non-hormonal copper coil/IUD

Please select the type of hormonal contraceptives or medications you are currently taking (select all that apply)

[Only for participants who answer 'Yes' to previous question]

1. Combined pill (e.g., Microgynon, Rigevidon, Ovranette)
2. Progestogen only pill ("mini-pill" e.g., Cerazette, Cerelle, Norgeston)
3. Contraceptive injection (e.g., Depo-Provera, Sayana Press)
4. Contraceptive patches (e.g., Evra Patches)
5. Vaginal ring (e.g., NuvaRing)
6. Hormonal coil/IUS (Mirena, Levosert, Benilexa, Kyleena, Jaydess)
7. Contraceptive implant (e.g., Nexplanon)
8. Other (please give details): [Prompts free text box]

How long have you been taking any type of hormonal contraceptive for?

[Only for participants who answer 'Yes' to taking hormonal contraception/medication]

1. 0 – 3 months
2. 3 – 6 months
3. 6 – 12 months
4. 1 year – 2 years
5. 2 years plus

For peer review only

Have you ever had any of the following gynaecological or reproductive health conditions? Select all that apply:

[baseline questionnaire only]

1. N/A
2. No
3. Endometriosis
4. Polycystic ovary syndrome (PCOS)
5. Fibroid(s)
6. Ovarian cyst
7. Pelvic inflammatory disease (PID)
8. Other (please give details) [Prompts free text box]

Substance Use (Smoking and Alcohol)

We will assess substance use with a single item in all 3 brief daily surveys.

In the last 3 hours, have you done any of the following? Select all that apply:

1. Smoked
2. Vaped
3. Drank alcohol
4. None of the above

Social Connectedness

Social connectedness will be assessed with three items asking participants how enjoyable they have found different types of social contact, including in-person and online. Items are adapted from Fried et al. (2023). These will be administered in all 3 brief daily surveys.

In the last 3 hours, **how enjoyable was spending time with other people in person?**

1. Not at all
2. Somewhat
3. Moderately
4. Very
5. Extremely
6. I haven't had any in-person contact in the last 3 hours

In the last 3 hours, **how enjoyable was spending time online using social media sites or apps such as Instagram, TikTok or X?**

1. Not at all
2. Somewhat

For peer review only

3. Moderately
4. Very
5. Extremely
6. I haven't spent any time using social media in the last 3 hours

In the last 3 hours, **how enjoyable was spending time with other people online through messaging sites (e.g., WhatsApp), video calls, or by phone?**

1. Not at all
2. Somewhat
3. Moderately
4. Very
5. Extremely
6. I haven't had any online social interactions in the last 3 hours

Pain

Participants will be asked about their experience of pain in the baseline questionnaire and at every two-week follow-up questionnaire. Those who report experiencing any pain will be asked a follow up question about the impact this has had on their life or work activities.

In the past 2 weeks, how often have you had any pain?

1. Never
2. Some days
3. Most days
4. Every day
5. Prefer not to say

In the past 2 weeks, how often did pain limit your life or work activities? [only for participants who answer 2 -4 to preceding question]

1. Never
2. Some days
3. Most days
4. Every day
5. Prefer not to say

Recent Life Events

At baseline and at every two-week follow-up questionnaire, participants will be asked if they have experienced a range of recent life events. By assessing life events over the previous two weeks the questionnaire shall elucidate potential positive or negative circumstances which may impact depressive symptoms and mood.

For peer review only

In the past 2 weeks, have any of the following happened to you [tick all that apply]?

1. You entered a new romantic relationship
2. You had a mild illness (e.g., cough, cold, flu, fever)
3. You became seriously ill
4. You accomplished a personal achievement
5. You got into serious financial problems
6. You or your partner became pregnant
7. You started a new job
8. A romantic relationship ended
9. You took a holiday
10. A friend or family member died
11. You or your partner had a baby
12. You had a big argument with a friend or family member
13. You lost your job
14. No significant life events in the past 2 weeks
15. Other (please give details):
16. Prefer not to say

Treatment and Medication

Participants will be asked if they have received any of the following treatments for anxiety or depression: antidepressant medication, cognitive behavioural therapy, any other form of psychological therapy, or any self-help guidance. Participants will be asked to provide this information at baseline and at every two-week questionnaire following this.

In the past 2 weeks, have you taken or received any of the following? Select all that apply:

1. Antidepressant medication for anxiety or depression
2. Cognitive behavioural therapy for anxiety or depression
3. Other forms of psychological therapy for anxiety or depression
4. Any self-help guidance for anxiety or depression
5. None of the above
6. Prefer not to say

Current and Previous Mental Health

Participants will be asked about current and previous mental health diagnoses. Participants will be asked about this at baseline only.

Have you ever been diagnosed with a mental health condition by a health-care professional (e.g., doctor, psychiatrist, therapist, nurse, healthcare professional with specialist training)?

For peer review only

1. Yes, I have a current mental health diagnosis.
2. Yes, I have had a past mental health diagnosis.
3. Yes, I have **both a previous and current** mental health diagnosis.
4. No, I have never received a mental health diagnosis.
5. I'm not sure.
6. Prefer not to say

Please select which mental health diagnosis [select all that apply]

[Only for participants who answer 'Yes' to even been diagnosed with a mental health condition]

1. Depression

2. Anxiety

3. Phobia

4. Bi-polar disorder

4. Body dysmorphia

5. Premenstrual dysphoric disorder

6. Post Traumatic Stress Disorder

7. Obsessive Compulsive Disorder

8. An eating disorder

9. Psychosis

10. Schizophrenia or schizoaffective disorder

11. A personality disorder

12. Other (not listed)

Questionnaire and Study Feedback

Following the completion of the EMA study at six weeks participants will be asked to provide feedback on their experience of using the m-Path application and of participation in the study in general.

For peer review only

Helplines

The helplines below will be shown at the end of every daily survey and at the end of every questionnaire. Additionally, if someone answers the PHQ-9 with a positive score of ≥1 they will immediately be shown the helplines below before being able to progress with the survey.

- **SAMARITANS** - Emotional support for everyone
  Website: https://www.samaritans.org/scotland/ | *Tel:* 116 123 (24 hours)
- **MIND** - Advice and support for anyone with a mental health problem
  Website: https://www.mind.org.uk | *Tel:* 0300 123 3393 Text: 86463
- **SHOUT** - Support around self-harm
  Website: https://giveusashout.org/get-help/issues/help-self-harm/ | *Text:* 85258
- **NHS 111** - If you need medical help right now, 111 online can tell you what to do next | *Website:* https://111.nhs.uk.

For peer review only
